# Azobenzene‐Cored Amphiphilic Dendrimers: Molecular Photoswitching Controls Dendriplex Topology and Gene Delivery

**DOI:** 10.1002/smsc.70377

**Published:** 2026-08-12

**Authors:** María J. Puerto‐Madorrán, Gonzalo Rivero‐Barbarroja, Juan M. Benito, Stéphane Maisonneuve, Itziar Vélaz, Christophe Di Giorgio, Conchita Tros de Ilarduya, Francisco Mendicuti, Carmen Ortiz Mellet, Juan Xie, José M. García Fernández

**Affiliations:** ^1^ Instituto de Investigaciones Químicas (IIQ) CSIC – Universidad de Sevilla Seville Spain; ^2^ Photophysique et Photochimie Supramoléculaires et Macromoléculaires Université Paris‐Saclay, ENS Paris‐Saclay, CNRS Gif‐sur‐Yvette France; ^3^ Department of Organic Chemistry Faculty of Chemistry University of Seville Seville Spain; ^4^ Department of Chemistry School of Sciences University of Navarra Pamplona Spain; ^5^ Institute de Chimie de Nice, UMR 7272 Université Côte d’Azur Nice France; ^6^ Department of Pharmaceutical Sciences School of Pharmacy and Nutrition University of Navarra Pamplona Spain; ^7^ Departamento de Química Analítica Química Física e Ingeniería Química and Instituto de Investigación Química “Andrés del Rio” Universidad de Alcalá Madrid Spain

**Keywords:** azobenzene, dendrimers, gene delivery, photoswitching, supramolecular assembly

## Abstract

Azobenzene‐cored ionizable amphiphilic Janus dendrimers (Azo‐IAJDs) are introduced as molecularly defined, single‐component vectors in which reversible photoswitching controls plasmid DNA (pDNA) condensation pathways, dendriplex topology, and transfection behavior. The amphiphiles combine asymmetrically substituted azobenzene cores with ionizable and lipophilic dendrons and undergo efficient, reversible *E*/*Z* photoisomerization under biologically relevant conditions. In the presence of pDNA, they form nanocomplexes displaying markedly different supramolecular organizations depending on the architecture of the ionizable domain, ranging from highly ordered lamellar nanoparticles to morula‐like or weakly structured condensates. Molecular mechanics and molecular dynamics simulations reveal that photoisomerization within preassembled lamellar dendriplexes introduces local structural frustration, transiently destabilizes bilayer organization, and enhances amphiphile dissociation. These structural perturbations correlate with enhanced intracellular delivery and transgene expression upon *E *↔ *Z* interconversion. In vitro, *Z*‐derived nanocomplexes systematically outperform the corresponding *E*‐isomers in COS‐7, HepG2, and RAW264.7 cells, while ex vivo photoswitching yields up to 50‐fold increases in expression. In vivo studies further demonstrate isomer‐dependent passive organ targeting, including pronounced shifts from lung‐ to liver‐dominant transfection profiles. Collectively, these results establish molecular photoswitching as a mechanism to encode supramolecular DNA organization and biological function in single‐component dendritic vectors.

## Graphical abstract

Light switches gene delivery: Optomechanical actuation of multilamellar pDNA nanocomplexes formulated with single‐component azobenzene‐scaffolded ionizable Janus dendrimers (Azo‐IAJDs) enhances light‐regulated transfection in vitri and in vivo. The initial *E* or *Z* state programs cell selectivity and passive organ targeting without added ligands.

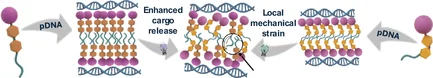

## 1 Introduction

Understanding how molecular‐level structural information propagates into supramolecular organization and biological function remains a central challenge in adaptive nanoscience [1, 2, 3]. This question is particularly relevant in nucleic acid (NA) delivery, where transfection efficiency depends not only on vector–cargo affinity but also on the topology, internal organization, and dynamic behavior of the resulting nanoassemblies [4, 5, 6]. Viral vectors continue to provide highly efficient gene transfer but remain limited by immunogenicity, risks of genomic integration, and manufacturing complexity [7]. Nonviral systems, particularly lipid nanoparticles (LNPs), have emerged as clinically validated alternatives and played a decisive role in the development of mRNA therapeutics [8, 9, 10]. Nevertheless, their intrinsic compositional heterogeneity and polydispersity complicate mechanistic interpretation and hinder the establishment of precise structure–activity relationships [11, 12, 13, 14]. Alternatively, molecularly defined vectors efficient in monoformulation provide a unique opportunity to directly correlate molecular architecture, supramolecular organization, and biological performance [15, 16, 17, 18, 19].

Stimuli‐responsive assemblies further expand this framework by enabling dynamic regulation of vector organization and intracellular processing [20]. Among external triggers, light is particularly attractive because it offers remote addressability together with excellent spatial and temporal resolution [21]. Azobenzene‐based photoswitches are especially appealing in this context due to their rapid and reversible *E*/*Z* photoisomerization, which predictably modifies molecular geometry, polarity, and intermolecular interactions [22, 23]. Such properties have enabled the development of photoresponsive systems capable of regulating oligonucleotide hybridization [24], DNA intercalation [25], ATP aggregation [26], G‐quadruplex stabilization [27], nucleic acid condensation [28], and gene expression [29]. Yet, despite these advances, examples in which molecular photoswitching within structurally defined vectors propagates hierarchically into supramolecular nucleic acid organization and biological transfection behavior remain exceedingly scarce [30].

Most photoresponsive NA‐delivery systems reported to date rely on polymeric or compositionally complex materials in which azobenzene motifs are incorporated as auxiliary responsive elements [31, 32, 33, 34, 35]. Although these systems successfully modulate bulk or colloidal properties, the inherent structural heterogeneity of the assemblies often obscures the mechanistic relationship between molecular switching, nanoscale organization, and biological function. We reasoned that molecularly defined amphiphiles capable of undergoing reversible geometrical reorganization upon photoisomerization could instead encode distinct pDNA condensation pathways and assembly morphologies. In particular, if photoswitching occurred within ordered DNA/amphiphile nanoassemblies, the resulting conformational perturbation could introduce localized mechanical strain, transiently destabilize internal organization, and facilitate nucleic acid release [36, 37, 38].

This concept finds support in previous studies on azobenzene‐containing supramolecular and soft materials, where collective *E*/*Z* photoisomerization induces large‐scale structural reorganization, phase transitions, and mechanical responses [39, 40]. Related effects have also been reported for azobenzene‐functionalized lipid bilayers and vesicles, in which photoisomerization perturbs membrane organization and transiently destabilizes supramolecular order [41, 42, 43, 44, 45]. Importantly, nucleic acid complexes formed by multihead/multitail amphiphiles frequently adopt lamellar architectures composed of alternating amphiphile bilayers and DNA monolayers [46, 47]. Such confined and highly ordered nanoenvironments are particularly attractive for amplifying molecular‐level conformational changes into supramolecular responses. We therefore hypothesized that photoisomerization within these assemblies could promote transient dendriplex swelling, local structural disorder, and partial amphiphile dissociation, thereby facilitating intracellular pDNA release.

In contrast to multicomponent formulations in which photoresponsive motifs operate as auxiliary structural elements, the systems investigated here were conceived as molecularly defined single‐component vectors capable of simultaneously encoding reversible photoswitching, amphiphilic self‐assembly, nucleic acid condensation behavior, and biological delivery function within the same compact molecular architecture. Within this framework, multivalent ionizable amphiphiles constitute especially attractive platforms because their modular architecture enables precise tuning of charge density, hydrophobic packing, and interfacial organization [12, 48, 49, 50]. In addition, incorporation of hydrogen‐bond donor functionalities such as thiourea or triazole groups can reinforce phosphate recognition while maintaining dynamic and reversible nucleic acid complexation [51, 52, 53, 54]. In particular, aminothiourea motifs are expected to promote adaptive electrostatic and hydrogen‐bonding interactions with the phosphate backbone while avoiding excessively kinetically trapped DNA condensates [55]. Multihead/multitail Janus architectures built on relatively simple aromatic or aliphatic scaffolds are also known to favor the formation of highly ordered lamellar assemblies [18, 19, 56, 57, 58, 59]. These precedents prompted us to investigate whether azobenzene‐driven molecular reorganization could be translated into topology‐dependent nucleic acid condensation and transfection behavior within structurally defined dendritic vectors.

**FIGURE 1:**
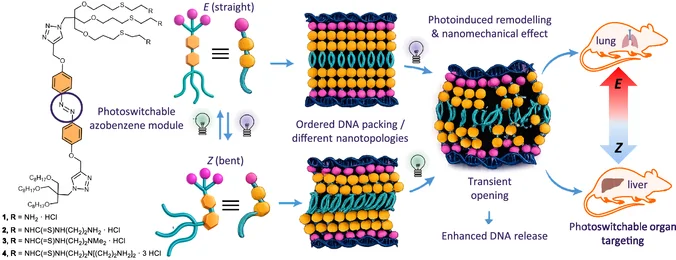
Structure of the Azo‐IAJDs prepared in this work (**1–** **4**) and schematic representation of the Azo‐IAJD concept: In the presence of plasmid DNA (pDNA), the multihead/multitail Azo‐IAJDs can self‐assemble into lamellar, lipid bilayer‐like architectures both in the *E* and *Z* state, with distinct cell and organ‐ transfection selectivities; light‐promoted (*E *→ *Z* or *Z *→ *E*) interconversion can generate nanomechanical perturbation, triggering transient destabilization of the supramolecular structure, facilitating pDNA release.

Herein, we introduce azobenzene‐based ionizable amphiphilic Janus dendrimers (Azo‐IAJDs) as single‐component, photoswitchable vectors for plasmid DNA (pDNA) delivery. The molecular design combines an asymmetrically substituted azobenzene core with ionizable and lipophilic dendrons connected through triazole‐containing branching units. Reversible *E*/*Z* photoisomerization modulates the relative disposition of the cationic and hydrophobic domains while preserving the ability of both isomeric states to form stable pDNA nanocomplexes. Depending on the architecture of the ionizable domain, the resulting dendriplexes display markedly different supramolecular organizations, ranging from highly ordered lamellar nanoparticles to morula‐like or weakly structured condensates. Combined experimental and computational analyses further reveal that photoisomerization within preassembled nanocomplexes induces localized nanomechanical perturbation, transient destabilization of lamellar order, and enhanced amphiphile dissociation. These effects translate into pronounced differences in transfection efficiency and passive organ targeting in vitro and in vivo (Figure 1).

pDNA was selected in the present work as a particularly demanding and structurally informative cargo because successful transfection requires not only efficient condensation and cellular uptake but also endosomal escape, cytosolic trafficking, nuclear transport, and transcriptional activation. Accordingly, pDNA provides a stringent model for evaluating whether molecular photoswitching can effectively propagate into supramolecular remodeling and biologically measurable delivery outcomes.

Importantly, the conceptual framework explored here differs fundamentally from conventional photoactivated “on/off” delivery systems that require irradiation after administration. In the present case, both *E* and *Z* isomeric states remain capable of forming stable and transfection‐competent dendriplexes while exhibiting distinct supramolecular organizations and biological behaviors. Consequently, photoswitching can be performed ex vivo prior to administration, enabling modulation of transfection efficiency and passive organ selectivity without the need for in vivo irradiation. This distinction is particularly relevant in view of the limited tissue penetration and potential phototoxicity associated with UV–visible activation. Overall, this work establishes molecular photoswitching as a mechanism to encode supramolecular nucleic acid organization and biological function in molecularly defined dendritic vectors.

## 2 Results and Discussion

### 2.1 Molecular Design and Synthesis

The azobenzene‐based ionizable amphiphilic Janus dendrimers (Azo‐IAJDs) **1–** **4** were designed as structurally defined vectors integrating (i) an asymmetrically substituted azobenzene core acting as the photoresponsive module, (ii) hydrophobic dendrons promoting amphiphile self‐assembly, and (iii) ionizable dendritic domains responsible for pDNA complexation. Triazole‐ and aminothiourea‐containing branching motifs were additionally incorporated to reinforce phosphate recognition through combined electrostatic and hydrogen‐bonding interactions.

The molecular architecture depicted in Figure 1 was inspired by the tendency of multihead/multitail aromatic amphiphiles to organize into lamellar nucleic acid assemblies composed of alternating amphiphile bilayers and DNA monolayers. Within this framework, incorporation of an azobenzene core was expected to enable reversible modulation of the relative spatial disposition of the ionizable and hydrophobic dendrons upon *E*/*Z* photoisomerization. Whereas the thermodynamically stable *E*‐isomer should favor a more extended molecular organization, the *Z*‐isomer was anticipated to adopt a more compact‐bent geometry potentially influencing amphiphile packing, bilayer curvature, and dendriplex internal organization. Across the series, the hydrophobic domain was intentionally conserved to preserve comparable self‐assembly tendencies [18, 19, 60], whereas the ionizable domain was systematically diversified to modulate charge density and interfacial organization [60, 61, 62]. Compounds **1** and **2** incorporate terminal primary amines, **3** contains tertiary aminoethylthiourea motifs, and **4** bears a more densely functionalized polyamine architecture. This design enabled evaluation of how relatively subtle modifications in ionizable headgroup topology influence pDNA co‐assembly, dendriplex organization, and transfection behavior while maintaining the same azobenzene‐centered photoswitchable scaffold.

**SCHEME 1:**
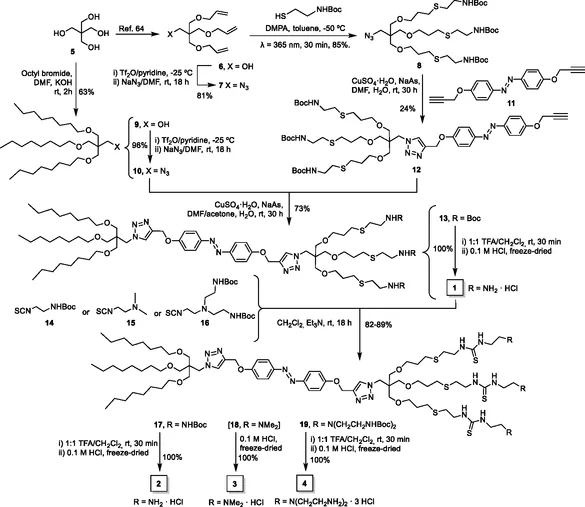
Synthesis of the Azo‐IAJDs **1** **–4**.

Compounds **1–** **4** were synthesized through a modular convergent strategy combining three orthogonal click‐type transformations: thiol‐ene radical addition to generate thioether linkages [63], copper(I)‐catalyzed azide–alkyne cycloaddition (CuAAC) to form 1,4‐disubstituted 1H‐1,2,3‐triazoles [64], and amine‐isothiocyanate coupling to afford *N*,*N′*‐disubstituted thioureas [65] (Scheme 1). The ionizable and lipophilic dendritic precursors were assembled from pentaerythritol (**5**) through sequential triple *O*‐allylation (→**6**) [66] or *O*‐octylation (→**9**), triflation/azidation (→**7** and **10**) and, for the first one, the incorporation of Boc‐protected cysteaminyl segments (→**8**). Sequential CuAAC coupling of the 4,4′‐bis(propargyloxy)azobenzene (**11**) [67] with azide‐functionalized dendrons **7** (→**12**) and **10** (→**13**) afforded the corresponding Janus intermediates, which after Boc deprotection yielded the triamine derivative **1**. Subsequent reaction with the corresponding isothiocyanates **14** [68], **15**, or **16** [69] furnished thiourea derivatives **2–** **4** after final deprotection and lyophilization from diluted HCl. All compounds were isolated as polyhydrochloride salts and fully characterized by NMR spectroscopy, mass spectrometry, and elemental analysis (see Sections S1‐S3).

### 2.2 Photoresponse and Molecular Reorganization of the Azo‐IAJDs

The photochromic properties of Azo‐IAJDs **1** **–4** were examined in 9:1 (v/v) water–DMSO, a medium compatible with both nucleic acid (NA) complexation and transfection studies. Particular attention was devoted not only to assessing the efficiency and reversibility of *E *⇄ *Z* photoisomerization after incorporation of the azobenzene unit into the dendritic scaffold but also to evaluating the persistence of the metastable *Z* state under biologically relevant conditions (see Section S4 for details).

**FIGURE 2:**
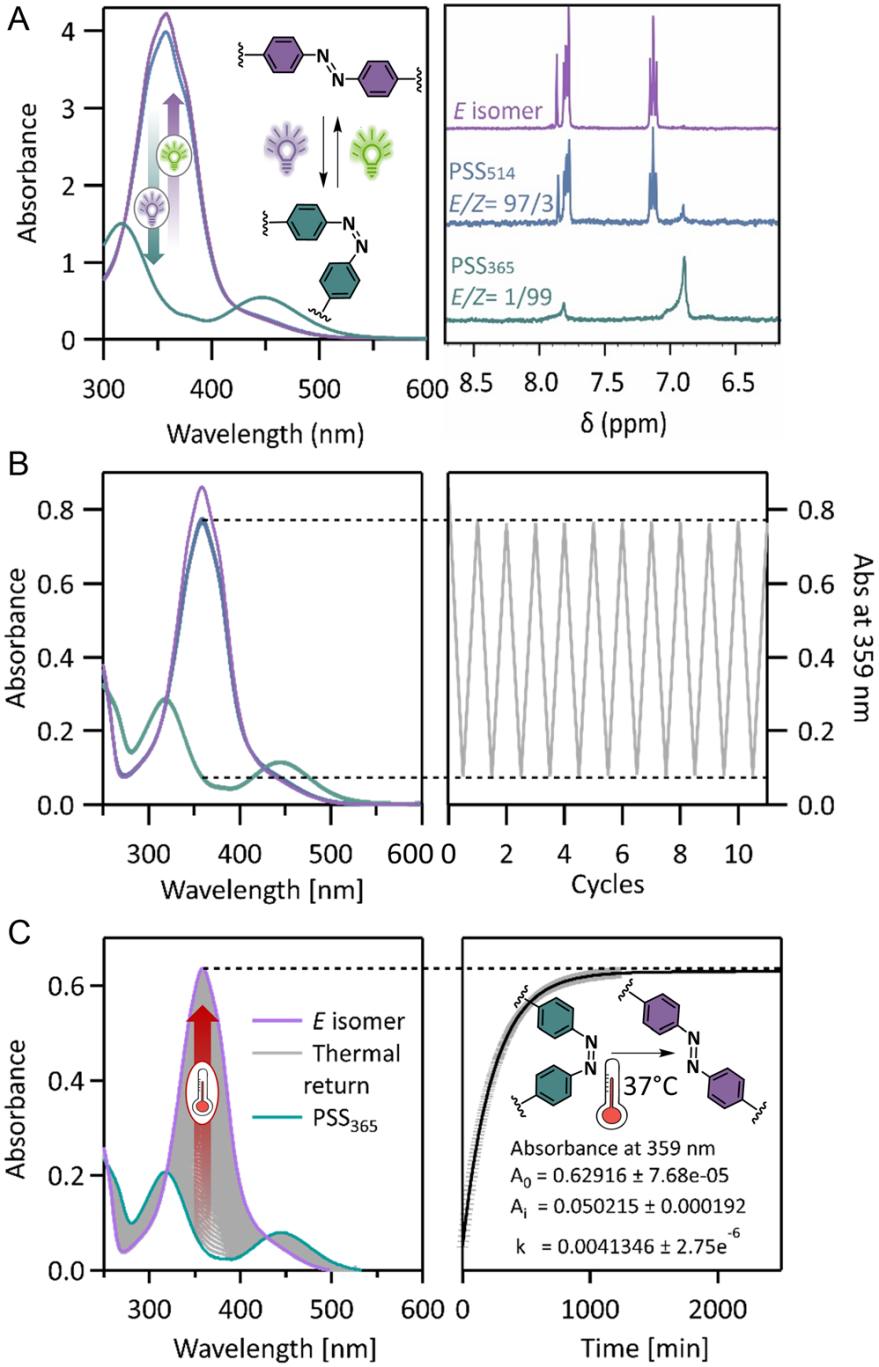
Photochromic properties of Azo‐IAJD **1** as a representative example. (A) Aromatic region in the ^1^H NMR spectra, recorded in 1:9 DMSO‐*d*_6_‐D_2_O, of the *E*‐isomer (purple line), PSS_365_ (major *Z*‐isomer; green line), and PSS_514_ (major *E*‐isomer; blue line). (B) Fatigue resistance monitored by UV–vis absorption in 1:9 DMSO‐*d*_6_‐D_2_O, showing the plot of the maximum absorption at 357 nm (*E*‐isomer) or 363 nm (*Z*‐isomer) under alternate irradiation at 365 nm (45 s, *P*_365_ = 92.00 mW cm^−2^) and 514 nm (660 s; *P*_514_ = 19.15 mW cm^−2^). (C) UV–vis absorption spectra of the thermal return (TR) in DMSO at 37 °C and curve fitting following the absorbance at 359 nm according to equation: Abs (*t*) = *A*_0_ − (*A*_0_ − *A* *_i_*)e^(−*kt*)^, where *A*_0_ is the absorption of the *E*‐isomer, *A* *_i_* is the absorption at PSS_365_, *k* is the rate constant of thermal isomerization, and *t* is the time in min; the half life (*τ*_1/2_) is calculated according to the equation *τ*_1/2_ = ln(2)/*k*. See the Supporting Information for details.

Thermally equilibrated solutions, containing exclusively the *E*‐isomer, displayed an intense π → π* absorption band at 357–360 nm. Irradiation at 365 nm generated a photostationary state (PSS_365_) in which this band was strongly attenuated and a new n → π* transition appeared at 444–449 nm, consistent with formation of the *Z*‐isomer. Subsequent irradiation at 514 nm restored the *E* configuration almost quantitatively (96%–99%). Well‐defined isosbestic points indicated a clean two‐state photoisomerization process, while *E*/*Z* ratios determined from the corresponding ^1^H NMR spectra confirmed efficient photoconversion. Alternating irradiation cycles further demonstrated excellent fatigue resistance without detectable degradation (Figure 2A,B). The thermal stability of the metastable *Z*‐isomers was evaluated at 37 °C by monitoring recovery of the *E*‐isomer absorption band in the dark (Figure 2C). Compounds **1**‐**3** displayed *Z *→ *E* half‐lives (*τ*_1/2_) ranging from 120 to 168 min, in agreement with related 4,4′‐alkoxy‐substituted azobenzenes [70, 71, 72, 73]. In contrast, compound **4**‐*Z* relaxed more rapidly, with a *τ*_1/2_ approximately fivefold shorter. This accelerated relaxation may arise from transient intramolecular proton‐transfer processes involving the more densely cationic headgroup of **4**, which could reduce the N=N double‐bond character and lower the rotational barrier for isomerization [74, 75].

Importantly, *Z *→ *E* relaxation is expected to slow considerably within organized supramolecular environments such as bilayers or NA nanocomplexes, where steric confinement restricts molecular mobility [76, 77]. Consistently, DNA binding has previously been shown to retard thermal relaxation of cationic azobenzene amphiphiles by more than one order of magnitude [36]. The *Z* forms of **1–** **4** can therefore be expected to remain sufficiently persistent within Azo‐IAJD/pDNA nanocomplexes during the time‐window relevant to cellular uptake, allowing meaningful assessment of isomer‐dependent transfection behavior. These experiments further demonstrate that the azobenzene core remains an effective molecular switch after incorporation into the Janus dendritic scaffold. Because *E*/*Z* photoisomerization modifies both the polarity and geometry of the central azobenzene unit, it is expected to alter the relative disposition of the ionizable and lipophilic dendrons. In the context of pDNA‐templated self‐assembly, such conformational reorganization may influence amphiphile packing, charge screening, and phosphate recognition, thereby modulating dendriplex internal organization while preserving nanocomplex integrity. This feature is central to the present design, where photoswitching is intended to regulate assembly dynamics and supramolecular organization rather than simply trigger nanoparticle disruption.

### 2.3 Co‐Assembly of Azo‐IAJDs With pDNA and Characterization of the Nanocomplexes

The ability of Azo‐IAJDs **1**–**4**, in both thermally equilibrated *E* and photoinduced *Z*‐enriched forms (>95% *Z* at PSS_365_), to condense and protect pDNA was evaluated using the firefly luciferase‐encoding plasmid pCMV‐LucVR1216. Nanocomplexes were prepared at cationizable nitrogen‐to‐phosphorus (N/P) ratios of 5, 10, and 20 in HEPES buffer (10 mM, pH 7.4) under dark conditions (see Section S5 and Table S3 for details).

Dynamic light scattering (DLS) measurements (Section S6) revealed the formation of self‐assembled nanoparticles with hydrodynamic diameters (*D*_h_) ranging from 70 to 125 nm, low polydispersity indices (PDI < 0.3), and positive ζ‐potentials (Table 1), values well within the range generally considered suitable for gene‐delivery formulations. Deviations from this behavior were mainly observed for nanocomplexes prepared at low N/P ratios using the tertiary amine derivative **3** and, to a lesser extent, derivative **4**, which produced larger and more polydisperse assemblies indicative of incomplete pDNA compaction.

**TABLE 1:** Average hydrodynamic diameter (*D*_h_), polydispersity index (PDI), and ζ‐potential values for nanocomplexes formulated with pCMV‐LucVR1216 pDNA and Azo‐IAJDs **1**–**4**.

Compound	N/P	*D*_h_, nm	PDI	ζ‐potential, mV
**1**‐*E*	5	117 ± 1	0.231 ± 0.010	+19.6 ± 0.5
	10	104 ± 1	0.270 ± 0.050	+25.3 ± 1.1
	20	81 ± 1	0.132 ± 0.026	+29.6 ± 0.2
**1**‐*Z*	5	80 ± 1	0.190 ± 0.001	+16.7 ± 1.5
	10	77 ± 1	0.199 ± 0.002	+23.1 ± 1.9
	20	72 ± 2	0.254 ± 0.001	+22.6 ± 3.3
**2**‐*E*	5	103 ± 2	0.225 ± 0.035	+21.2 ± 0.7
	10	92 ± 1	0.164 ± 0.001	+26.1 ± 0.7
	20	87 ± 1	0.152 ± 0.013	+31.5 ± 1.6
**2**‐*Z*	5	771	0.195 ± 0.004	+19.9 ± 1.1
	10	79 ± 1	0.244 ± 0.057	+20.9 ± 0.2
	20	73 ± 2	0.250 ± 0.002	+25.9 ± 0.7
**3**‐*E*	5	707 ± 91	1.000 ± 0.014	+22.1 ± 1.0
	10	142 ± 3	0.348 ± 0.019	+21.0 ± 0.6
	20	112 ± 1	0.163 ± 0.005	+37.8 ± 1.4
**3**‐*Z*	5	425 ± 34	0.510 ± 0.110	+14.2 ± 0.5
	10	125 ± 1.	0.148 ± 0.091	+22.1 ± 0.6
	20	110 ± 1	0.109 ± 0.004	+31.3 ± 3.6
**4**‐*E*	5	155 ± 11	0.473 ± 0.030	+17.7 ± 0.7
	10	97 ± 1	0.166 ± 0.004	+23.0 ± 1.0
	20	85 ± 2	0.316 ± 0.002	+26.5 ± 1.3
**4**‐*Z*	5	138 ± 3	0.285 ± 0.112	+19.0 ± 0.5
	10	86 ± 1	0.187 ± 0.049	+25.0 ± 1.7
	20	74 ± 1	0.197 ± 0.035	+22.5 ± 3.6

Clear structure‐dependent trends emerged across the Azo‐IAJD series. For compounds **1–** **3**, each incorporating one amino group per branch, *Z*‐derived formulations systematically generated smaller nanocomplexes than the corresponding *E*‐derived systems at identical N/P ratios, whereas *E*‐derived dendriplexes generally exhibited higher ζ‐potentials, particularly at N/P = 20. This behavior suggests more efficient charge screening within the *Z*‐derived assemblies, likely reflecting differences in amphiphile packing arising from the more compact bent geometry of the *Z*‐isomer. In contrast, the more densely functionalized derivative **4** displayed weaker correlations between azobenzene configuration and nanoparticle physicochemical parameters.

**FIGURE 3:**
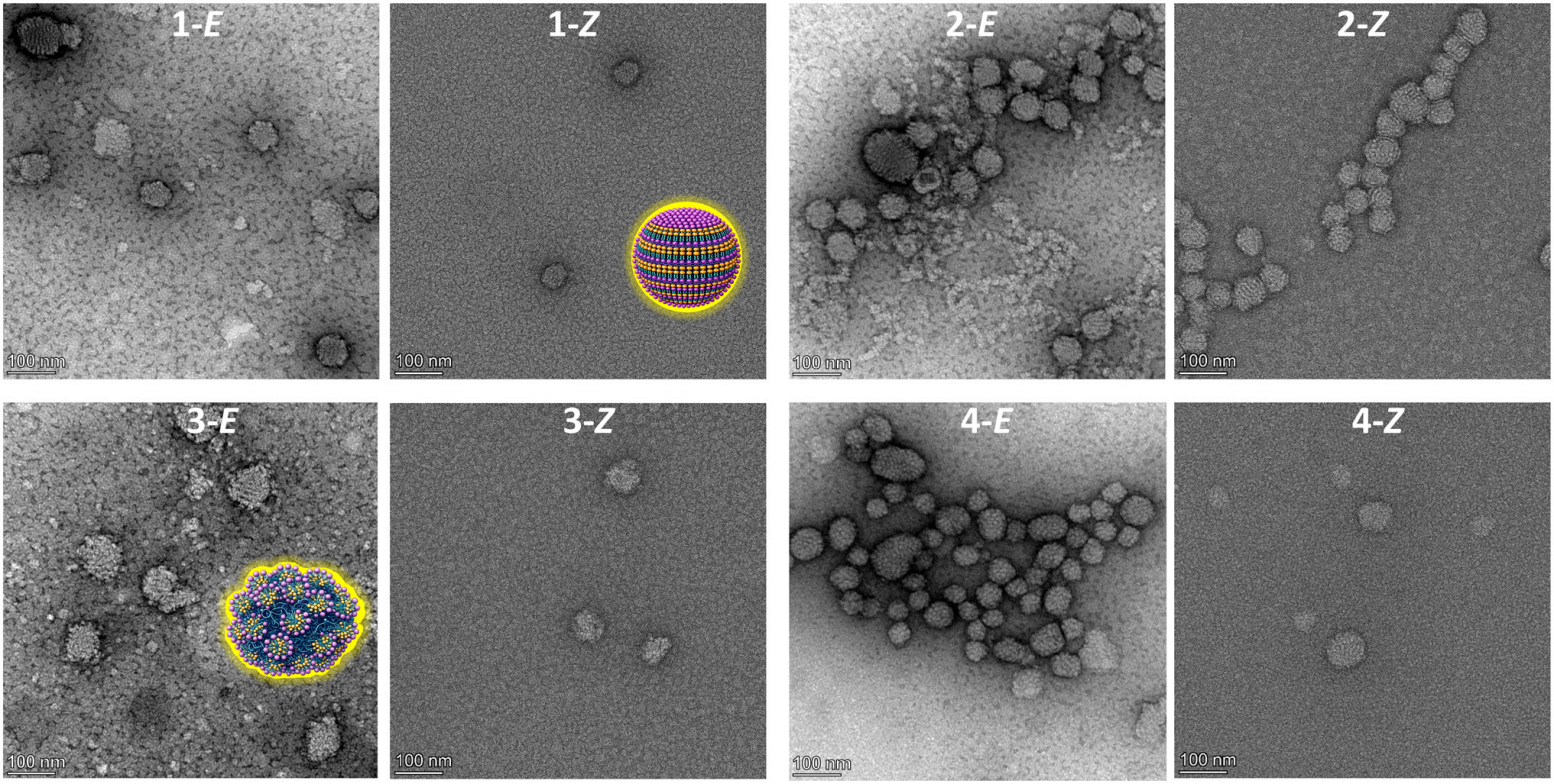
Representative TEM micrographs of N/P 20 Azo‐IAJD **1–** **4**/pDNA nanocomplexes, formulated with either the *E* or the predominant *Z* configuration of the vector, as indicated. Schematic representations of lamellar and morula‐like arrangements are also shown.

Transmission electron microscopy (TEM; Section S9) revealed that relatively subtle variations in ionizable headgroup architecture translate into major differences in dendriplex morphology and internal organization (Figure 3). Compounds **1** and **2**, both incorporating terminal primary amines, generated homogeneous spherical nanoparticles displaying pronounced long‐range lamellar organization characterized by alternating dark and light regions, consistent with interleaved pDNA monolayers and Azo‐IAJD bilayers [78]. In contrast, tertiary amine‐containing derivative **3** produced less‐ordered morula‐like nanocomplexes with irregular internal organization, whereas the more hydrophilic analogue **4** yielded ellipsoidal‐to‐spheroidal nanoparticles displaying only short‐range order. Compared with the strong influence exerted by ionizable headgroup architecture, the azobenzene configuration itself produced comparatively more subtle effects on overall nanoparticle morphology. *E*‐ and *Z*‐derived assemblies generally preserved similar global organization, although *Z*‐derived particles consistently appeared slightly smaller and structurally better defined, in excellent agreement with the DLS results.

Electrophoretic mobility shift assays (EMSA) confirmed complete pDNA complexation and efficient protection against enzymatic degradation. In all cases, plasmid migration was fully suppressed, whereas subsequent treatment with DNase I followed by SDS addition restored intact pDNA bands, demonstrating efficient shielding of the genetic cargo from nuclease attack (Figures S31 and S32). DLS measurements further revealed negligible variation in *D*_h_ after storage for 6 weeks at 4 °C or 24 h at 25 °C in the dark, indicating excellent colloidal stability (Section S8 and Figures S31 and S32).

Taken together, these results demonstrate that Azo‐IAJDs efficiently condense pDNA into stable supramolecular nanocomplexes irrespective of the azobenzene isomeric state, while simultaneously revealing a strong dependence of dendriplex internal organization on ionizable dendritic architecture. Particularly noteworthy is the emergence of highly ordered lamellar assemblies for compounds **1** and **2**, which appear especially well suited to amplify molecular‐level conformational perturbations into larger supramolecular responses.

We therefore investigated whether *E *⇄ *Z* photoisomerization could induce nanostructural remodeling within preassembled dendriplexes. Combined UV–vis/DLS measurements performed on N/P 10 and 20 nanocomplexes subjected to alternating 365/466 nm irradiation cycles confirmed that photoisomerization proceeds efficiently within intact nanoparticles. Isomer interconversion induced moderate nanoparticle swelling, reflected by increases in *D*_h_ and PDI together with significant decreases in ζ‐potential, yet without detectable precipitation or irreversible aggregation (Section S7 and Table S4). These observations support a reversible nanostructural remodeling mechanism in which molecular photoswitching transiently perturbs dendriplex internal organization while preserving overall colloidal integrity. Rather than inducing irreversible particle disruption, photoswitching primarily modulates internal organization and dynamic permeability within the nanocomplexes. Such transient structural perturbation is expected to facilitate intracellular pDNA release under biological conditions.

Together, these observations support a model in which repeated *E *↔ *Z* photoisomerization does not induce catastrophic dendriplex disassembly but instead promotes transient nanostructural remodeling within the preassembled nanocomplexes. The moderate swelling together with the pronounced reduction in ζ‐potential observed upon alternate irradiation are fully consistent with partial relaxation of the compact electrostatic organization, enhanced permeability, and increased pDNA accessibility. These findings experimentally support the optomechanical mechanism proposed from the computational analysis (see hereinafter).

### 2.4 Computational Analysis of Photoinduced Nanostructural Remodeling in Azo‐IAJD/pDNA Nanocomplexes

To gain molecular‐level insight into the effect of azobenzene photoisomerization on dendriplex organization and stability, molecular mechanics (MM) and molecular dynamics (MD) simulations were performed using compound **1** as a representative system, since it forms highly ordered lamellar nanocomplexes in both *E* and *Z* configurations. The computational workflow comprised: (i) minimization of isolated *E* and *Z* monomers; (ii) construction of 3 × 3 bilayers of **1**‐*E* and **1**‐*Z*; (iii) evaluation of their interactions with two DNA dodecanucleotides (CGCGAATTCGCG); and (iv) assessment of the consequences of partial or complete *E *→ *Z* or *Z *→ *E* switching within preassembled bilayer/DNA complexes (Section S14).

**FIGURE 4:**
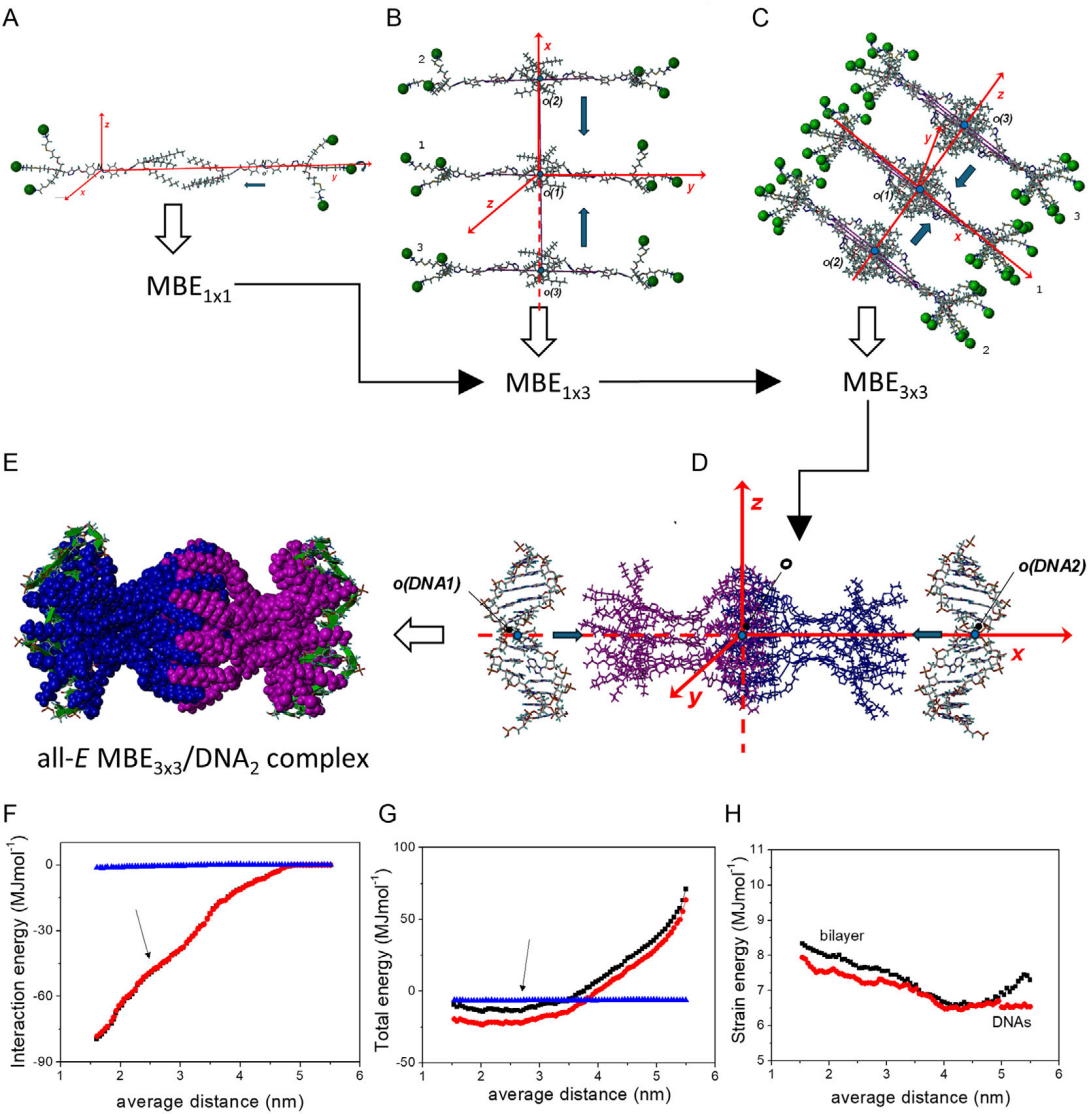
(A–C) Coordinate system used for approaching two Azo‐AIJD **1** molecules, in the *E*‐configuration, to form a 1 × 1 dimer, laterally approaching two dimers to a central one to form a 1 x 3 monolayer, and side‐by‐side approaching of two 1 × 3 monolayers to a central one to form a 3 × 3 bilayer, respectively. The workflow involved the corresponding minimum‐binding‐energy (MBE) structure at each step. (D) Schematic illustration of the coordinate system used for the simultaneous approach of two DNA fragments (CGCGAATTCGCG) to the three‐dimensional all‐*E* cationized 3 × 3 bilayer; the DNA fragments interact with the bilayer via their major groove. (E) Selected 3 × 3 bilayer/DNA_2_ MBE structure. (F) Total interaction energy (black), along with its electrostatic (red) and van der Waals (blue) components, between the DNA fragments and the **1** all‐*E* 3 × 3 bilayer, plotted as a function of the average of the distances between the centers of mass of the DNA1 and DNA2 fragments and the center of mass of the bilayer's hydrophobic domain. The arrow in the curve corresponds to the selected optimized structure in (E). (G,H) Same as in (F), showing the total interaction energy and its electrostatic and van der Waals components (using the same color scheme), along with the strain energy of both the DNA fragments (red) and the bilayer (black).

Initial bilayer models were generated through an iterative minimum‐binding‐energy (MBE) procedure. Explicit‐solvent MBE structures of **1**‐*E* and **1**‐*Z* homodimers (MBE_1×1_), arranged with their hydrophobic domains facing and the ammonium groups paired with chloride ions, were first constructed (Figure 4A). These units were subsequently assembled into larger 1 × 3 monolayers (MBE_1×3_; Figure 4B) and finally into complete 3 × 3 bilayers (MBE_3×3_; Figure 4C). van der Waals interactions dominated the stabilization energy, with longitudinal cohesion arising primarily from hydrophobic packing and lateral stabilization additionally benefiting from aromatic–aromatic interactions.

**FIGURE 5:**
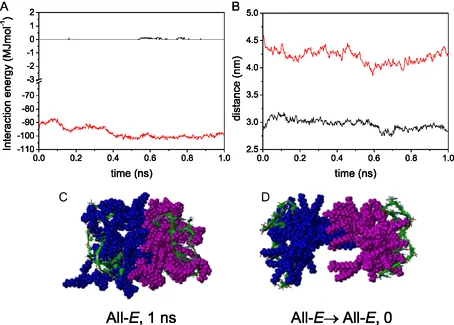
(A) Time evolution of the total interaction energy between the DNA fragments and the **1** all‐*E* 3 × 3 bilayer (red), and between the DNA1–DNA2 fragments (black), obtained from the analysis of 1‐ns MD trajectories in the presence of explicit water for the optimized bilayer–(DNA)_2_ nanocomplex. (B)Time evolution of the average of the *o–o′* distances (black) defined between the centers of mass of the two nitrogen atoms in each azobenzene unit from paired azobenzene groups within the bilayer and between the centers of mass of the DNA chains. (C) Snapshot of the **1** all‐*E* 3 × 3 bilayer/(DNA)_2_ nanocomplex at the end of the MD trajectory. (D) Structure obtained after isomerizing all monomers from the *E* to the *Z* configuration, showing nanocomplex dissociation already at 0 ns.

After chloride removal and further optimization, all‐*E* and all‐*Z* bilayers were positioned between two DNA fragments initially placed at noninteracting distances and progressively translated toward the cationic domains in 0.05 nm increments (Figure 4D). Each configuration was solvated, minimized, and analyzed to identify energetically favorable bilayer/DNA arrangements (Figure 5E). In all cases, the interaction energy became increasingly favorable upon approach, driven predominantly by electrostatic interactions between the cationic dendritic domains and the phosphate backbone of DNA (Figure 4F,G). However, at shorter distances the strain energy of both the bilayer and the DNA increased sharply (Figure 4H), indicating that excessive compression compromises structural stability. The optimized low‐energy structures selected from these calculations correspond to compromise configurations minimizing total and interaction energies while avoiding excessive strain penalties. These structures were subsequently re‐optimized and used as starting points for 1 ns MD simulations in explicit water (see Section S14 and Figures S35–S42 for details).

For the all‐*E* system, the MD trajectories revealed that both bilayer‐DNA and DNA–DNA interactions remained favorable throughout the simulation (Figure 5A), while the interlayer spacing and DNA–DNA distances remained relatively constant (Figure 5B). Snapshot analysis confirmed preservation of the lamellar organization over the entire trajectory (Figure 5C). Comparable behavior was observed for the all‐*Z* assemblies, in excellent agreement with the DLS, TEM, and EMSA experimental data demonstrating the stability of both *E*‐ and *Z*‐derived dendriplexes.

A markedly different picture emerged when partial photoisomerization was introduced within the bilayer organization. When nine of the 18 azobenzene units were manually switched to the opposite configuration, the monolayer interaction energy progressively approached 0 while the monolayer–monolayer distance increased, indicating progressive weakening of bilayer cohesion during the trajectory. Simulations in which 12 of the 18 azobenzene units were switched revealed even more pronounced destabilization, characterized by early loss of lamellar organization and larger interlayer separation. Although the effect was slightly attenuated when starting from the all‐*Z* state, destabilization remained evident in all cases (Figures S43–S46).

Finally, complete *E *→ *Z* or *Z *→ *E* conversion of all azobenzene units within the preassembled **1** 3 × 3 bilayer/(DNA)_2_ complexes was investigated. In both cases, isomerization introduced substantial local strain that severely disrupted the organization of the bilayer already at the beginning of the simulations, preventing convergence toward a new low‐energy MBE structure (Figures 5D and S47). The resulting structures displayed partial amphiphile dissociation together with significant perturbation of the ordered lamellar arrangement.

Collectively, these simulations support a mechanism in which azobenzene photoisomerization generates localized nanostructural perturbations within otherwise stable dendriplexes, which is consistent with the observed topological effects in alternate irradiation experiments. Molecular switching transiently weakens amphiphile cohesion, increases local structural disorder, and facilitates partial dissociation of the supramolecular framework, thereby promoting DNA disengagement and cargo release. Although the present data strongly support transient nanoscale remodeling upon photoswitching, future time‐resolved structural studies will be necessary to fully characterize the kinetics and spatial heterogeneity of the optomechanical perturbation process.

### 2.5 In Vitro Transfection Behavior

We next investigated whether the topology‐dependent supramolecular organization and nanostructural remodeling processes identified for the Azo‐IAJD/pDNA dendriplexes translate into measurable differences in biological transfection performance. COS‐7, HepG2, and RAW264.7 cells were transfected with nanocomplexes formulated from Azo‐IAJDs **1–** **4** and pCMV‐LucVR1216 at N/P ratios of 10 and 20 in the presence of 10% fetal bovine serum (FBS). Cells were incubated with the formulations for 4 h at 37 °C in the dark, followed by replacement with fresh complete medium and further incubation for 48 h prior to luciferase quantification. Branched polyethyleneimine (bPEI, 25 kDa; N/P = 10), SuperFect (SFect; SFect/pDNA mass ratio 10:1) and Lipofectamine 3000 (LP) were included as benchmark transfection controls (see Section S11 and Tables S6 and S7 for details).

**FIGURE 6:**
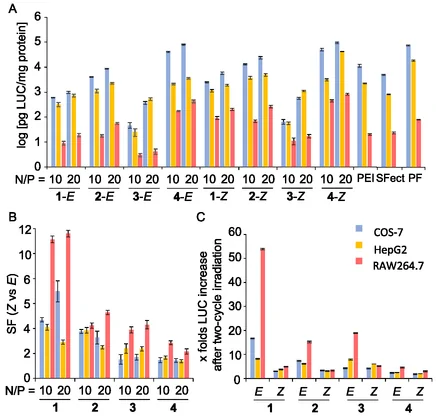
(A) Transfection efficiency in COS‐7, HepG2 and RAW264.7 cells for nanocomplexes formulated with the Azo‐IAJDs **1–** **4** in both the *E* or *Z* major form and the luciferase‐encoding plasmid pCMV‐Luc VR1216 at N/P 10 and 20, in the presence of 10% fetal bovine serum (FBS); data for control formulations using bPEI (PEI), SuperFect (SFect), and Lipofectamine 3000 (LP) are also shown. (B) Switching factor (SF) values, defined as the ratio between the transfection efficiency for the *Z* and the *E* isomer. (C) Relative transfection efficiency enhancement after a two alternating 5‐min irradiation cycles at 365 nm/466 nm (for *E* isomers) or 466 nm/365 nm (for *Z* isomers). The data represent the mean  ±  SD of three wells and are representative of three independent determinations.

For all Azo‐IAJDs, increasing the N/P ratio from 10 to 20 systematically enhanced transfection efficiency (Figure 6A), in agreement with the higher ζ‐potentials and improved pDNA compaction observed in the physicochemical characterization studies. Enhanced nanoparticle surface charge is expected to strengthen electrostatic interactions with negatively charged cellular membranes and promote uptake, while excess ionizable amphiphile may additionally facilitate destabilization of endosomal membranes and intracellular cargo release [79]. More importantly, pronounced structure‐dependent transfection profiles emerged across the Azo‐IAJD series. Introduction of aminothiourea segments in **2** relative to precursor **1** substantially enhanced transfection efficiency, particularly in COS‐7 cells, supporting the hypothesis that adaptive phosphate recognition and reversible complexation dynamics favor productive intracellular delivery. Replacement of the terminal primary amines in **2** by tertiary amino groups in **3** caused a marked reduction in transfection activity across all cell lines, whereas incorporation of mixed primary/tertiary amino functionalities in **4** restored or even improved performance. These differences correlate closely with the dendriplex supramolecular organizations and topologies revealed by TEM. Compounds **1** and **2**, which form highly ordered multilamellar nanoparticles, consistently mediated substantially higher transfection efficiencies than compound **3**, whose morula‐like assemblies display weaker internal organization. The more hydrophilic derivative **4**, despite lacking extensive long‐range lamellar order, still generated efficient transfection, likely reflecting the combined influence of compact particle size, favorable charge distribution, and enhanced colloidal adaptability. These observations indicate that transfection competence depends not simply on pDNA binding strength but rather on the emergence of suitable supramolecular organization and dynamic behavior within the dendriplexes.

The azobenzene configuration exerted an additional major influence on biological performance. In all cell lines and for all Azo‐IAJDs, *Z*‐derived formulations systematically mediated higher transfection levels than the corresponding *E*‐derived dendriplexes (Figure 6A and Table S6). With the exception of **3**‐*Z*, most *Z*‐derived nanocomplexes performed at levels comparable to or exceeding those obtained with bPEI or SFect controls, whereas **4**‐*Z* formulations approached the performance of Lipofectamine 3000. The corresponding switching factors (SF), defined as the ratio between luciferase expression obtained for *Z*‐ and *E*‐derived formulations of the same Azo‐IAJD, were consistently above unity and reached values between 2 and 12 in RAW264.7 cells (Figure 6B). These results indicate that relatively subtle molecular‐level geometrical changes associated with azobenzene *E*/Z isomerization propagate into substantial differences in biological function.

Importantly, the higher transfection efficiency of the *Z*‐derived systems cannot be simply attributed to improved cellular uptake resulting from smaller nanoparticle size. Although reduced hydrodynamic diameter may contribute to enhanced internalization, the observed differences likely also reflect changes in dendriplex internal organization and dynamic behavior. In particular, progressive thermal *Z*‐to‐*E* isomerization in the dark may provoke stronger local curvature fluctuations and enhanced structural adaptability within the supramolecular assembly, thereby facilitating endosomal escape and intracellular pDNA release. To directly probe the contribution of azobenzene isomerization‐mediated nanostructural remodeling, additional transfection experiments were performed in which the dendriplexes were irradiated after the cellular internalization stage but before the expression phase. For *Z*‐derived formulations, 15 min irradiation at 466 nm systematically enhanced luciferase expression by factors ranging from 1.9 to 4.0 for **1–** **3** and from 1.4 to 1.8 for **4** relative to nonirradiated controls. Even larger enhancements were observed when starting from *E*‐derived formulations and applying 365 nm irradiation, with increases between 1.8‐ and 10.2‐fold (Figure 6C). Alternating irradiation cycles produced even more pronounced effects. Two consecutive 5 min irradiation cycles at 365/466 nm (for *E*‐derived dendriplexes) or 466/365 nm (for *Z*‐derived dendriplexes) yielded transfection enhancements ranging from 2.2‐ to 53.9‐fold and from 1.7‐ to 6.0‐fold, respectively. In contrast, control formulations based on bPEI, SFect, or LP displayed no detectable photoresponse under identical conditions (see Table S6). These observations rule out a simple unidirectional mechanism whereby enhanced pDNA release would occur exclusively during *Z *→ *E* thermal relaxation. Instead, they support a model in which dynamic *E *⇄ *Z* interconversion itself transiently perturbs dendriplex internal organization, increases nanocomplex permeability, and facilitates partial amphiphile dissociation and intracellular cargo release.

Equally importantly, although alternating irradiation cycles partially erase the initial photoisomeric composition, the starting topology of the dendriplexes continues to influence the final transfection outcome. *E*‐ and *Z*‐derived nanocomplexes subjected to identical irradiation sequences still displayed significantly different transfection efficiencies, indicating that the supramolecular organization established during the initial condensation process exerts a lasting influence on cellular uptake, intracellular trafficking, and release behavior. Cytotoxicity evaluated by AlamarBlue assays revealed cell viabilities systematically above 80% under all experimental conditions (see Section S10, Table S5, and Figures S33 and S34), confirming that the enhanced transfection efficiencies are not associated with substantial membrane disruption or nonspecific toxicity.

In combination, these results demonstrate that Azo‐IAJD molecular design, dendriplex topology, and azobenzene photoisomerization cooperate to regulate transfection performance. More broadly, the findings support the concept that molecular photoswitching can modulate biological activity not through simple nanoparticle disassembly but rather by dynamically remodeling supramolecular nucleic acid organization and intracellular release pathways.

### 2.6 In Vivo Transfection and Passive Organ Targeting

The pronounced influence exerted by Azo‐IAJD molecular architecture and dendriplex organization on in vitro transfection behavior prompted us to investigate whether similar effects could also emerge under in vivo conditions. Particular attention was devoted to evaluating whether the conformational changes associated with azobenzene *E*/*Z* photoisomerization could propagate into measurable differences in passive organ targeting and transgene expression profiles.

**FIGURE 7:**
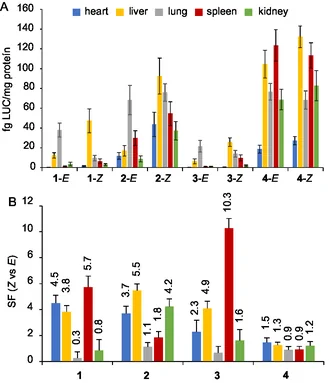
(A) Luciferase expression in the heart, liver, lungs, spleen, and kidney of mice after intravenous administration of 50 μg of pCMV‐Luc VR1216 formulated with **1–** **4**, in either the *E* or the *Z* major form. (B) Switching factor (SF) values, defined as the ratio between the luciferase expression for the *Z* and the *E* major isomer in each organ (right graph). Bars represent the mean ± SD (*n* = 8 animals).

BALB/c mice received intravenous injections of dendriplexes formulated with pCMV‐LucVR1216 using either thermally equilibrated *E*‐isomers or *Z*‐enriched formulations obtained by irradiation at 365 nm prior to complexation. Luciferase expression was quantified 24 h postadministration in the major organs following ex vivo tissue homogenization (Figure 7, Sections S12 and S13 and Table S8). The different Azo‐IAJDs displayed markedly distinct transfection profiles depending on both molecular structure and azobenzene configuration. Consistent with the in vitro results, compounds **1–** **3** generally exhibited lower transfection efficiencies than derivative **4**, whose dendriplexes mediated the highest levels of gene expression in most tissues examined. Beyond activity differences, substantial variations in passive organ‐targeting behavior were also observed across the series. The most striking example was provided by compound **2**. Whereas *E*‐**2**/pDNA dendriplexes preferentially promoted luciferase expression in the lungs, photoisomerization to the *Z* state produced a substantial redistribution toward liver‐associated transfection. Because both formulations possess identical chemical composition and differ only in azobenzene geometry, these observations demonstrate that conformational switching at the molecular level can ultimately translate into major differences in biological distribution behavior. A similar although less‐pronounced tendency was also observed for the other Azo‐IAJDs. In general, *Z*‐derived formulations displayed broader organ distribution together with enhanced liver‐associated expression relative to the corresponding *E*‐derived dendriplexes. These effects likely arise from differences in nanoparticle organization and dynamic behavior rather than from major variations in global physicochemical parameters, since DLS measurements revealed only moderate differences in Dh and ζ‐potential between *E*‐ and *Z*‐derived assemblies.

Importantly, the in vivo transfection profiles correlate closely with the internal organization of the corresponding dendriplexes observed by TEM. Highly ordered multilamellar nanoparticles formed by **1** and **2** displayed biological behavior markedly different from the less‐structured assemblies generated by **3**. Such results reinforce the conclusion that transfection performance is strongly influenced by dendriplex organization and structural adaptability rather than simply by pDNA‐binding affinity or nanoparticle size. The enhanced performance of *Z*‐derived formulations may additionally reflect increased structural flexibility associated with thermal isomerization into the *E*‐isomer. Molecular simulations together with alternating irradiation experiments suggested that *E *⇄ *Z* interconversion induces transient nanostructural perturbations within the dendriplexes, facilitating local rearrangement of the amphiphilic organization and partial weakening of intermolecular cohesion. Under biological conditions, such behavior may promote more efficient intracellular pDNA release. Collectively, these findings demonstrate that the consequences of azobenzene photoisomerization extend well beyond local molecular geometry changes and propagate hierarchically into supramolecular and functional outputs under physiological conditions.

## 3 Conclusions

This work demonstrates that Azo‐IAJDs constitute a unique class of molecularly defined, single‐component vectors in which reversible molecular photoswitching directs pDNA condensation pathways, dendriplex topology, and biological transfection behavior. By integrating a photoswitchable azobenzene core within amphiphilic dendritic architectures combining ionizable and lipophilic domains, it was possible to translate relatively small molecular‐level conformational perturbations into substantial supramolecular and functional consequences.

All Azo‐IAJDs efficiently underwent reversible *E *⇄ *Z* photoisomerization under biologically relevant conditions while preserving their ability to form stable pDNA nanocomplexes. Importantly, photoisomerization did not simply induce nanoparticle disassembly or gross colloidal destabilization. Instead, both photoisomeric states remained fully competent for pDNA condensation and self‐assembly, while displaying distinct supramolecular organization and dynamic behavior. Depending on the nature of the ionizable dendritic domain, the resulting dendriplexes ranged from highly ordered multilamellar nanoparticles to morula‐like or weakly structured condensates, demonstrating that relatively subtle molecular modifications can redirect nucleic acid condensation pathways toward profoundly different organizational states.

Significant relationships arose between dendriplex topology and biological performance. Compounds forming highly ordered lamellar assemblies consistently mediated the highest transfection efficiencies, whereas less‐ organized condensates displayed substantially lower activity despite efficient pDNA‐binding. These observations indicate that productive gene delivery depends not only on nucleic acid affinity but also on the emergence of suitable supramolecular organization and dynamic adaptability within the nanocomplexes.

The azobenzene configuration exerted an additional major influence on transfection behavior. *Z*‐derived dendriplexes systematically outperformed the corresponding *E*‐derived formulations in vitro, while alternating irradiation cycles further enhanced transgene expression. Combined experimental and computational analyses support a mechanism in which *E *⇄ *Z* interconversion within preassembled dendriplexes transiently perturbs internal amphiphilic organization, promotes local nanostructural remodeling, and facilitates intracellular pDNA release. Importantly, this behavior differs fundamentally from conventional photoresponsive delivery systems based on irreversible nanoparticle disruption or cargo uncaging. In the present systems, photoswitching dynamically modulates supramolecular organization while preserving overall colloidal integrity.

In vivo studies further demonstrated that molecular photoswitching can influence passive organ targeting and tissue‐selective transfection behavior. Particularly remarkable was the observation that conformational changes associated with azobenzene isomerization at the molecular level produced substantial redistribution of transgene expression between lung and liver tissues, highlighting the strong dependence of biological function on dendriplex topology and interfacial organization. Importantly, Azo‐IAJDs allow modulating cell and organ transfection selectivity through ex vivo photoisomerization. Nevertheless, we recognize that fully benefitting from the optomechanical transfection‐enhancing mechanism in translational applications would require photoswitches responsive to more biologically compatible wavelengths or adapted irradiation technologies. In this context, red‐ and near‐infrared‐responsive azobenzene derivatives, two‐photon activation strategies and upconverting nanoparticle‐assisted photoisomerization have emerged as promising approaches to improve tissue penetration while minimizing phototoxicity [80, 81, 82, 83]. Although such developments remain beyond the scope of the present study, the modular architecture of the Azo‐IAJDs should facilitate future implementation of these concepts.

Altogether, these findings highlight optomechanical regulation as a promising strategy to encode adaptive supramolecular nucleic acid organization and biological function in structurally defined dendritic vectors. More broadly, the work illustrates how molecular conformational changes can propagate hierarchically into topology‐dependent nanoscale and biological behaviors, providing a design framework for future stimuli‐responsive vectors.

## Funding

This study was supported by Ministerio de Ciencia, Innovación y Universidades (PID2022‐141034OB‐C21, PID2024‐157753OB‐C21, and PID2024‐157753OB‐C22), Agencia Estatal de Investigación (PID2022‐141034OB‐C21, PID2024‐157753OB‐C21, and PID2024‐157753OB‐C22), European Regional Development Fund, HORIZON EUROPE Marie Sklodowska‐Curie Actions (101130235 – Bicyclos), Consejería de Universidad, Investigación e Innovación Junta de Andalucia (DGP_PIDI_2024_00465), Red de Enfermedades Raras CSIC (RER‐CSIC), PTI+ Salud Global – CSIC, and Booster program, ENS Paris‐Saclay.

## Conflicts of Interest

The authors declare no conflicts of interest.

## Supporting information

The complete description of the materials, synthesis, photocharacterization, formulation, biological evaluation, computational methods, and additional data are provided in the Supporting Information. The authors have cited additional references within the Supporting Information.

## Data Availability

The data that support the findings of this study are available in the Supporting Information of this article.
