# Supplementary material for: Azobenzene‐Cored Amphiphilic Dendrimers: Molecular Photoswitching Controls Dendriplex Topology and Gene Delivery
Source: Small Sci. 2026 Aug 12;6(8):e70377. doi: 10.1002/smsc.70377 (PMC13489328; doi:10.1002/smsc.70377)
Supplement: Supplementary file 1 [file SMSC-6-e70377-s001.pdf]

Supporting Information for:

Azobenzene-Cored Amphiphilic Dendrimers: Molecular Photoswitching Controls Dendriplex Topology and Gene Delivery

María J. Puerto-Madorrán,[abc] Gonzalo Rivero-Barbarroja,[c] Juan M. Benito,[a] Stéphane Maisonneuve,[b] Itziar Vélaz,[d] Christophe Di Giorgio,[e] Conchita Tros de Ilarduya,[f] Francisco Mendicuti,[g] Carmen Ortiz Mellet*[c], Juan Xie*[b] and José M. García Fernández*[a]

[a] Instituto de Investigaciones Químicas (IIQ), CSIC – Universidad de Sevilla
Américo Vespucio 49, 41092 Seville, Spain
E-mail: jogarcia@iiq.csic.es

[b] Photophysique et Photochimie Supramoléculaires et Macromoléculaires
Université Paris-Saclay, ENS Paris-Saclay, CNRS
Gif-sur-Yvette 91190, France
E-mail: joanne.xie@ens-paris-saclay.fr

[c] Department of Organic Chemistry, Faculty of Chemistry, University of Seville
c/ Profesor García González 1, 41012 Seville, Spain
E-mail: mellet@us.es

[d] Department of Chemistry, School of Sciences, University of Navarra
31080 Pamplona, Spain

[e] Institute de Chimie de Nice, UMR 7272
Université Côte d'Azur
F-06108 Nice, France

[f] Department of Pharmaceutical Sciences, School of Pharmacy and Nutrition
University of Navarra
31080 Pamplona, Spain

[g] Departamento de Química Analítica, Química Física e Ingeniería Química and
Instituto de Investigación Química "Andrés del Rio"
Universidad de Alcalá
Alcalá de Henares, 28805 Madrid, Spain

TABLE OF CONTENTS

1. GENERAL METHODS 3

2. DETAILED SYNTHETIC PROCEDURES 4

3. NMR AND MASS SPECTRA 13

4. UV-VIS SPECTROSCOPIC ABSORPTION MEASUREMENTS 25

5. FORMULATIONS OF PDNA/AZO-IAJD NANOCOMPLEXES 37

6. CHARACTERIZATION OF NANOPARTICLES 38

7. EFFECT OF ALTERNATING IRRADIATION IN NANOCOMPLEX TOPOLOGY 38

8. ELECTROPHORESIS MOBILITY SHIFT ASSAY (EMSA) GELS 40

9. TRANSMISSION ELECTRON MICROSCOPY (TEM) 42

10. CELL VIABILITY ASSAY 43

11. *IN VITRO* GENE TRANSFECTION ASSAYS 45

12. *IN VIVO* GENE TRANSFECTION ASSAYS 48

13. STATISTICAL ANALYSIS 49

14. MOLECULAR MECHANICS (MM) AND MOLECULAR DYNAMICS (MD) 49

1. GENERAL METHODS

^1H (^{13}C) NMR experiments were performed at 300 (75.5), 400 (100.6) or 500 (125.7) MHz. 2-D COSY and HSQC experiments were carried out to assist in signal assignment. Solutions were used in $CDCl_3$, CD_3OD, $(CD_3)_2SO$ and D_2O. For ESI mass spectra, 0.1 μM sample concentrations were used, the mobile phase consisting of 50% aq MeOH at 0.1 mL·min^{-1}. Thin-layer chromatography was performed on precoated TLC plates, silica gel 30F-245, with visualization by UV light (254 nm) and by charring with 10% H_2SO_4, 0.2% w/v $Ce(SO_4)_2$ and 5% $(NH_4)_6Mo_7O_{24}\cdot 4\cdot H_2O$ in 2 M H_2SO_4, or 0.1% ninhydrin in EtOH. Column chromatography was performed on silica gel 60 (E. Merck, 230-400 mesh; 50 g silica / g crude material; all compounds were wet loaded using the less polar eluent mixture). Elemental analyses were performed at the Servicio de Microanálisis del Instituto de Investigaciones Químicas de Sevilla, Spain, using an elemental analyser Leco TruSpec CHNS-932 from vacuum-dried samples. The analytical results for C, H, N and S were within ±0.4 of the theoretical values.

2. DETAILED SYNTHETIC PROCEDURES

Starting materials.

The following starting materials were synthesized as previously described: pentaerythritol triallyl ether (**11**),[1] (*E*)-4,4'-di(propargyloxy)azobenzene (**16**),[2] 2-(*N*-*tert*-butoxycarbonylamino)ethyl isothiocyanate **19**,[3] 2-dimethylaminoethyl isothiocyanate **20**[4] and 2-[bis[2-(*tert*-butoxycarbonylamino)ethyl]amino]ethyl isothiocyanate **21**.[5]

Synthesis of the ionizable dendron precursor 8.

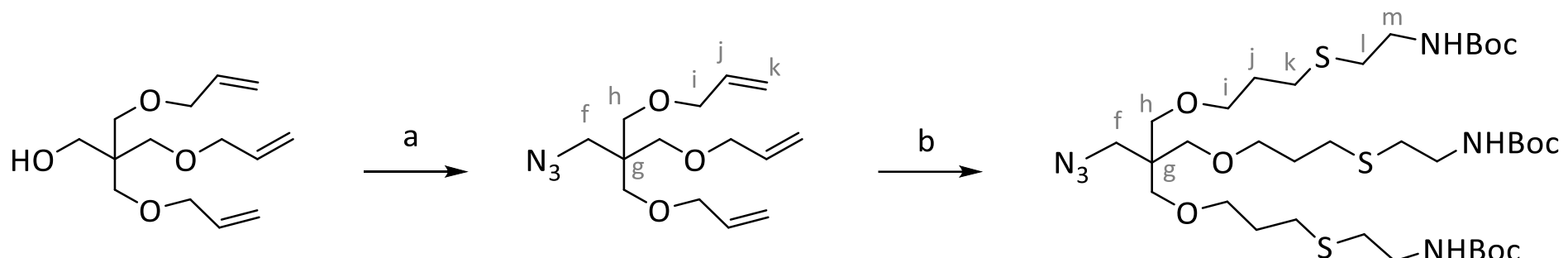

6 7 8

Scheme S1. Reactions and conditions: a) i. Tf_2O, pyridine, –25 ºC, 2 h; ii. NaN_3, DMF, rt, 18 h, 81%; b) *N*-*tert*-butoxycarbonyl-2-aminoethanethiol, DMPA, λ_{irr} = 365 nm, toluene, –50 ºC, 30 min, 85%.

2,2,2-Tris(allyloxymethyl)-1-azidoethane (7).

To a solution of pentaerythritol triallyl ether **6** (5.20 g, 20.3 mmol) in dry CH_2Cl_2 (100 mL), pyridine (24.5 mL, 0.31 mol, 15 equiv) was added under a nitrogen atmosphere. The mixture was cooled to –25 °C, and Tf_2O (2.12 mL, 31 mmol, 1.5 equiv) was added dropwise. After stirring at this temperature for 2.5 h, the reaction mixture was diluted with CH_2Cl_2 (100 mL) and successively washed with H_2O (2 × 50 mL), 2 N H_2SO_4 (2 × 50 mL), saturated aqueous $NaHCO_3$ (2 × 50 mL), and brine (50 mL). The organic layer was dried over $MgSO_4$, filtered, and concentrated. The resulting crude material was dissolved in dry DMF (100 mL), and NaN_3 (6.60 g, 101.5 mmol, 5.0 equiv) was added under nitrogen. The reaction was stirred at room temperature for 18 h, then diluted with a 1:1 toluene–Et_2O mixture (100 mL) and washed with H_2O (2 × 50 mL), saturated aqueous $NaHCO_3$ (2 × 50 mL), and brine (50 mL). The organic layer was dried over $MgSO_4$, filtered, and concentrated to afford **7** as a colourless oil. Yield: 4.6 g (81%). R_f 0.63 (1:1 cyclohexane-EtOAc). ^{1}H NMR (300 MHz, $CDCl_3$): δ 5.85-5.76 (m, 3 H, CH_2-j), 5.18 (ddd, 3 H, $^3J_{H,H}$ = 13.0 Hz, $^2J_{H,H}$ = 2.3 Hz, $^4J_{H,H}$ = 1.3 Hz, CH_2-k_{trans}), 5.08 (ddd, 3 H, $^3J_{H,H}$ = 7.8 Hz, $^2J_{H,H}$ = 2.3 Hz, $^4J_{H,H}$ = 1.0 Hz, CH_2-k_{cis}), 3.88 (dt, 6 H, $^3J_{H,H}$ = 4.1 Hz, $^4J_{H,H}$ = 1.3 Hz, CH_2-i), 3.33 (s, 8 H, CH_2-f, CH_2-h). ^{13}C NMR (75.5 MHz, $CDCl_3$): δ 134.9 (C-j), 116.5 (C-k), 72.3 (C-i), 69.1 (C-h), 52.0 (C-f), 45.4 (C-g). ESIMS: *m/z* 254.22 $[M – N_2 + H]^+$, 535.58 $[2M – N_2 + H]^+$, 788.72 $[3M – N_2 + H]^+$. Anal. Calcd for $C_{14}H_{23}N_3O_3$: C, 59.77; H, 8.24; N, 14.94. Found: C, 59.86; H, 8.34; N, 14.78.

2,2,2-Tris[[3-[2-(*tert*-butoxycarbonylamino)ethyl]thio]propoxymethyl]-1-azidoethane (8). To a solution of the allyl derivative **7** (4.63 g, 16.4 mmol) in anhydrous toluene (100 mL), commercial *N*-*tert*-butoxycarbonyl-2-aminoethanethiol (10.1 mL, 58.9 mmol, 3.6 eq) and DMPA (164 mg, 0.7 mmol, 0.04 eq) were added under a nitrogen atmosphere. The mixture was irradiated with UV light (λ = 365 nm) for 30 min at –50 ºC. The solvent was removed under reduced pressure and the crude was purified by column chromatography (6:1 → 4:1 cyclohexane-EtOAc), affording **8** as a colourless oil. Yield: 11.3 g (85%). R_f 0.65 (1:1 cyclohexane-EtOAc). ^{1}H NMR (300 MHz, $CDCl_3$): δ 4.95 (bs, 3 H, NH), 3.40 (t, 6 H, $^3J_{H,H}$ = 6.0 Hz, CH_2-i), 3.25-3.21 (m, 14 H, CH_2-h, CH_2-m, CH_2-f), 2.57 (t, 6 H, $^3J_{H,H}$ = 6.6 Hz, CH_2-l), 2.52 (t, 6 H, $^3J_{H,H}$ = 7.4 Hz, CH_2-k), 1.75-1.71 (m, 6 H, CH_2-j), 1.38 (s, 9 H, CMe_3). ^{13}C NMR (75.5 MHz, $CDCl_3$): δ 155.8 (CO), 79.3 (*C*Me_3), 69.6 (C-i), 69.5 (C-h), 51.9 (C-f), 39.7 (C-m), 32.2 (C-l), 31.1 (C-g), 29.7 (C-j), 28.5 (C*Me_3*), 28.4 (C-k). ESIMS: *m/z* 713.49 [M – Boc + H]$^+$, 857.75 [M + HCO_2]$^+$. Anal. Calcd for $C_{34}H_{68}N_6O_9S_3$: C, 51.70; H, 8.43; N, 10.34; S, 11.83. Found: C, 51.82; H, 8.71; N, 10.29; S, 11.26.

Synthesis of the hydrophobic dendron precursor 10.

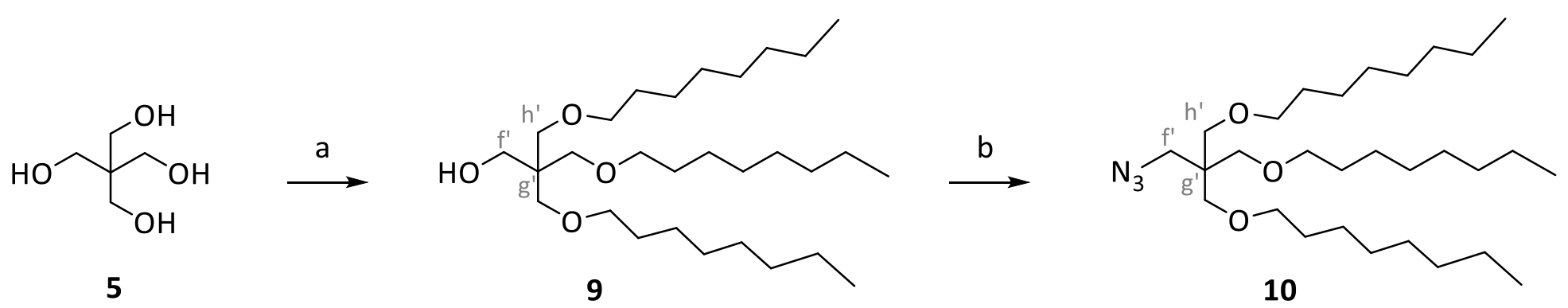

5 9 10

Scheme S2. Reactions and conditions: a) DMF, octyl bromide, KOH, rt, 2 h, 63%; b) i. Tf_2O, pyridine, -25 ºC, 2 h; ii. NaN_3, DMF, rt, 18 h, 96%.

2,2,2-Tris(octyloxymethyl)-1-ethanol (9). To a solution of commercial pentaerythritol **5** (8.0 g, 58.8 mmol) in anhydrous DMF (300 mL), octyl bromide (32 mL, 235 mmol, 4.0 equiv) was added, and the mixture was stirred at room temperature. Finely ground KOH (13.2 g, 235 mmol, 4.0 equiv), previously dried under vacuum, was then added in portions over 30 min, and the reaction was stirred for an additional 2 h at room temperature. The solvent was removed under reduced pressure, and the residue was taken up in CH_2Cl_2 (100 mL) and washed with H_2O (2 × 70 mL), saturated aqueous $NaHCO_3$ (2 × 70 mL), and brine (50 mL). The organic layer was dried over $MgSO_4$, filtered, and concentrated to dryness under reduced pressure. The resulting residue was purified by column chromatography (cyclohexane → 20:1 cyclohexane-EtOAc) to afford **9** as a colourless oil. Yield: 17.6 g (63%). R_f 0.83 (20:1 cyclohexane-EtOAc). ^{1}H NMR (300 MHz, $CDCl_3$): δ 3.37-3.29 (m, 14 H, CH_2-f, CH_2-h, $OCH_{2\text{-octyl}}$), 1.51-1.42 (m, 6 H, $CH_{2\text{-octyl}}$), 1.26-1.21 (m, 30 H, $CH_{2\text{-octyl}}$), 0.81 (t, 9 H, $^3J_{H,H}$ = 6.5 Hz, $CH_{3\text{-octyl}}$). ^{13}C NMR (75.5 MHz, $CDCl_3$): δ 71.8 (C-h), 71.5 (CH_2), 66.7 (C-f), 44.7 (C-g), 31.9 (CH_2), 29.7 (CH_2), 29.7 (CH_2), 29.5 (CH_2), 29.3

(CH_2), 26.2 (CH_2), 22.7 (CH_2), 14.1 (CH_3). ESIMS: *m/z* 473.53 $[M + H]^+$. Anal. Calcd for $C_{29}H_{60}O_4$: C, 73.67; H, 12.79. Found: C; 73.79; H, 12.90.

2,2,2-Tris(octyloxymethyl)-1-azidoethane (10). To a solution of **9** (17.6 g, 37.4 mmol) in dry CH_2Cl_2 (250 mL) under a nitrogen atmosphere, anhydrous pyridine (45 mL, 561 mmol, 15 equiv) was added. The mixture was cooled to –25 °C, and Tf_2O (5.8 mL, 56.1 mmol, 1.5 equiv) was added dropwise. The reaction was stirred at this temperature for 2.5 h and then washed successively with H_2O (2 × 70 mL), 2 N H_2SO_4 (2 × 70 mL), saturated aqueous $NaHCO_3$ (2 × 70 mL), and brine (50 mL). The organic layer was dried over $MgSO_4$, filtered, and concentrated. The resulting crude product was dissolved in anhydrous DMF (250 mL), and NaN_3 (12 g, 187 mmol, 5.0 equiv) was added. The mixture was stirred at room temperature for 18 h, after which the solvent was removed under reduced pressure. The residue was taken up in CH_2Cl_2 (250 mL) and washed with H_2O (2 × 100 mL), saturated aqueous $NaHCO_3$ (2 × 100 mL), and brine (50 mL). The organic layer was dried over $MgSO_4$, filtered, and concentrated. Purification by column chromatography (cyclohexane → 20:1 cyclohexane–EtOAc) afforded **15** as a colourless oil. Yield: 17.9 g (96%). R_f 0.87 (20:1 cyclohexane-EtOAc). ^{1}H NMR (300 MHz, $CDCl_3$): 3.32-3.25 (m, 14 H, CH_2-f, CH_2-h, $OCH_{2\text{-octyl}}$), 1.48-1.42 (m, 6 H, $CH_{2\text{-octyl}}$), 1.26-1.21 (m, 30 H, $CH_{2\text{-octyl}}$), 0.81 (t, 9 H, $^3J_{H,H}$ = 6.5 Hz, $CH_{3\text{-octyl}}$). ^{13}C NMR (75.5 MHz, $CDCl_3$): δ 71.5 (C-h), 69.5 (CH_2), 52.2 (C-f), 45.5 (C-g), 31.9 (CH_2), 29.6 (CH_2), 29.5 (CH_2), 29.3 (CH_2), 26.2 (CH_2), 22.7 (CH_2), 14.1 (CH_3). ESIMS: *m/z* 497.43 $[M + H]^+$. Anal. Calcd for $C_{29}H_{59}N_3O_3$: C, 69.97; H, 11.95; N, 8.44. Found: C, 70.24; H, 12.17; N, 8.30.

Synthesis of Azo-IAJD 1.

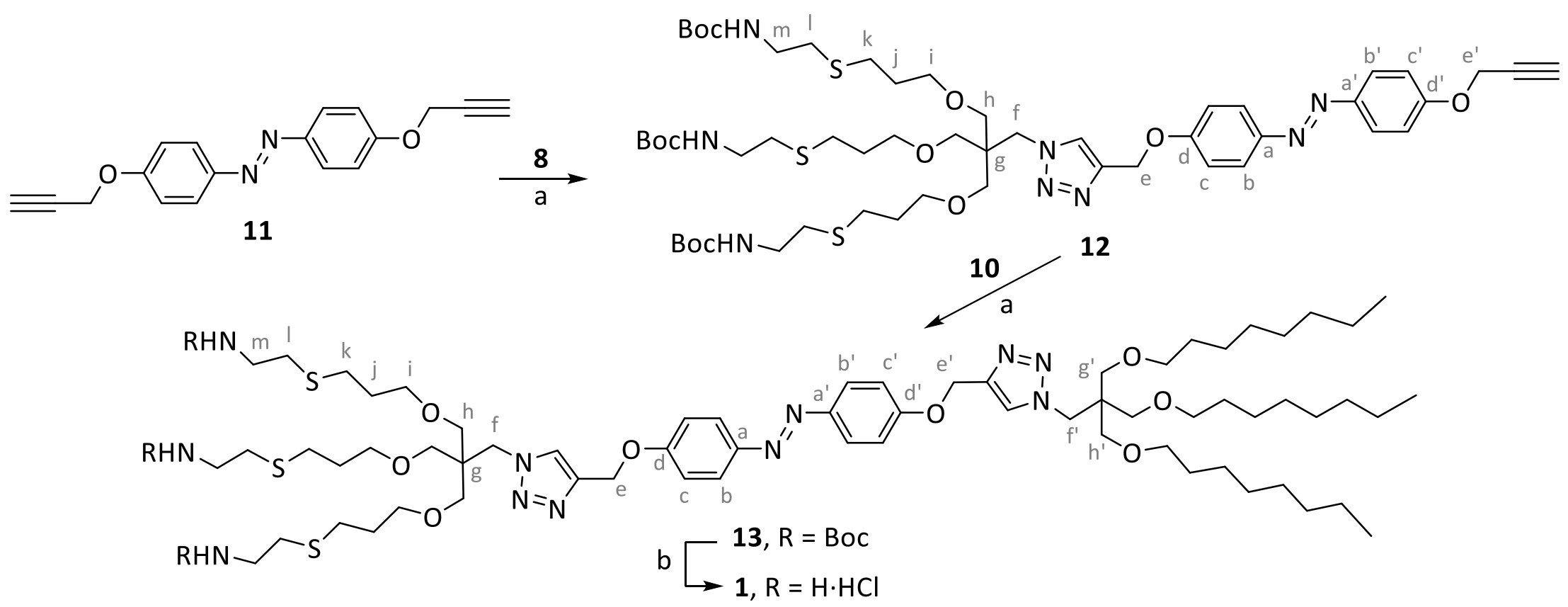

Scheme S3. Reactions and conditions: a) $CuSO_4\cdot 5H_2O$, sodium ascorbate, DMF, H_2O, rt, 30 h, 24-73%; b) 1. 1:1 CH_2Cl_2-TFA, rt, 10 min; 2. 0.1 M HCl, 5 min, quant.

4-{1-{2,2,2-Tris{3-[2-(*N-tert*-butoxycarbonylamino)ethylthio]propoxymethyl}ethyl}-1*H*-1,2,3-triazol-4-ylmethoxy}-4’-

propargyloxyazobenzene (12). To a solution of 4,4'-di(propargyloxy)azobenzene **11** (0.847 g, 2.92 mmol) in DMF (84 mL), **8** (1.187 g, 1.46 mmol, 0.5 eq), $CuSO_4 \cdot 5H_2O$ (73 mg, 0.292 mmol, 0.1 eq) in H_2O (1.0 mL) and sodium ascorbate (NaAs, 116 mg, 0.584 mmol, 0.2 eq) in H_2O (1.0 mL) were added. The reaction was stirred for 30 h at rt. The mixture was diluted in 20 mL of toluene-Et_2O (1:1), and washed with 1 M HCl (2 × 20 mL), saturated aq $NaHCO_3$ (2 × 20 mL) and brine (20 mL). The organic layer was dried over $MgSO_4$, filtered, concentrated and purified by column chromatography (4:1 → 2:1 cyclohexane-EtOAc), affording **17** as a yellow oil. Yield: 772 mg (24%). R_f 0.45 (1:1 cyclohexane-EtOAc). ^{1}H NMR (400 MHz, $CDCl_3$): δ 7.91-7.87 (m, 4 H, CH-b, CH-b'), 7.67 (s, 1 H, C=CH), 7.12-7.08 (m, 4 H, CH-c, CH-c'), 5.33 (s, 2 H, CH_2-f), 5.04 (bs, 3 H, $NH_{carbamate}$), 4.78 (d, 2 H, $^3J_{H,H}$ = 3.0 Hz, CH_2-e'), 4.44 (s, 2 H, CH_2-e), 3.45 (t, 6 H, $^3J_{H,H}$ = 7.5 Hz, CH_2-i), 3.32-3.27 (m, 12 H, CH_2-h, CH_2-m), 2.66-2.58 (m, 13 H, CH_2-l, CH_2-k, C≡C*H*), 1.86-1.80 (m, 6 H, CH_2-j), 1.44 (s, 27 H, CMe_3). ^{13}C NMR (100.6 MHz, $CDCl_3$): δ 160.2, 159.5 (C-d, C-d'), 155.8 (CO), 147.5, 147.3 (C-a, C-a'), 143.3 (*C*=CH), 124.8 (C=*C*H), 124.3 (C-b, C-b'), 115.1 (C-c, C-c'), 79.4 (*C*≡CH), 78.1 (C-g), 76.0 (C≡*C*H), 69.6 (C-i), 69.3 (C-h), 62.3 (C-f), 56.0 (C-e'), 50.2 (C-e), 39.8 (C-m), 32.2 (C-l), 29.6 (C-j), 28.4 (C-k, C*Me*$_3$). ESIMS: *m/z* 1104.01 $[M+H]^+$, 1148.39 $[M+HCO_2]^-$. Anal. Calcd for $C_{53}H_{82}N_8O_{11}S_3$: C, 57.69; H, 7.49; N, 10.15; S, 8.72. Found: C, 57.81; H, 7.62; N, 10.04; S, 8.59.

4-{1-{2,2,2-Tris{3-[2-(*N-tert*-butoxycarbonylamino)ethylthio]propoxymethyl}ethyl}-1*H*-1,2,3-triazol-4-ylmethyloxy}-4'-{1-[2,2,2-tris(*n*-octyloxymethyl)ethyl]-1*H*-1,2,3-triazol-4-ylmethyloxy}azobenzene (13). To a solution of **12** (1.277 g, 1.16 mmol) in 1:1 DMF-acetone (40 mL), **10** (635 mg, 1.27 mmol, 1.1 eq), $CuSO_4 \cdot 5H_2O$ (29 mg, 0.116 mmol, 0.1 eq) in H_2O (1.0 mL) and sodium ascorbate (44 mg, 0.232 mmol, 0.2 eq) in H_2O (1.0 mL) were added. The reaction was stirred for 30 h at rt. The mixture was diluted in 20 mL of 1:1 toluene-Et_2O, and washed with 1 M HCl (2 × 20 mL), saturated aq $NaHCO_3$ (2 × 20 mL) and brine (20 mL). The organic layer was dried over $MgSO_4$, filtered, concentrated and purified by column chromatography (3:1 → 2:1 cyclohexane-EtOAc), affording **18** as a yellow oil. Yield: 1.346 g (73%). R_f 0.58 (1:1 cyclohexane-EtOAc). ^{1}H NMR (400 MHz, $CDCl_3$): δ 7.91-7.87 (m, 4 H, CH-b, CH-b'), 7.69, 7.67 (2×s, 2 H, $C{=}CH_{triazole}$), 7.12-7.09 (m, 4 H, CH-c, CH-c'), 5.33, 5.32 (2×s, 4 H, CH_2-f, CH_2-f'), 5.05 (bs, 3 H, $NH_{carbamate}$), 4.46, 4.44 (s, 4 H, CH_2-e, CH_2-e'), 3.46 (t, 6 H, $^3J_{H,H}$ = 6.0 Hz, $OCH_{2\text{-}octyl}$), 3.38-3.28 (m, 22 H, CH_2, CH_2-h, CH_2-h', CH_2-i, CH_2-m), 2.65-2.58 (m, 12 H, CH_2-l, CH_2-k), 1.87-1.81 (m, 6 H, CH_2-j), 1.56-1.53 (m, 6 H, CH_2), 1.45 (s, 27 H, CMe_3), 1.30-1.29 (m, 30 H, $CH_{2\text{-}octyl}$), 0.89 (t, 9 H, $^3J_{H,H}$ = 6.7 Hz, $CH_{3\text{-}octyl}$). ^{13}C NMR (100.6 MHz, $CDCl_3$): δ 160.3, 160.2 (C-d, C-d'), 155.8 (CO), 147.3 (C-a, C-a'), 143.2 (*C*=CH), 124.8 (C=*C*H), 124.4 (C-b, C-b'), 115.1 (C-c, C-c'), 79.4, 76.0 (C-g, C-g'), 71.53 (*C*Me$_3$), 69.6, 69.5, 69.3 (C-h, C-i, $OCH_{2\text{-}octyl}$), 62.4, 62.3 (C-e, C-e'), 50.6, 50.3 (C-f, C-f'), 39.8 (C-m), 32.3 ($CH_{2\text{-}octyl}$), 31.9 (C-l), 29.6 (C-j), 29.5 (C-k), 29.3, 28.6 ($CH_{2\text{-}octyl}$), 28.4

(CMe_3), 26.2, 22.7 ($CH_{2\text{-octyl}}$), 14.1 ($CH_{3\text{-octyl}}$). ESIMS: *m/z* 1602.07 $[M + H]^+$. Anal. Calcd for $C_{82}H_{141}N_{11}O_{14}S_3$: C, 61.51; H, 8.88; N, 9.62; S, 6.01. Found: C, 61.63; H, 9.01; N, 9.47; S, 5.78.

4-{1-{2,2,2-Tris[3-(2-aminoethylthio)propoxymethyl]ethyl}-1*H*-1,2,3-triazol-4-ylmethoxy}-4'-{1-[2,2,2-tris(*n*-octyloxymethyl)ethyl]-1*H*-1,2,3-triazol-4-ylmethoxy}azobenzene (1). To a solution of **13** (764 mg, 0.478 mmol) in CH_2Cl_2 (5 mL), TFA (5 mL) was added. After 10 min, the reaction mixture was concentrated under vacuum. The crude was resuspended in 0.1 M HCl, stirred for 5 min at rt and freeze-dried, affording **6** as a dark orange foam. Yield: 673 mg (quant). R_f 0.26 (60:10:1 CH_2Cl_2-CH_3OH-H_2O). ^{1}H NMR (500 MHz, CD_3OD): δ 7.98 (s, 2 H, C=CH_{triazole}), 7.90-7.87 (m, 4 H, CH-b, CH-b'), 7.20-7.16 (m, 4 H, CH-c, CH-c'), 5.33 (s, 4 H, CH_2-e, CH_2-e'), 4.52, 4.48 (2×s, 4 H, CH_2-f, CH_2-f'), 3.50 (t, 6 H, $^3J_{H,H}$ = 6.0 Hz, $OCH_{2\text{-octyl}}$), 3.37 (t, 6 H, $^3J_{H,H}$ = 6.4 Hz, CH_2-i), 3.33, 3.28 (2×s, 4 H, CH_2-h, CH_2-h'), 3.16 (t, 6 H, $^3J_{H,H}$ = 6.8 Hz, CH_2-m), 2.82 (t, 6 H, $^3J_{H,H}$ = 6.8 Hz, CH_2-l), 2.68 (t, 6 H, $^3J_{H,H}$ = 7.2 Hz, CH_2-k), 1.89-1.83 (m, 6 H, CH_2-j), 1.59-1.52 (m, 6 H, $CH_{2\text{-octyl}}$), 1.31-1.30 (m, 30 H, $CH_{2\text{-octyl}}$), 0.90 (t, 9 H, $^3J_{H,H}$ = 6.7 Hz, $CH_{3\text{-octyl}}$). ^{13}C NMR (125.7 MHz, CD_3OD): δ 160.4 (C-d, C-d'), 147.2, 147.2 (C-a, C-a'), 142.9 (*C*=CH), 125.8 (C=*C*H), 124.0, 123.9 (C-b, C-b'), 115.0 (C-c, C-c'), 71.1 (C-i), 69.3, 69.2, 69.0 ($OCH_{2\text{-octyl}}$, C-h, C-h'), 61.4, 61.2 (C-e, C-e'), 50.4, 50.2 (C-f, C-f'), 38.5 (C-m), 31.7, 29.3, 29.2, 29.1 ($CH_{2\text{-octyl}}$), 29.1 (C-j), 28.4 (C-l), 27.8 (C-k), 26.0, 22.4 ($CH_{2\text{-octyl}}$), 13.1 ($CH_{3\text{-octyl}}$). ESIMS: *m/z* 434.59 $[M + 3 H]^{3+}$, 651.16 $[M + 2 H]^{2+}$, 1301.02 $[M + H]^+$, 1345.99 $[M + HCO_2]^-$. Anal. Calcd for $C_{37}H_{63}N_{10}O_4S_4 \cdot 3$ HCl: C, 57.06; H, 8.58; N, 10.93; S, 6.82. Found: C, 56.87; H, 8.37; N, 10.65; S, 6.60.

Synthesis of Azo-IAJD 7-9.

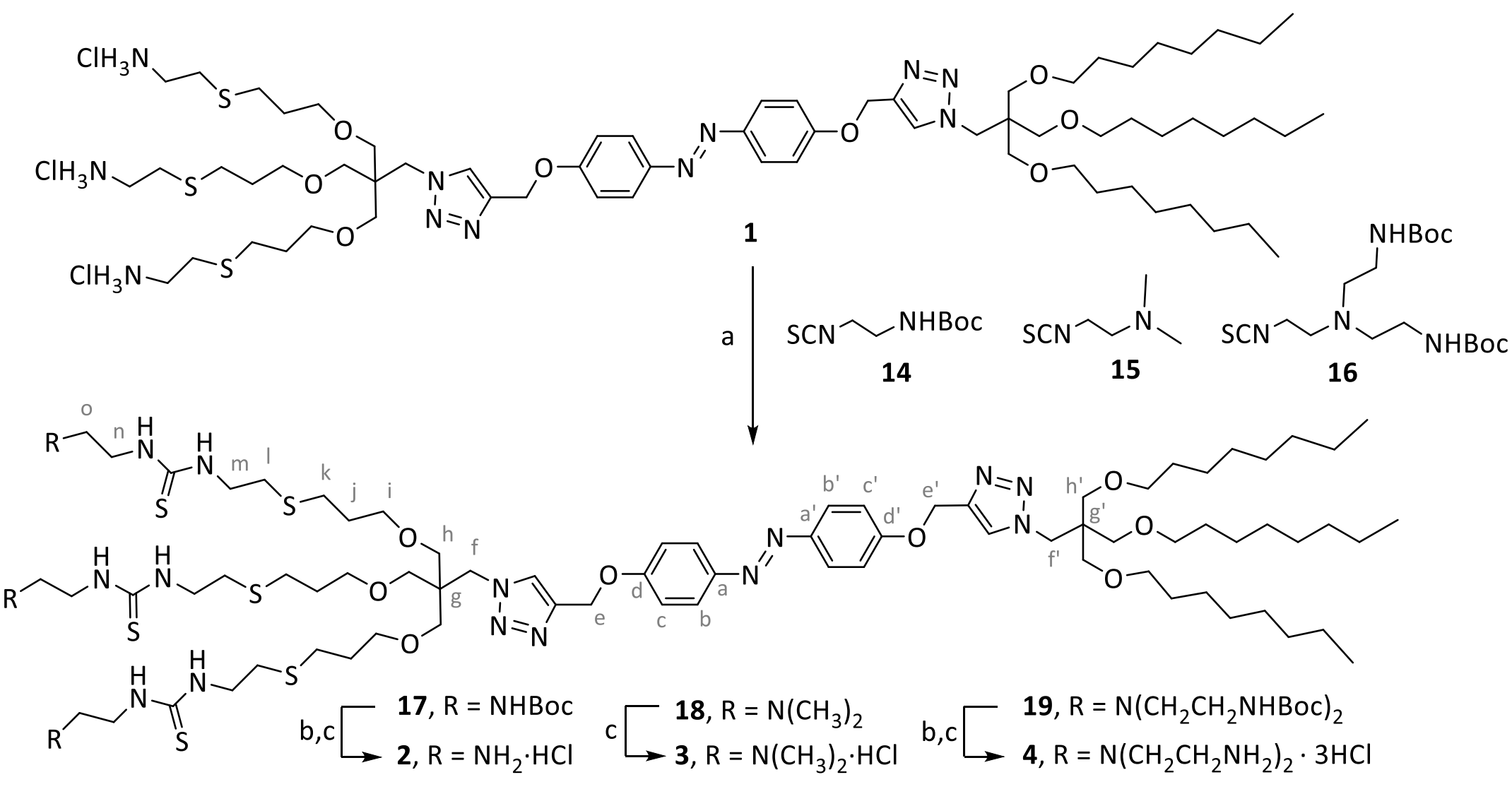

Scheme S4. Reactions and conditions: a) CH_2Cl_2, Et_3N, rt, 18 h, 82-89%; b) 1:1 CH_2Cl_2-TFA, 0 ºC to rt, 2 h; c) 0.1 M HCl, 5 min, freeze-drying, quant.

4-{1-{2,2,2-Tris{3-{2-{*N'*-[2-*N*-(*tert*-butoxycarboyl)aminoethyl]thioureido}ethylthio}propoxymethyl}ethyl}-1*H*-1,2,3-triazol-4-ylmethoxy}-4'-{1-[2,2,2-tris(*n*-octyloxymethyl)ethyl]-1*H*-1,2,3-triazol-4-ylmethoxy}azobenzene (17). To a solution of **1** (176 mg, 0.125 mmol) in CH_2Cl_2 (6 mL) were added Et_3N (52 μL, 0.375 mmol, 3.0 equiv) and 2-(*N*-*tert*-butoxycarbonylamino)ethyl isothiocyanate **14** (84 mg, 0.414 mmol, 3.3 equiv). The reaction mixture was stirred at room temperature for 18 h, then washed with H_2O (2 × 3 mL), saturated aqueous $NaHCO_3$ (2 × 3 mL), and brine (3 mL). The organic layer was dried over $MgSO_4$, filtered, concentrated, and purified by column chromatography (50:1 → 30:1 CH_2Cl_2–CH_3OH) to afford **17** as a yellow oil. Yield: 203 mg (85%). R_f 0.26 (20:1 CH_2Cl_2-CH_3OH). ^{1}H NMR (500 MHz, $CDCl_3$): δ 7.88 (2×d, 4 H, $^3J_{H,H}$ = 9.0 Hz, CH-b, CH-b'), 7.70, 7.66 (2×s, 2 H, C=$CH_{triazole}$), 7.10 (2×d, 4 H, $^3J_{H,H}$ = 9.0 Hz, CH-c, CH-c'), 6.86 (bs, 3 H, $NH_{thiourea}$), 5.33, 5.31 (2×s, 4 H, CH_2-e, CH_2-e'), 5.24 (bs, 3 H, $NH_{carbamate}$), 4.47, 4.45 (2×s, 4 H, CH_2-f, CH_2-f'), 3.71 (bs, 6 H, CH_2-m), 3.61 (bs, 6 H, CH_2-o), 3.47 (t, 6 H, $^3J_{H,H}$ = 6.0 Hz, $OCH_{2\text{-octyl}}$), 3.36 (t, 6 H, $^3J_{H,H}$ = 6.6 Hz, CH_2-i), 3.33-3.29 (m, 18 H, CH_2-h, CH_2-h', CH_2-n), 2.78 (t, 6 H, $^3J_{H,H}$ = 6.7 Hz, CH_2-l), 2.64 (t, 6 H, $^3J_{H,H}$ = 7.2 Hz, CH_2-k), 1.87-1.82 (m, 6 H, CH_2-j), 1.56-1.52 (m, 6 H, $CH_{2\text{-octyl}}$), 1.44 (s, 27 H, CMe_3), 1.35-1.29 (m, 30 H, $CH_{2\text{-octyl}}$), 0.89 (t, 9 H, $^3J_{H,H}$ = 6.9 Hz, $CH_{3\text{-octyl}}$). ^{13}C NMR (125.7 MHz, $CDCl_3$): δ 182.1 (C=S), 160.3, 160.1 (C-d, C-d'), 157.2 (CO), 147.4, 147.2 (C-a, C-a'), 143.2, 143.1 (*C*=CH), 125.2, 124.9 (C=*C*H), 124.4 (C-b, C-b'), 115.1 (C-c, C-c'), 80.0 (*C*Me$_3$), 71.5 (C-i), 69.6, 69.4, 69.2 ($OCH_{2\text{-octyl}}$, C-h, C-h'), 62.3, 62.1 (C-e, C-e'), 50.7, 50.4 (C-f, C-f'), 45.2 (C-n), 43.6 (C-m), 39.9 (C-o), 31.9 (C-l), 31.2, 29.6, 29.4 ($CH_{2\text{-octyl}}$), 29.3 (C-k), 28.5 (C*Me*$_3$), 28.4 (C-k), 28.2, 26.2, 22.7 ($CH_{2\text{-octyl}}$), 14.1 ($CH_{3\text{-octyl}}$). ESIMS: *m/z* 954.96 $[M + 2 H]^{2+}$, 1908.57 $[M + H]^+$, 1906.71 $[M + H]^-$. Anal. Calcd for $C_{91}H_{159}N_{17}O_{14}S_6$: C, 57.29; H, 8.40; N, 12.48; S, 10.08. Found: C, 57.41; H, 8.611; N, 12.20; S, 10.08.

4-{1-{2,2,2-Tris{3-{2-[*N'*-(2-aminoethyl)thioureido]ethylthio}propoxymethyl}ethyl}-1*H*-1,2,3-triazol-4-ylmethoxy}-4'-{1-[2,2,2-tris(*n*-octyloxymethyl)ethyl]-1*H*-1,2,3-triazol-4-ylmethoxy}azobenzene (2). To a solution of **17** (725 mg, 0.138 mmol) in CH_2Cl_2 (5 mL), TFA (5 mL) was added. After 10 min, the reaction mixture was concentrated under vacuum. The crude was resuspended in 0.1 M HCl, stirred for 5 min at rt and freeze-dried, affording **2** as a yellow foam. Yield: 117 mg (quant). R_f 0.60 (60:10:1 CH_2Cl_2-CH_3OH-NH_4OH). ^{1}H NMR (500 MHz, CD_3OD): δ 8.59, 8.54 (2×s, 2 H, C=$CH_{triazole}$), 7.94 (d, 4 H, $^3J_{H,H}$ = 9.0 Hz, CH-b, CH-b'), 7.27 (s, 4 H, CH-c, CH-c'), 5.56, 5.53 (2×s, 4 H, CH_2-e, CH_2-e'), 4.75, 4.71 (2×s, 4 H, CH_2-f, CH_2-f'), 3.87 (t, 6 H, $^3J_{H,H}$ = 6.0 Hz, CH_2-o), 3.70 (t, 6 H, $^3J_{H,H}$ = 6.5 Hz, CH_2-m), 3.52-3.37 (m, 24 H, $OCH_{2\text{-octyl}}$, CH_2-i, CH_2-h, CH_2-h'), 3.20 (t, 6 H, CH_2-n), 2.75 (t, 6 H, CH_2-l), 2.67 (t, 6 H, $^3J_{H,H}$ = 7.2 Hz, CH_2-k), 1.88-1.82 (m, 6 H, CH_2-j), 1.57-1.51 (m, 6 H, $CH_{2\text{-octyl}}$), 1.31-1.29 (m, 30 H, $CH_{2\text{-octyl}}$), 0.89 (t, 9 H, $^3J_{H,H}$ = 6.2 Hz,

$CH_{3\text{-octyl}}$). ^{13}C NMR (125.7 MHz, CD_3OD): δ 183.5 (C=S), 159.7, 159.7 (C-d, C-d'), 147.6, 147.6 (C-a, C-a'), 139.7, 139.8 (C=*C*H), 124.4, 124.3 (C-b, C-b'), 115.2, 115.1 (C-c, C-c'), 71.2, 69.5, 69.3, 69.2 (C-i, $OCH_{2\text{-octyl}}$, C-h, C-h'), 59.5, 59.4 (C-e, C-e'), 53.6 (C-f, C-f'), 45.3 (C-g), 43.7 (C-m), 41.0 (C-o), 39.4 (C-n), 31.7 ($CH_{2\text{-octyl}}$), 30.3 (C-l), 29.5 (C-j), 29.3, 29.2, 29.1 ($CH_{2\text{-octyl}}$), 28.0 (C-k), 26.0, 22.4 ($CH_{2\text{-octyl}}$), 13.1 ($CH_{3\text{-octyl}}$). ESIMS: *m/z* 536.81 $[M + 3 H]^{3+}$, 804.51 $[M + 2 H]^{2+}$, 1609.02 $[M + H]^+$, 1607.14 $[M + H]^-$. Anal. Calcd for $C_{76}H_{135}N_{17}O_8S_6$·3 HCl: C, 53.17; H, 8.10; N, 13.87; S, 11.20. Found: C, 52.97; H, 8.23; N, 13.60; S, 10.99.

4-{1-{2,2,2-Tris{3-{2-{*N'*-(2-(*N*,*N*-diamino)ethyl]thioureido}ethylthio}propoxymethyl}ethyl}-1*H*-1,2,3-triazol-4-ylmethoxy}-4'-{1-[2,2,2-tris(*n*-octyloxymethyl)ethyl]-1*H*-1,2,3-triazol-4-ylmethoxy}azobenzene (3). To a solution of **1** (176 mg, 0.125 mmol) in CH_2Cl_2 (6 mL), Et_3N (52 μL, 0.375 mmol, 3.0 eq) and 2-(*N*,*N*-dimethylamino)ethyl isothiocyanate **15** (13 mg, 0.414 mmol, 3.3 eq) were added. The reaction was stirred 18 h at rt. The solvent was removed under reduced pressure and the crude was purified by column chromatography (150:10:1 → 50:10:1 CH_2Cl_2-CH_3OH-NH_4OH). The resulting solid material was suspended in 0.1 M HCl, stirred for 5 min at rt and freeze-dried, affording **3** as a yellow foam. Yield: 150 mg (89%). R_f 0.61 (70:10:1 CH_2Cl_2-CH_3OH-NH_4OH). ^{1}H NMR (500 MHz, CD_3OD): δ 8.15, 8.09 (2×s, 2 H, $C{=}CH_{triazole}$), 7.91 (d, 4 H, $^3J_{H,H}$ = 8.9 Hz, CH-b, CH-b'), 7.20 (d, 4 H, CH-c, CH-c'), 5.40, 5.38 (2×s, 4 H, CH_2-e, CH_2-e'), 4.57, 4.54 (2×s, 4 H, CH_2-f, CH_2-f'), 3.98 (t, 6 H, $^3J_{H,H}$ = 5.7 Hz, CH_2-n), 3.71 (bs, 6 H, CH_2-m), 3.49 (t, 6 H, $^3J_{H,H}$ = 5.9 Hz, $OCH_{2\text{-octyl}}$), 3.42 (t, 6 H, CH_2-o), 3.37 (t, 6 H, $^3J_{H,H}$ = 6.3 Hz, CH_2-i), 3.34-3.30 (m, 12 H, CH_2-h, CH_2-h'), 2.98 (s, 18 H, NMe_2), 2.75 (t, 6 H, $^3J_{H,H}$ = 7.3 Hz, CH_2-l), 2.68 (t, 6 H, $^3J_{H,H}$ = 7.2 Hz, CH_2-k), 1.88-1.83 (m, 6 H, CH_2-j), 1.57-1.52 (m, 6 H, $CH_{2\text{-octyl}}$), 1.32-1.29 (m, 30 H, $CH_{2\text{-octyl}}$), 0.90 (t, 9 H, $^3J_{H,H}$ = 6.8 Hz, $CH_{3\text{-octyl}}$). ^{13}C NMR (100.6 MHz, D_2O): δ 183.6 (C=S), 160.2, 160.2 (C-d, C-d'), 147.3, 147.3 (C-a, C-a'), 142.0, 141.9 (*C*=CH), 126.7, 126.6 (C=*C*H), 124.2 (C-b, C-b'), 115.2, 115.1 (C-c, C-c'), 71.2 (C-i), 69.5, 69.2, 69.1 ($OCH_{2\text{-octyl}}$, C-h, C-h'), 60.9 (C-e, C-e'), 57.2 (C-m), 51.4, 51.3 (C-f, C-f'), 43.8 (C-o), 42.8 (NCH_3), 38.8 (C-n), 31.7 ($CH_{2\text{-octyl}}$), 30.3 (C-l), 29.6, 29.3, 29.2 ($CH_{2\text{-octyl}}$), 29.0 (C-j), 28.8 ($CH_{2\text{-octyl}}$), 28.1 (C-k), 26.1, 22.4 ($CH_{2\text{-octyl}}$), 13.2 ($CH_{3\text{-octyl}}$). ESIMS: *m/z* 423.93 $[M + 4 H]^{4+}$, 564.87 $[M + 3 H]^{3+}$, 846.58 $[M + 2 H]^{2+}$, 1692.15 $[M + H]^+$, 1690.41 $[M + H]^-$. Anal. Calcd for $C_{82}H_{147}N_{17}O_8S_6$·3 HCl: C, 54.69; H, 8.40; N, 13.22; S, 10.68. Found: C, 54.47; H, 8.32; N, 12.97; S, 10.56.

4-{1-{2,2,2-Tris{3-{2-{*N'*-{2-{*N*-bis{2-*N*-(*tert*-butoxycarbonyl)amino}ethyl}amino}ethyl}thioureido}ethylthio}propoxymethyl}ethyl}-1*H*-1,2,3-triazol-4-ylmethyloxy}-4'-{1-[2,2,2-tris(*n*-octyloxymethyl)ethyl]-1*H*-1,2,3-triazol-4-ylmetoxy}azobenzene (19). To a solution of **1** (176 mg, 0.125 mmol) in CH_2Cl_2 (6 mL), Et_3N (52 μL, 0.375 mmol, 3.0 eq) and 2-{*N*-{bis[2-(*N*-*tert*-butoxycarbonyl)amino]ethyl}amino}ethyl isothiocyanate **16** (161 mg, 0.414 mmol, 3.3 eq) were added. The reaction was stirred 18 h at rt.

The mixture was washed with H_2O (2 × 3 mL), saturated aq $NaHCO_3$ (2 × 3 mL) and brine (3 mL). The organic layer was dried over $MgSO_4$, filtered, concentrated and purified by column chromatography (50:1 → 15:1 CH_2Cl_2-CH_3OH), obtaining **19** as a yellow oil. Yield: 340 mg (82%). R_f 0.60 (9:1 CH_2Cl_2-CH_3OH). ^{1}H NMR (400 MHz, $CDCl_3$): δ 8.00 (bs, 3 H, $NH_{thiourea}$), 7.86 (2×d, 4 H, $^3J_{H,H}$ = 8.8 Hz, CH-b, CH-b'), 7.71, 7.65 (2×s, 2 H, $C{=}CH_{triazole}$), 7.54 (bs, 3 H, $NH_{thiourea}$), 7.10-7.08 (m, 4 H, CH-c, CH-c'), 5.74 (bs, 6 H, $NH_{carbamate}$), 5.30, 5.29 (2×s, 4 H, CH_2-e, CH_2-e'), 4.43 (s, 4 H, CH_2-f, CH_2-f'), 3.89 (bs, 6 H, CH_2-o), 3.73 (bs, 6 H, CH_2-m), 3.45-3.44 (m, 24 H, $OCH_{2\text{-}octyl}$, CH_2-q, CH_2-n), 3.34 (t, 6 H, $^3J_{H,H}$ = 6.5 Hz, CH_2-i), 3.26 (s, 12 H, CH_2-h, CH_2-h'), 3.18 (bs, 12 H, CH_2-p), 2.73 (t, 6 H, $^3J_{H,H}$ = 6.5 Hz, CH_2-l), 2.63 (t, 6 H, $^3J_{H,H}$ = 7.0 Hz, CH_2-k), 1.85-1.81 (m, 6 H, CH_2-j), 1.58-1.49 (m, 6 H, $CH_{2\text{-}octyl}$), 1.42 (s, 54 H, CMe_3), 1.32-1.26 (m, 30 H, $CH_{2\text{-}octyl}$), 0.86 (t, 9 H, $^3J_{H,H}$ = 6.7 Hz, $CH_{3\text{-}octyl}$). ^{13}C NMR (100.6 MHz, $CDCl_3$): δ 183.2 (C=S), 160.3, 160.2 (C-d, C-d'), 156.5 (CO), 147.3, 147.3 (C-a, C-a'), 143.1, 143.1 (*C*=CH), 125.2, 124.8, 124.4 (C=*C*H, C-b, C-b'), 115.1, 115.0 (C-c, C-c'), 80.2 (*C*Me_3), 71.5 (C-i), 69.7, 69.4, 69.3 ($OCH_{2\text{-}octyl}$, C-h, C-h'), 62.4, 62.2 (C-e, C-e'), 55.0 (C-p), 50.6, 50.4 (C-f, C-f'), 45.2 (C-m), 43.9 (C-o), 39.9 (C-n), 36.7 (C-q), 31.8 ($CH_{2\text{-}octyl}$), 31.1 (C-l), 29.6 ($CH_{2\text{-}octyl}$), 29.4 (C-k), 28.6 (C-j), 28.4 (C*Me*$_3$), 26.2, 22.6 ($CH_{2\text{-}octyl}$), 14.1 ($CH_{3\text{-}octyl}$). ESIMS: *m/z* 617.77 $[M + 4 H]^{4+}$, 823.29 $[M + 3 H]^{3+}$, 1234.21 $[M + 2 H]^{2+}$, 1278.36 $[M + 2 HCO_2]^{2-}$, 1255.81 $[M + HCO_2 + H]^{2-}$. Anal. Calcd for $C_{118}H_{213}N_{23}O_{20}S_6$: C, 57.46; H, 8.70; N, 13.06; S, 7.80. Found: C, 57.26; H, 8.573; N, 12.87; S, 7.49.

4-{1-{2,2,2-Tris{3-{2-{*N'*-{2-[*N*-bis(2-aminoethyl)amino]ethyl}thioureido}ethylthio}propoxymethyl}ethyl}-1*H*-1,2,3-triazol-4-ylmethoxy}-4'-{1-[2,2,2-tris(*n*-octyloxymethyl)ethyl]-1*H*-1,2,3-triazol-4-ylmethoxy}azobenzene (4). To a solution of **19** (340 mg, 0.138 mmol) in CH_2Cl_2 (5 mL), TFA (5 mL) was added. After 10 min, the reaction mixture was concentrated under vacuum. The crude was resuspended in 0.1 M HCl, stirred for 5 min at rt and freeze-dried, affording **4** as a yellow foam. Yield: 168 mg (quant). R_f 0.33 (60:10:1 CH_2Cl_2-CH_3OH-NH_4OH). ^{1}H NMR (500 MHz, 323K, $(CD_3)_2SO$): δ 8.38 (bs, 3 H, $NH_{thiourea}$), 8.01, 7.92 (2×s, 1 H, $C{=}CH_{triazole}$), 7.84-7.82 (m, 4 H, CH-b, CH-b'), 7.23-7.19 (m, 4 H, CH-c, CH-c'), 5.28 (s, 4H, CH_2-e, CH_2-e'), 4.40-4.35 (m, 4 H, CH_2-f, CH_2-f'), 4.16-3.73 (m, 9 H, CH_2-o, $NH_{thiourea}$), 3.46-2.77 (m, 60 H, CH_2-p, CH_2-q, CH_2-n, $OCH_{2\text{-}octyl}$, CH_2-i, CH_2-h, CH_2-h', CH_2-m), 2.67-2.58 (m, 12 H, CH_2-l, CH_2-k), 1.78-1.76 (m, 6 H, CH_2-j), 1.48-1.43 (m, 6 H, CH_2), 1.29-1.23 (m, 30 H, $CH_{2\text{-}octyl}$), 0.84 (t, 9 H, $^3J_{H,H}$ = 6.7 Hz, $CH_{3\text{-}octyl}$). ^{13}C NMR (125.7 MHz, $(CD_3)_2SO$): δ 160.7 (C-d, C-d'), 146.8 (C-a, C-a'), 142.4 (C=*C*H), 126.5, 124.6 (C-b, C-b'), 115.9, 115.8 (C-c, C-c'), 71.1, 69.6, 69.3 (C-i, $OCH_{2\text{-}octyl}$, C-h, C-h'), 61.9, 61.8 (C-e, C-e'), 51.0 (C-q, C-p), 50.4 (C-f, C-f'), 45.1 (C-o), 36.4 (C-n, C-m), 31.7, 29.4 ($CH_{2\text{-}octyl}$), 29.3 (C-l, C-j), 29.2 (C-k), 26.1, 22.5 ($CH_{2\text{-}octyl}$), 14.4 ($CH_{3\text{-}octyl}$). ESIMS: *m/z* 374.25 $[M + 5 H]^{5+}$, 467.51 $[M + 4 H]^{4+}$, 622.96 $[M + 3 H]^{3+}$, 933.67 $[M + 2 H]^{2+}$, 1866.32

[M + H]$^+$, 1864.75 [M + H]$^-$. Anal. Calcd for $C_{88}H_{165}N_{23}O_8S_6 \cdot 9\ HCl$: C, 48.18; H, 7.99; N, 14.68; S, 8.78. Found: C, 47.89; H, 7.761; N, 14.37; S, 8.53.

3. NMR AND MASS SPECTRA

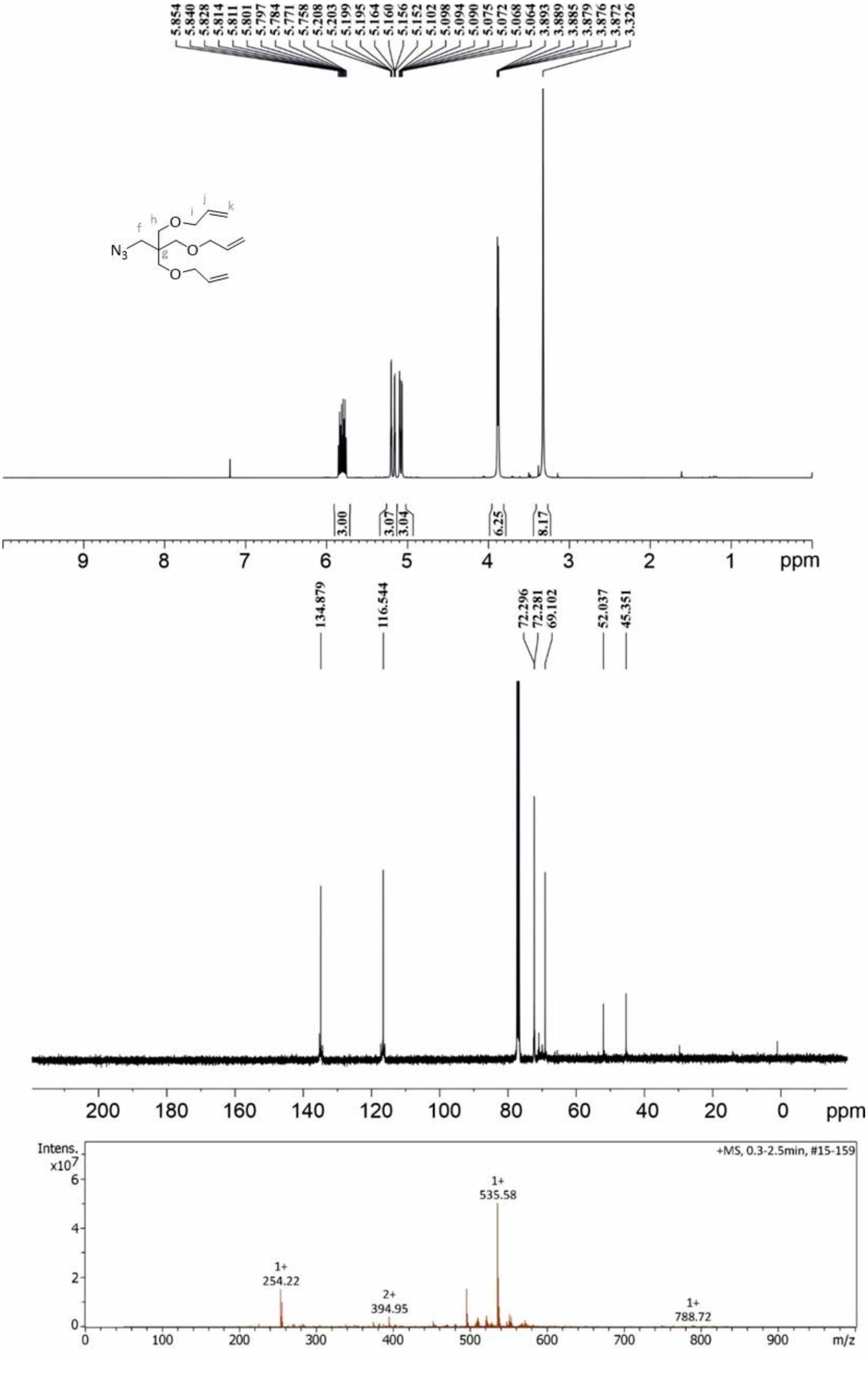

Figure S1. ^{1}H, ^{13}C NMR (300 MHz, 75.5 MHz, $CDCl_3$) and ESIMS spectra of **7**.

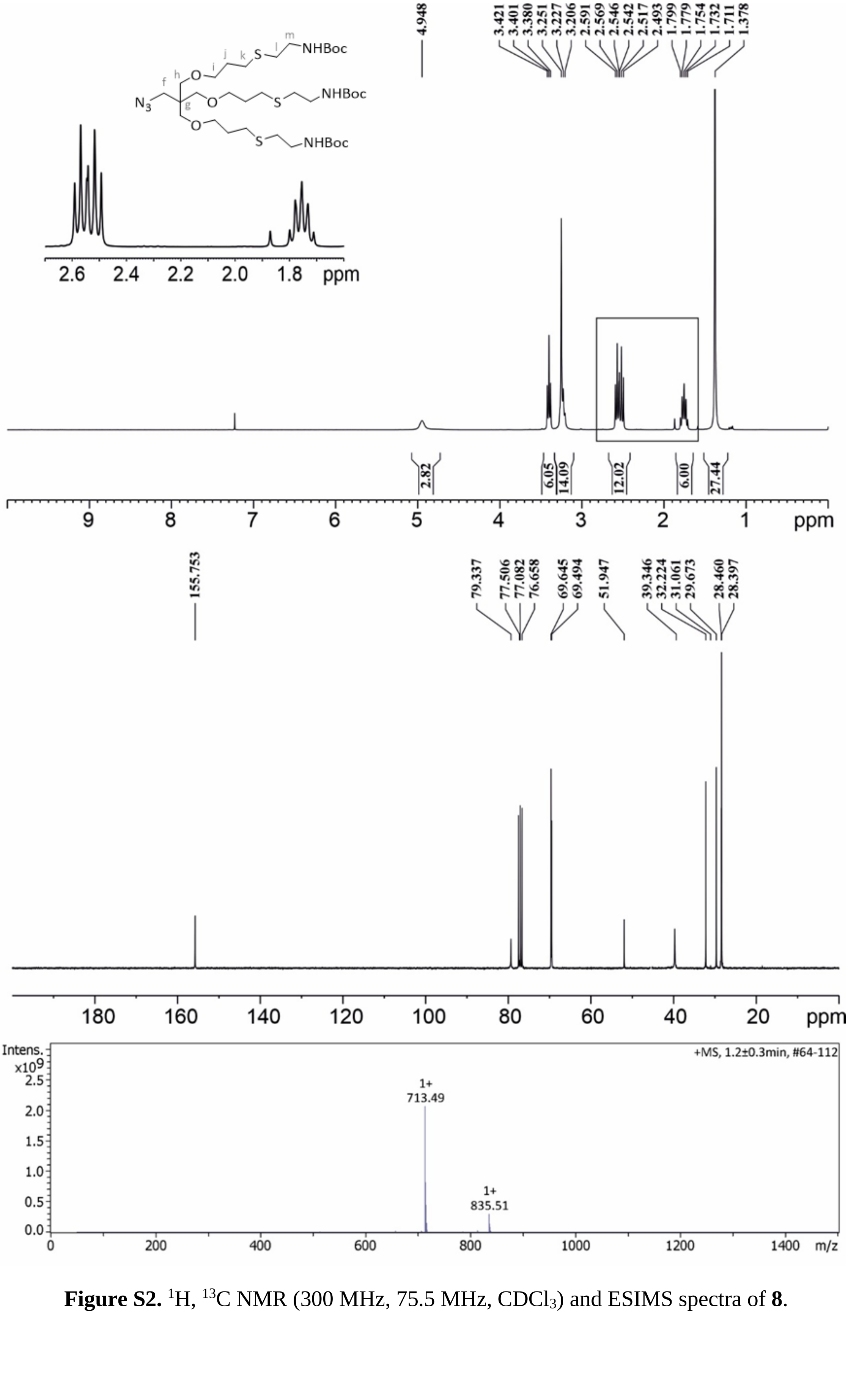

Figure S2. ^{1}H, ^{13}C NMR (300 MHz, 75.5 MHz, $CDCl_3$) and ESIMS spectra of **8**.

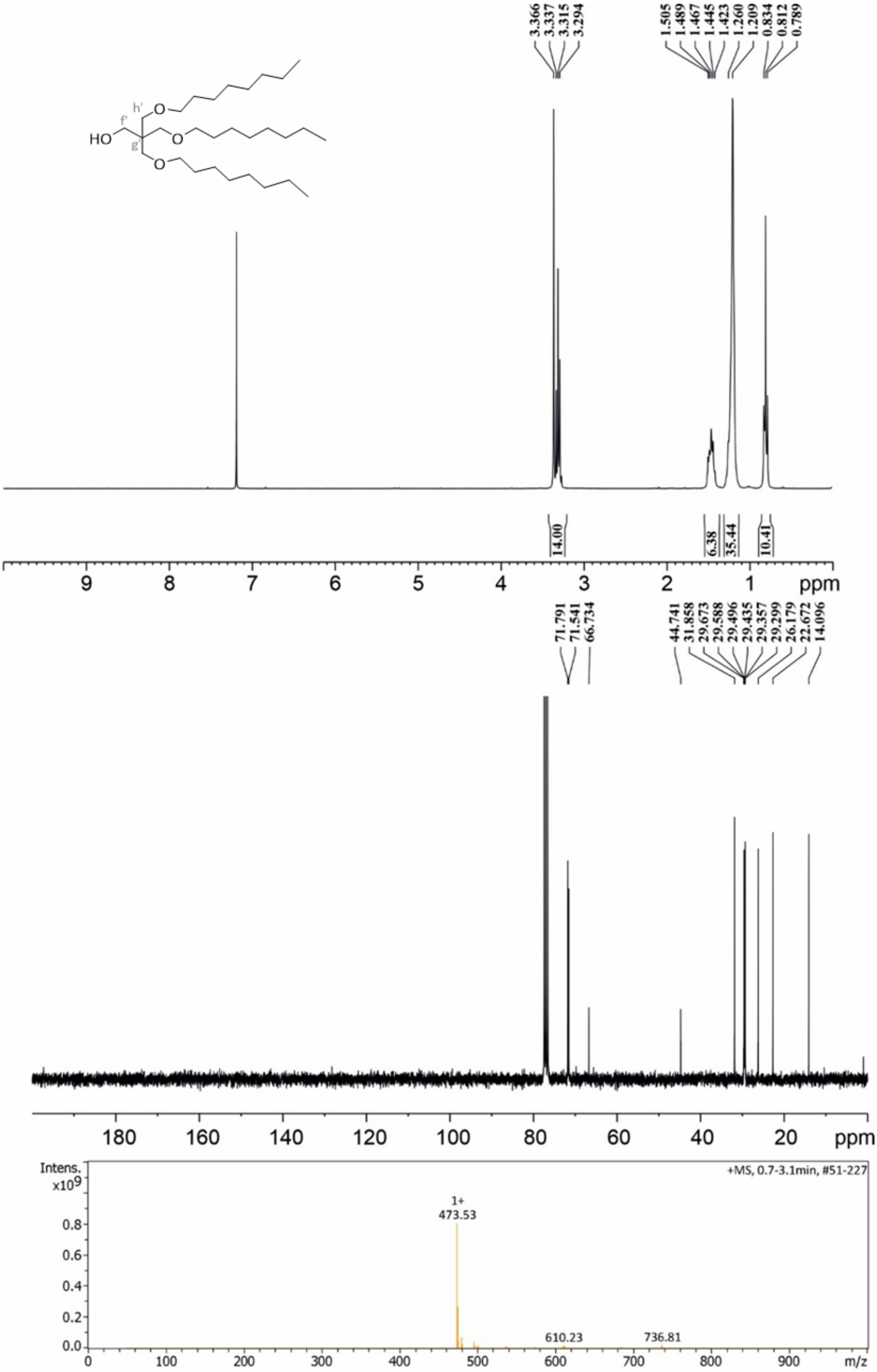

Figure S3. ^{1}H and ^{13}C NMR spectra (300 MHz, 75.5 MHz, $CDCl_3$) and ESIMS spectra of **9**.

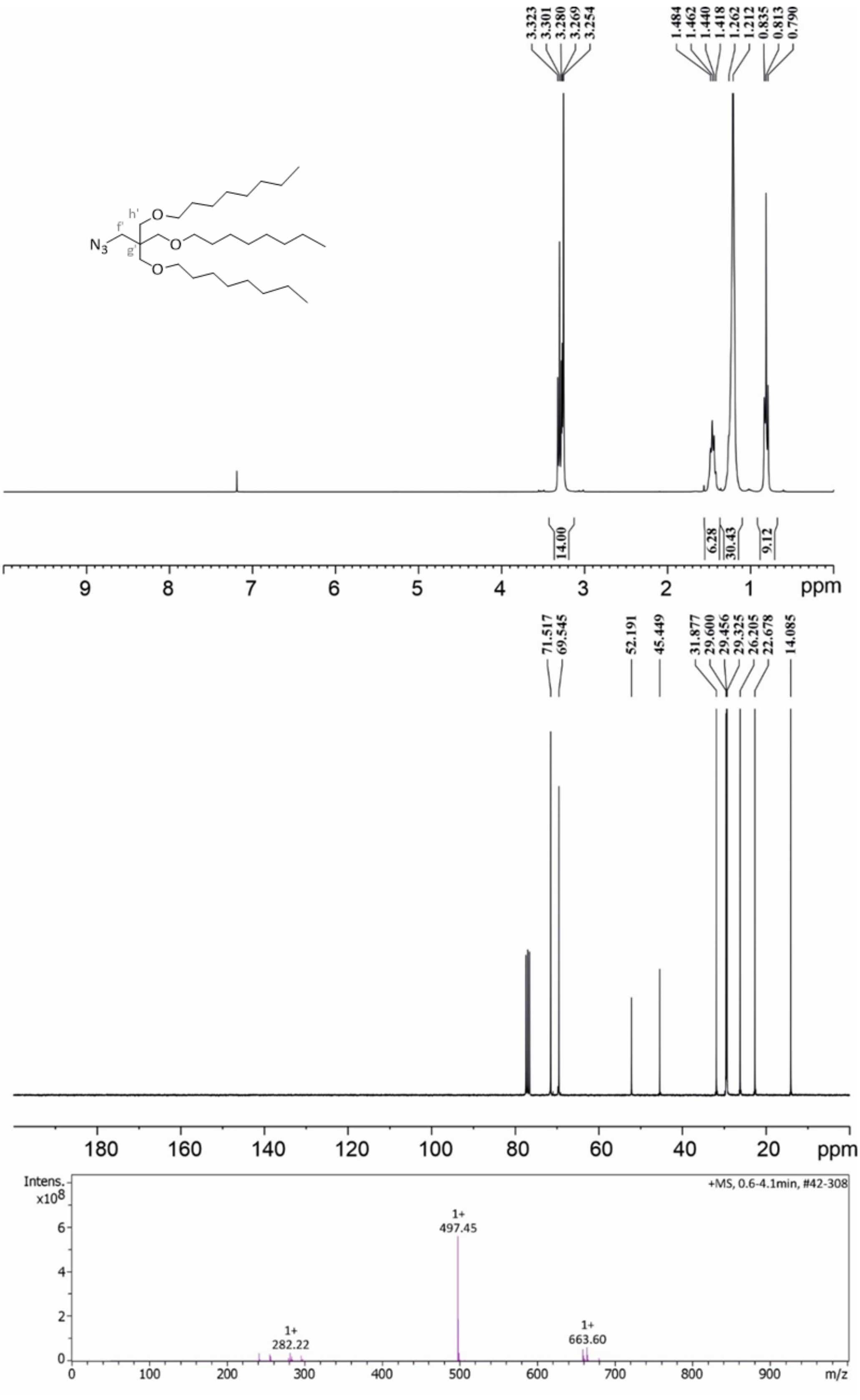

Figure S4. ^{1}H and ^{13}C NMR spectra (300 MHz, 75.5 MHz, $CDCl_3$) and ESIMS spectra of **10**.

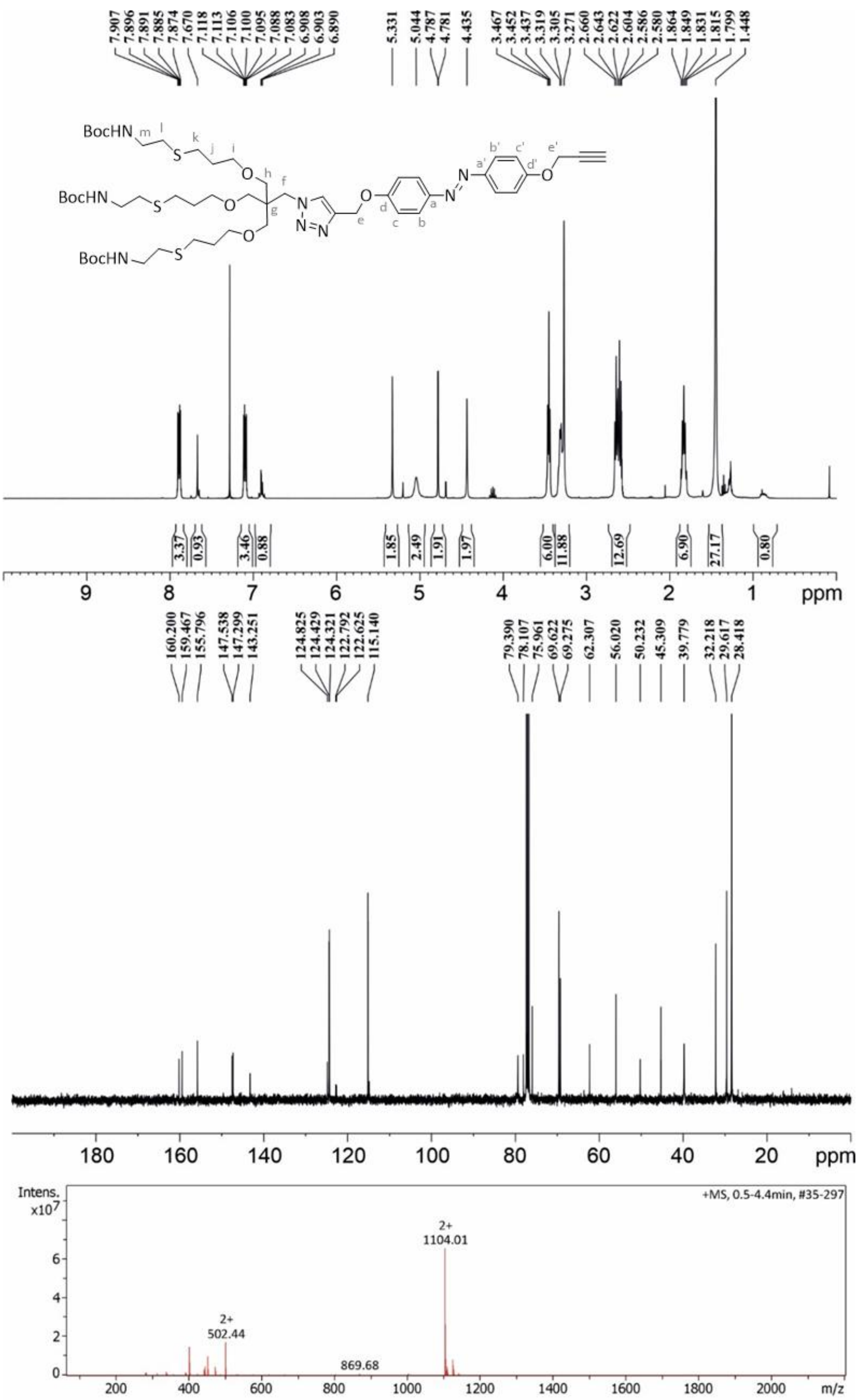

Figure S5. ^{1}H and ^{13}C NMR spectra (400 MHz, 100.6 MHz, $CDCl_3$) and ESIMS spectra of **12**.

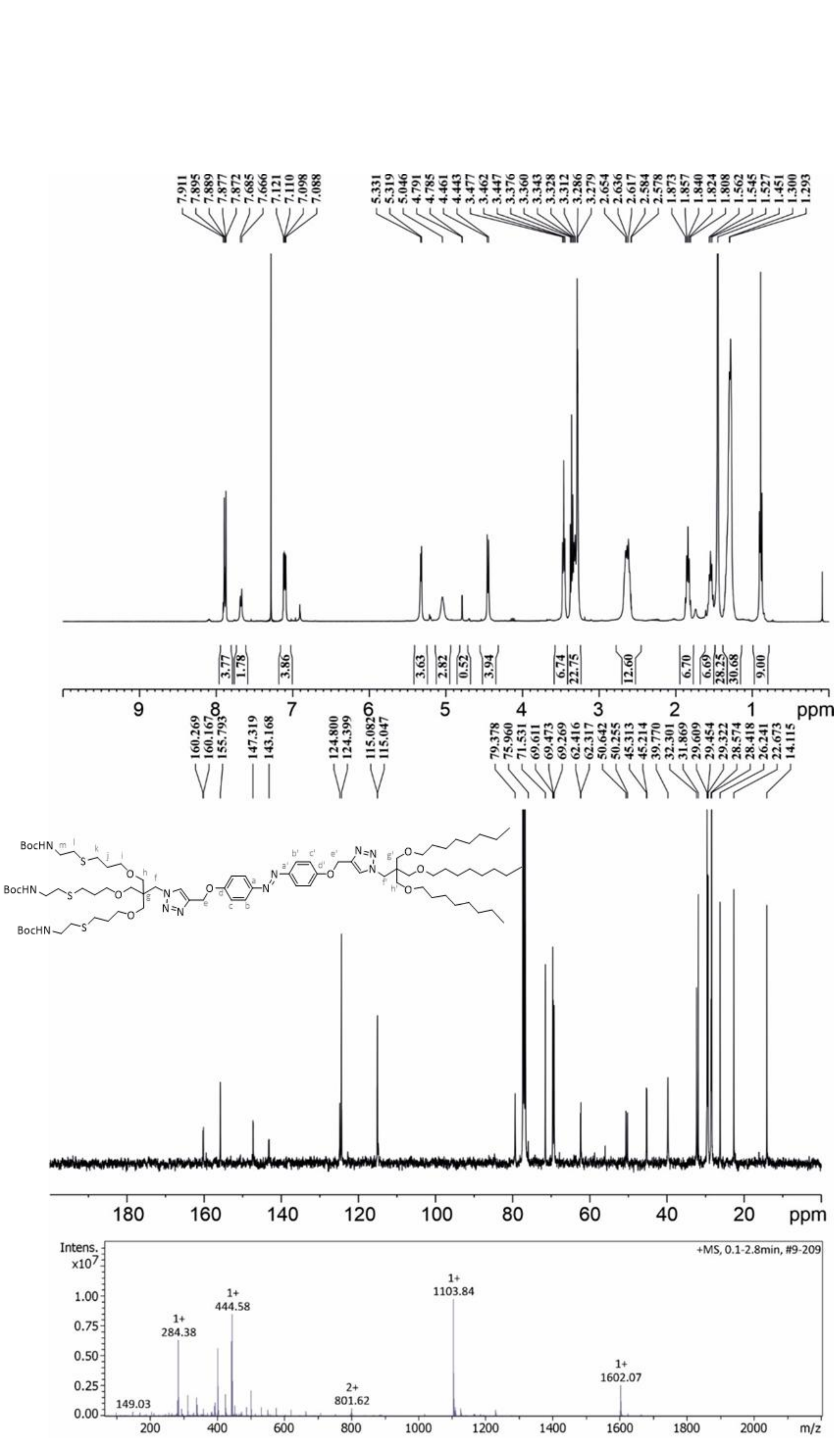

Figure S6. ^{1}H and ^{13}C NMR spectra (400 MHz, 100.6 MHz, $CDCl_3$) and ESIMS spectra of **13**.

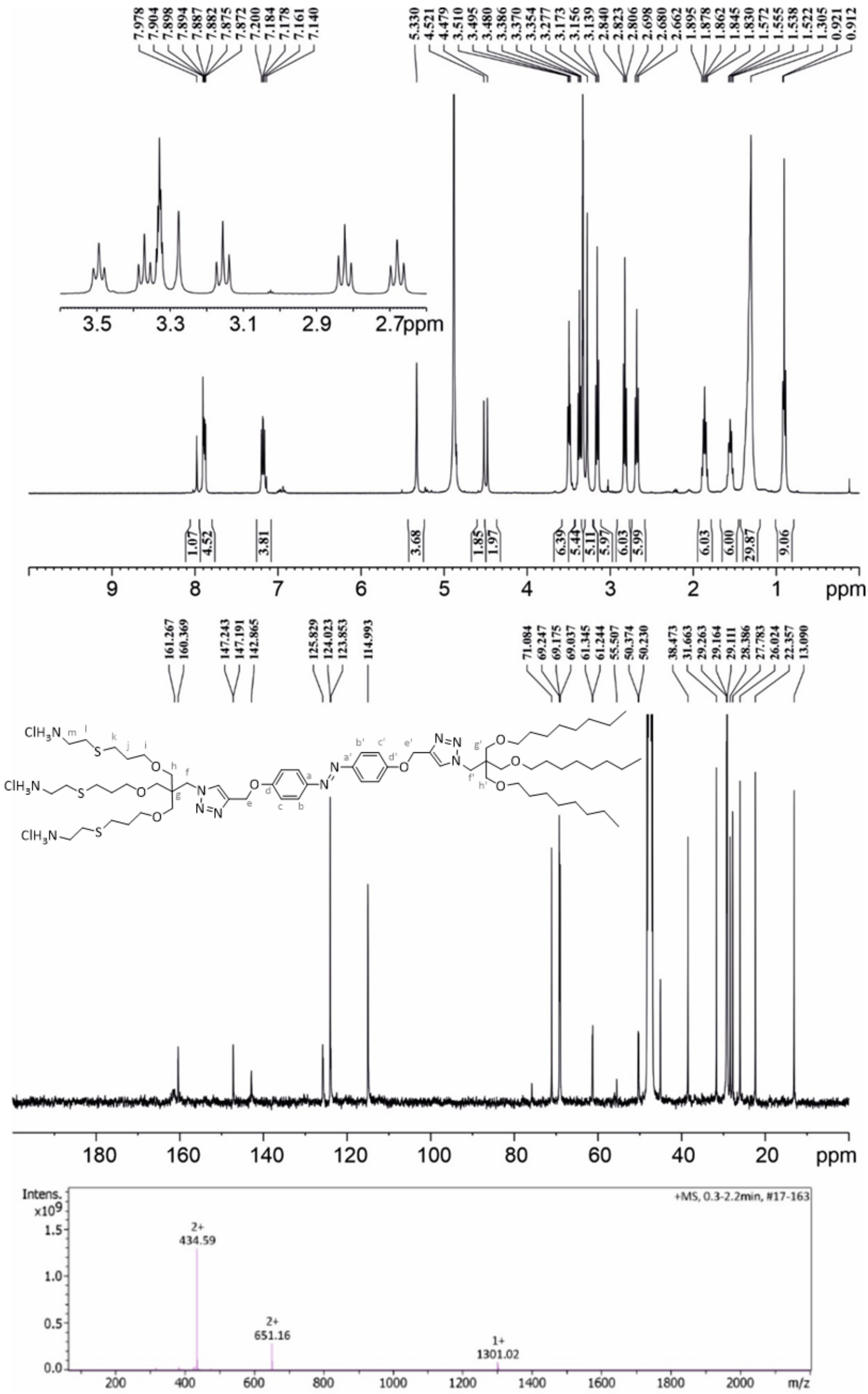

Figure S7. ^{1}H and ^{13}C NMR spectra (500 MHz, 125.7 MHz, CD_3OD) and ESIMS spectra of **1**.

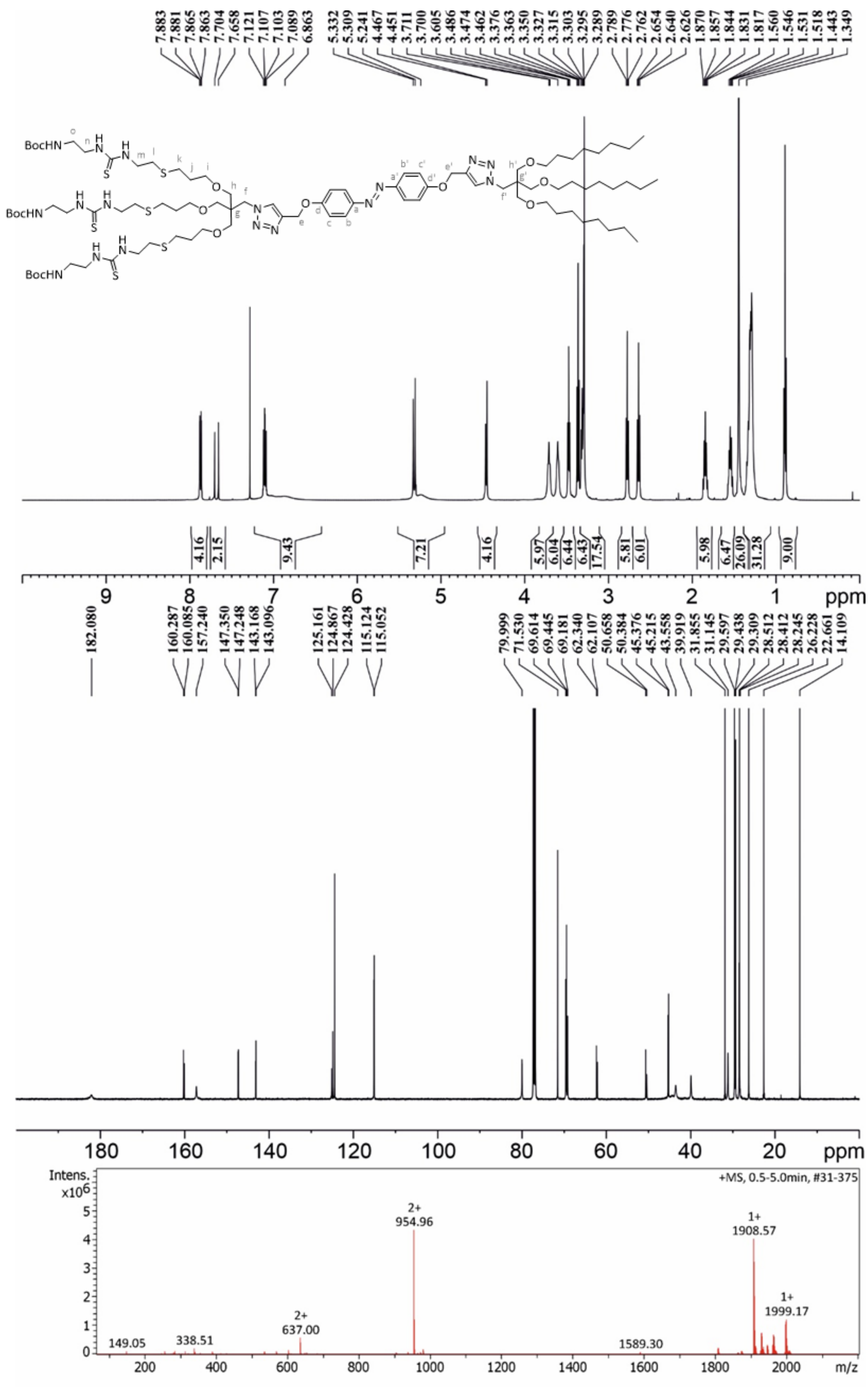

Figure S8. ^{1}H and ^{13}C NMR spectra (500 MHz (323 K), 125.7 MHz (298 K), $CDCl_3$) and ESIMS spectra of **17**.

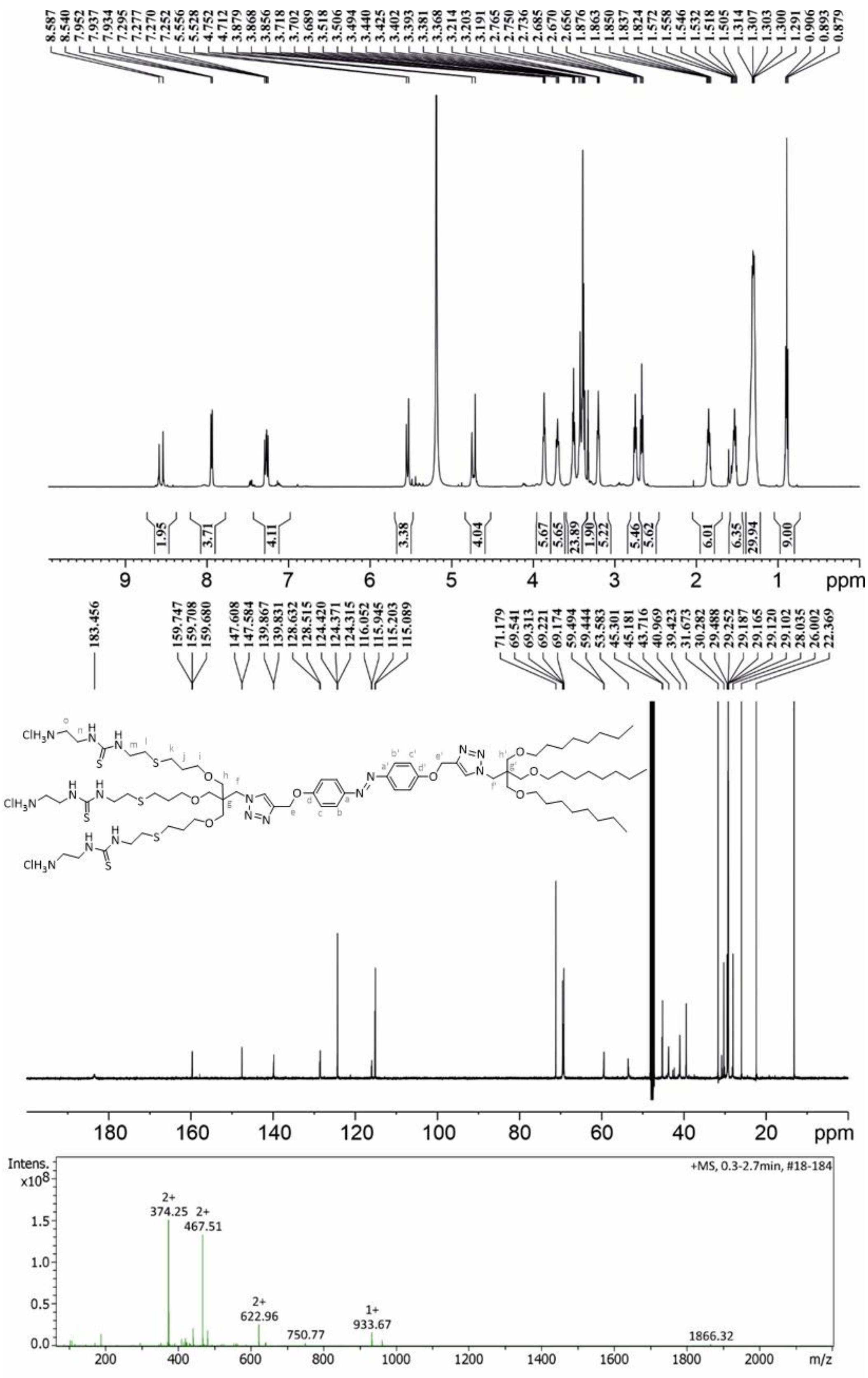

Figure S9. ^{1}H and ^{13}C NMR spectra (500 MHz, 125.7 MHz, CD_3OD) and ESIMS spectra of **2**.

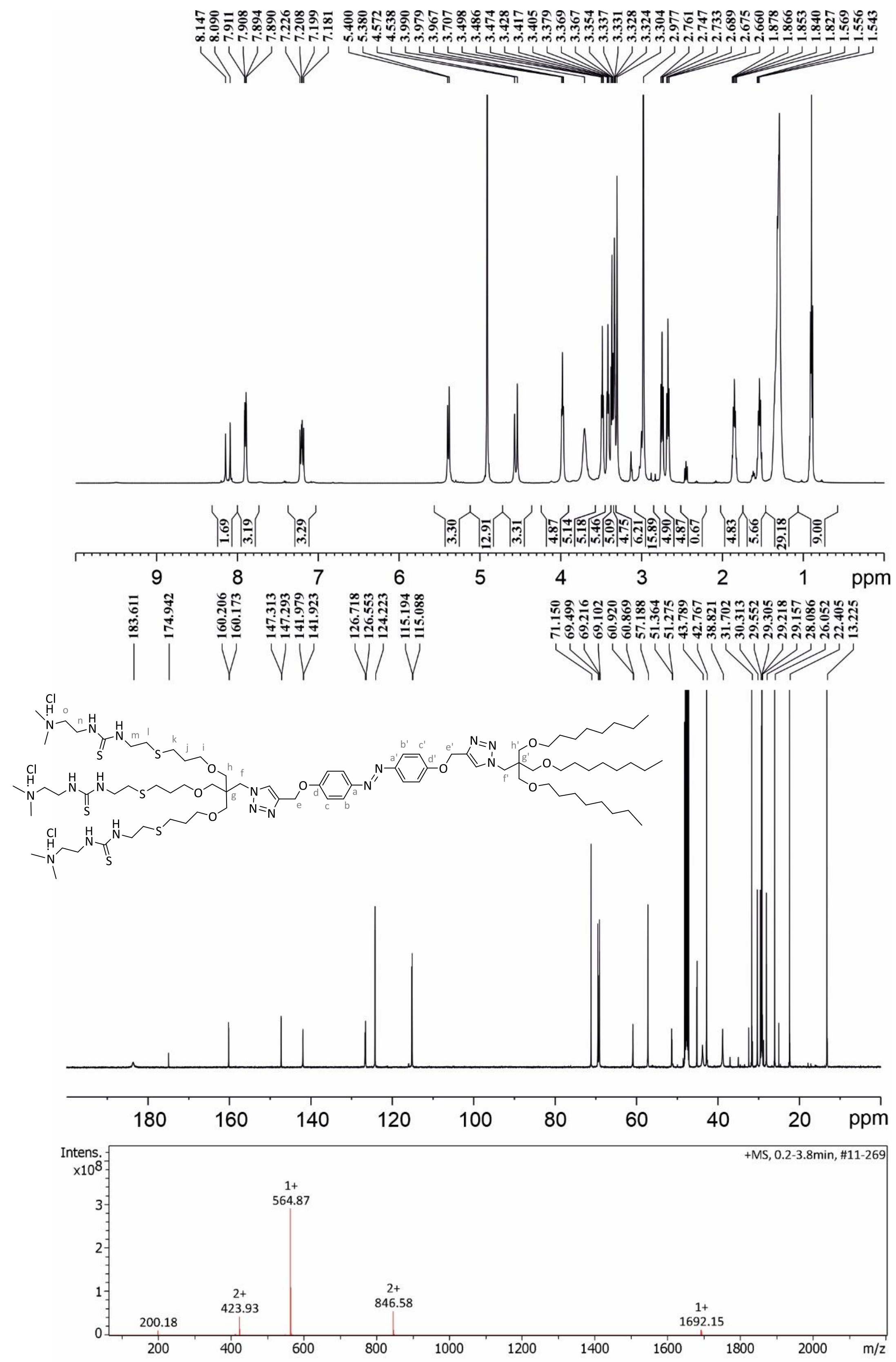

Figure S10. ^{1}H and ^{13}C NMR spectra (500 MHz, 125.7 MHz, CD_3OD) and ESIMS spectra of **3**.

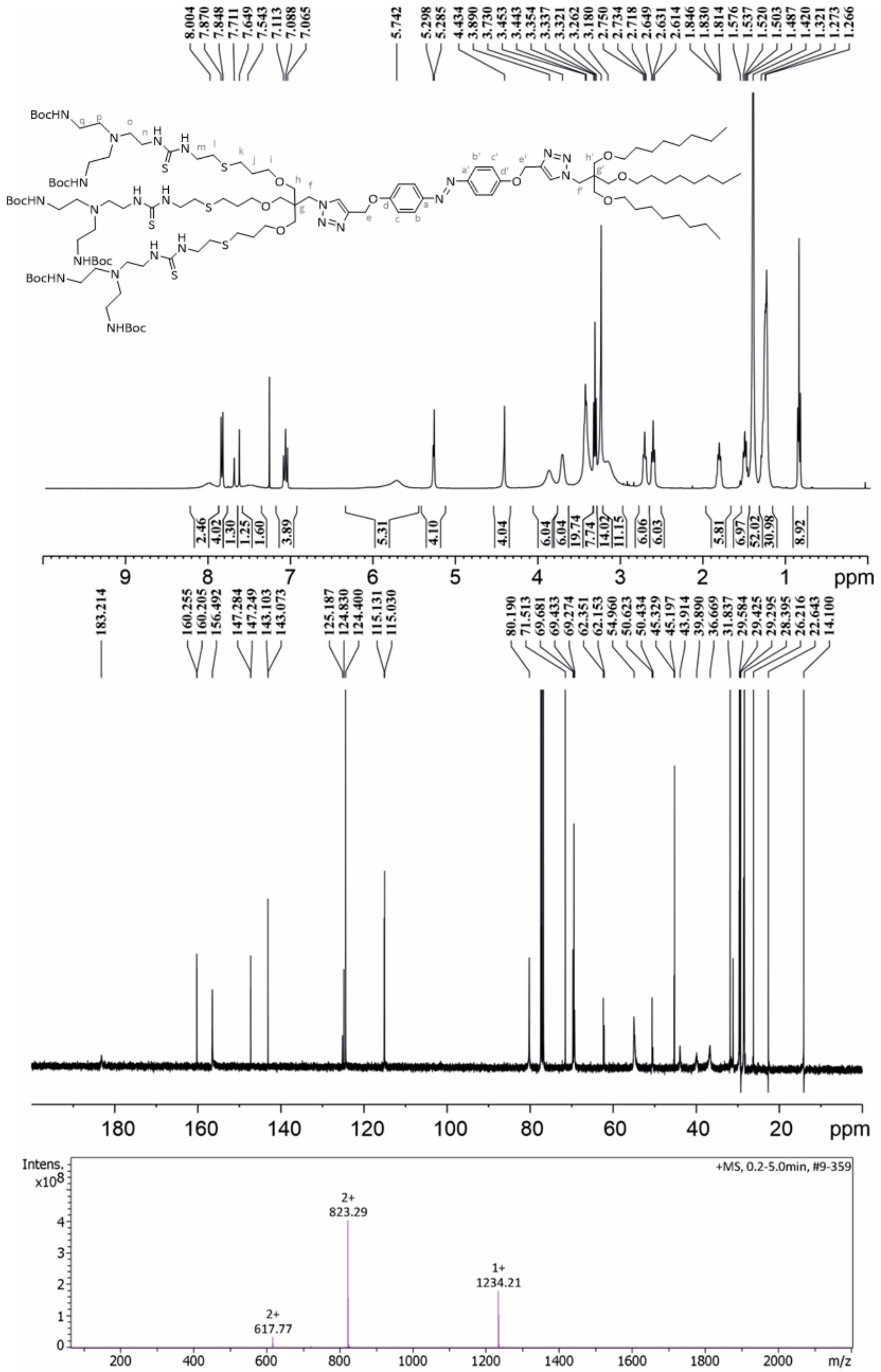

Figure S11. ^{1}H and ^{13}C NMR spectra (400 MHz, 100.6 MHz, $CDCl_3$) and ESIMS spectra of **19**.

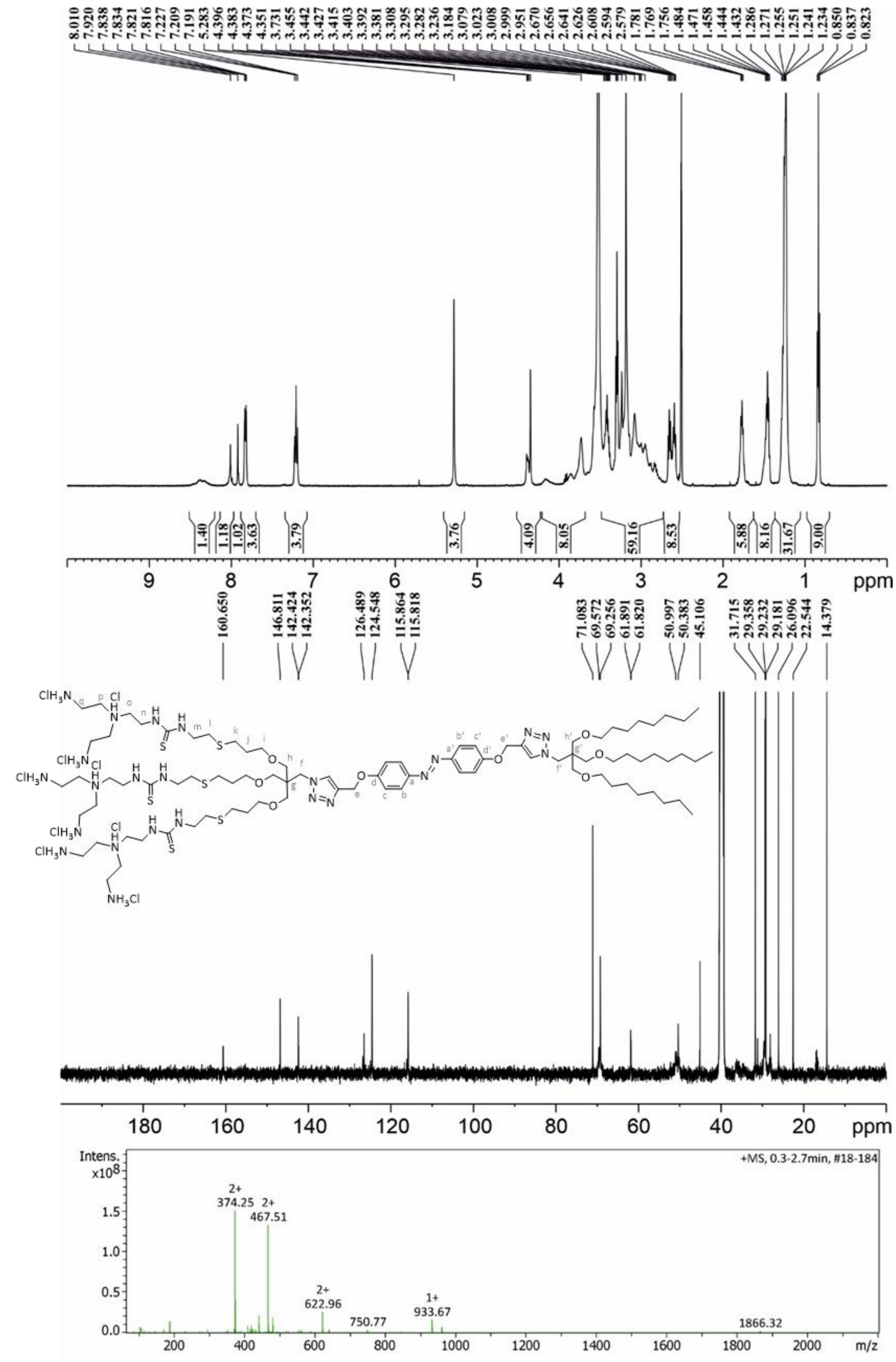

Figure S12. ^{1}H and ^{13}C NMR spectra (500 MHz (323 K), 125 MHz (298 K), $(CD_3)_2SO$) and ESIMS spectra of **4**.

4. UV-VIS SPECTROSCOPIC ABSORPTION MEASUREMENTS

General details on solvents and equipments

UV-Vis absorption spectroscopy measurements and experiments realized under light irradiations were performed in Milli-Q water and spectroscopic grade DMSO (Carlo Erba Reagents from DASIT Group). Spectra were recorded on Cary 100, Cary 4000 or Cary 5000 spectrophotometers from Agilent Technologies. Spectra were recorded with a scan speed 100 nm/min and 1 nm resolution with a time of response of 0.5 s. Unless otherwise stated, a pair of quartz cuvettes from Starna Scientific Ltd (type 23/Q/10; path length 10 mm) have been used for solution measurements.

General Procedure for photochromic reactions and power measurements

Photochromic reactions: photochromic reactions were performed in quartz cuvettes from Starna Scientific Ltd (type 23/Q/10; path length 10 mm), and induced *in situ* by a continuous irradiation with Hg/Xe lamp (Hamamatsu, LC6- or LC8-Lightningcure, 200 W) equipped with narrow band interference filters of appropriate wavelengths (Table S1):

Table S1 List of single-band bandpass filters commercially used in this work.

Irradiation wavelength [nm]	Filter[a]
365	Semrock Hg01-365/25, n°104
514	Semrock FF01-514/30-25, n°159

[a]Example of filter description: Semrock FF01-514/30-25, corresponds to a filter with a bandpass centred at 514 nm, a guaranteed minimum bandwidth of 30 nm, and a diameter of 25 mm; and the n° is the internal lab filter reference.

Power measurements: The incident lamp power was measured using an Ophir PD300-UV photodiode. The near-infrared (NIR) contribution to the photocurrent (PLP) was quantified and subtracted from the total signal (PTotal) by inserting a Schott long-pass filter (LP-585/2-73 nm) during 365 nm and 514 nm irradiations, which are transmitted through the Semrock filter. A transmittance of 90% was assumed in these corrections. (equation S1):

$$P_{\lambda-Irr} = P_{Total} - \left(\frac{10}{9} \cdot P_{LP}\right) \qquad \textbf{\textit{(Equation S1)}}$$

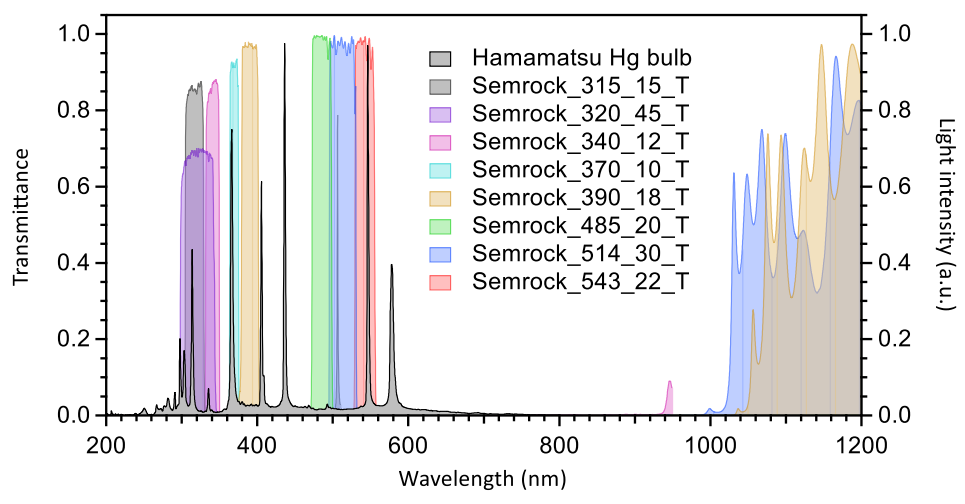

Figure S13. Hamamatsu Hg/Xe lamp emission spectrum and examples of transmission spectrum of different Semrock filters.

General Procedure for determination of the molar absorption coefficients (ε) of *E* isomer.

A stock solution (mother solution) of the *E* isomer of each compound was extemporaneously prepared in H_2O-DMSO (9:1) at the specific concentration indicated for each case.

From this stock, nine daughter solutions were prepared using 3 different micropipettes (Pipetman® Gilson) to cover a concentration range of 1-53 μM. Their absorbance was measured independently and immediately after preparation by UV-Vis spectroscopy at room temperature (22 ± 1 °C). To evaluate reproducibility and assess measurement accuracy, each spectrum was normalized by dividing the recorded absorbance by its corresponding concentration; only the overlapping normalized spectra were used to determine the molar absorption coefficient and obtain the corresponding spectrum in epsilon scale. The absorbance at the maximum of the *π*–*π** transition (357-360 nm) was then used to calculate the molar absorption coefficients by linear regression (least-squares method) constrained to pass through the origin (*x*- and *y*-intercept = 0). The molar absorption coefficient was obtained according to the Beer–Lambert law (**Equation S2**):

$$\mathrm{Abs}(\lambda) = \varepsilon(\lambda)\cdot l\cdot c \qquad (\textit{Equation S2})$$

where Abs(λ) represents the absorbance at the wavelength λ; ε(λ) represents the molar absorption coefficient at the wavelength λ, in $mol\cdot L^{-1}\cdot cm^{-1}$; l is the distance of the trajectory in the media, in cm; and *c* is the concentration of the substrate in $mol\cdot L^{-1}$.

The data are presented in Figures S14-S17 and Table S2.

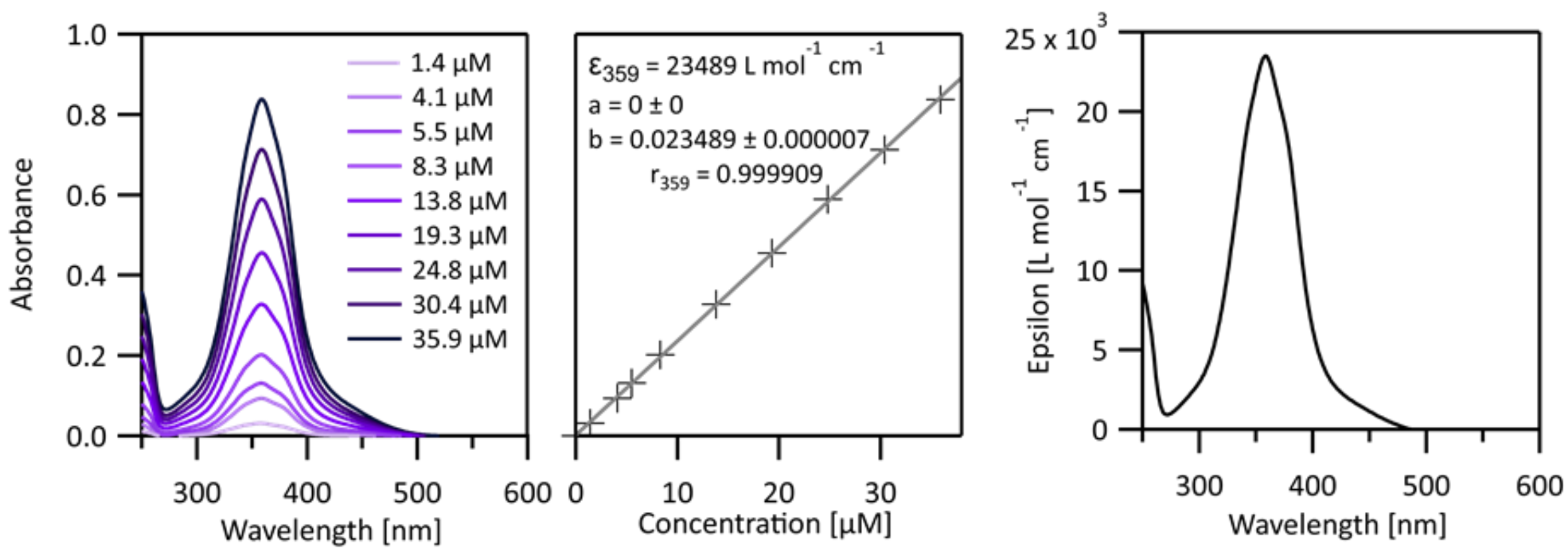

Figure S14. UV-Vis absorption spectra of the daughter solutions of **1-(*E*)** measured in H_2O-DMSO (9:1) (CARY 5000) (left) and evolution of the absorbance as a function of the concentration at the maximum of the π-π* band of *E*-isomer (359 nm) (center). Normalized UV-Vis absorption spectra of the average epsilon (right). Mother solution: 2.920 mg dissolved in 3000 μL of H_2O-DMSO (9:1).

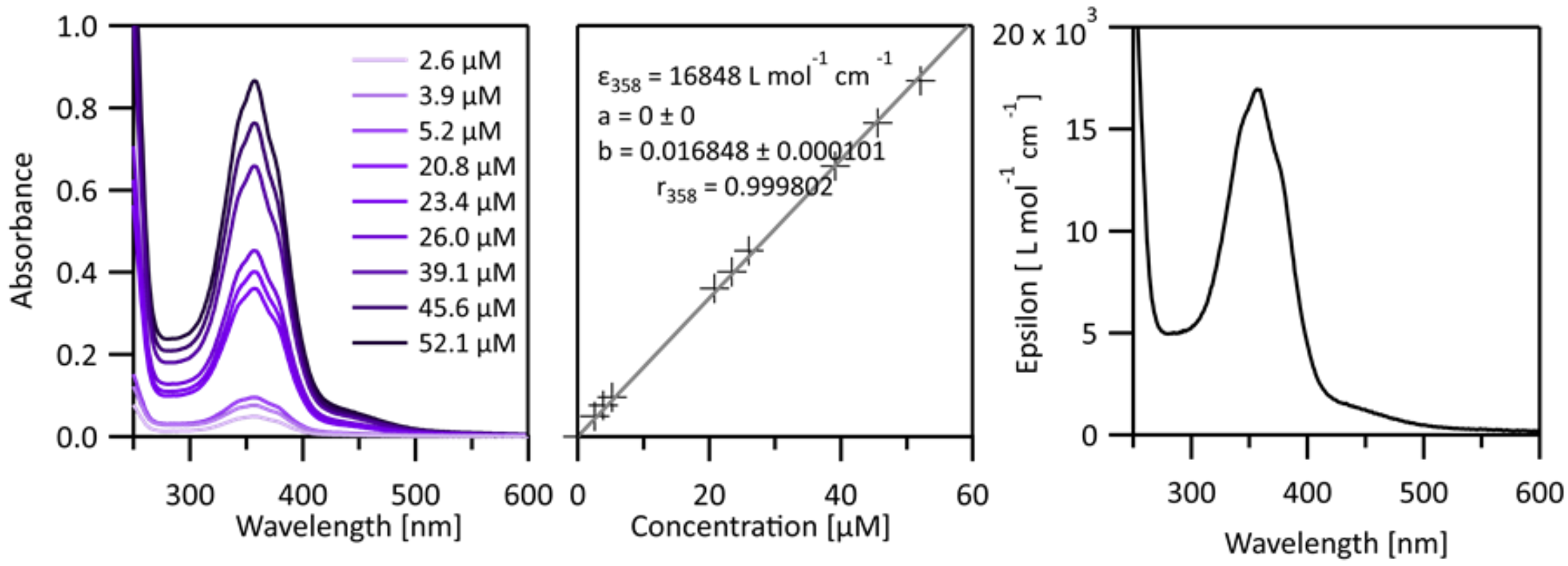

Figure S15. UV-Vis absorption spectra of the daughter solutions of **2-(*E*)** measured in H_2O-DMSO (9:1) (CARY 100) (left) and evolution of the absorbance as a function of the concentration at the maximum of the π-π* band of *E*-isomer (358 nm) (center). Normalized UV-Vis absorption spectra of the average epsilon (right). Mother solution: 3.352 mg dissolved in 3000 µL of H_2O-DMSO (9:1).

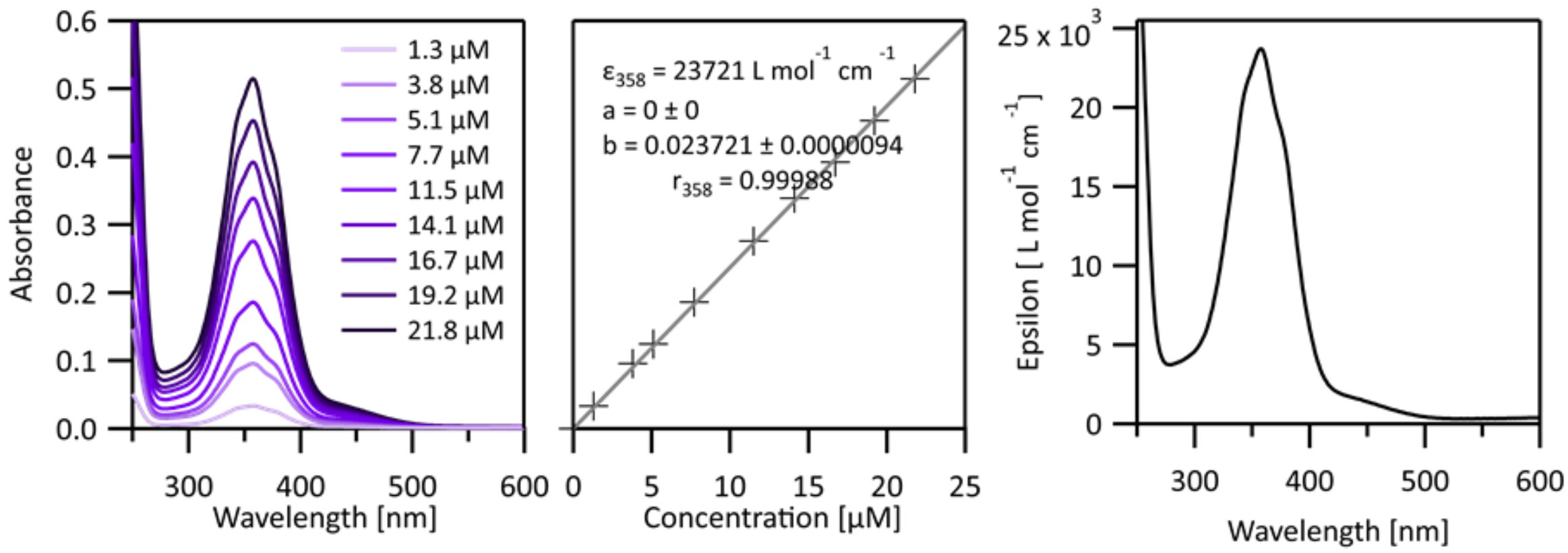

Figure S16. UV-Vis absorption spectra of the daughter solutions of **3-(*E*)** measured in H_2O-DMSO (9:1) (CARY 5000) (left) and evolution of the absorbance as a function of the concentration at the maximum of the π-π* band of *E*-isomer (358 nm). UV-Vis absorption spectra of the average epsilon (right). Epsilon was measured twice. Mother solution: 3.463 mg dissolved in 3000 µL of H_2O-DMSO (9:1).

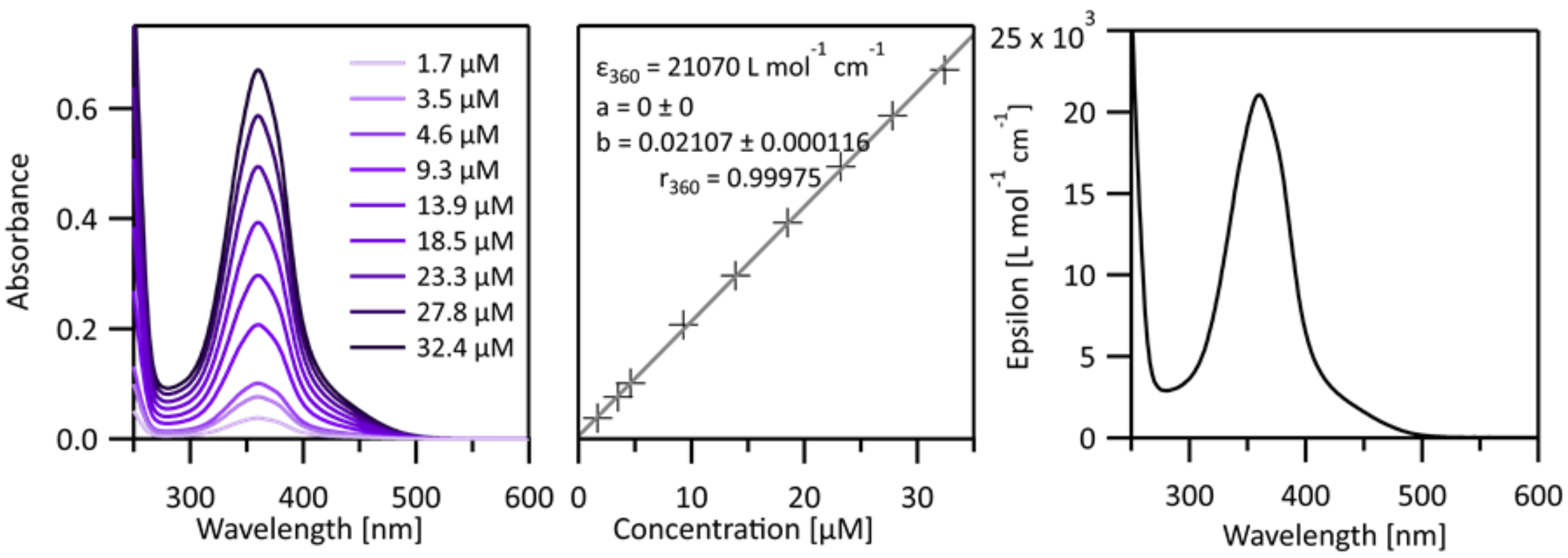

Figure S17. UV-Vis absorption spectra of the daughter solutions of **4-(*E*)** measured in H_2O-DMSO (9:1) (CARY 5000) (left) and evolution of the absorbance as a function of the concentration at the maximum of the π-π* band of *E*-isomer (360 nm) (center). Normalized UV-Vis absorption spectra of the average epsilon (right). Mother solution: 3.623 mg dissolved in 3000 µL of H_2O-DMSO (9:1).

Table S2. Characteristic λ (nm), molar absorption coefficient (ε), half-life ($\tau_{1/2}$), and photoconversion yield (CY) for **1-4** in H_2O-DMSO (9:1).

		**1**	**2**	**3**	**4**
λ_{max} [nm] (transition)	***E*** **(π→π*)**	359	358	357	360
	Z (n→π*)	447	448	449	444
**Isosbestic points** [nm]		319, 422	318, 422	319, 422	320, 426
**ε** [L·mol^{-1}·cm^{-1}] at *E* isomer λ_{max}	***E***	23489	16848	23721	21070
$\tau_{1/2}$ [min] at 37 °C		168	121	120	26
**CY** [% *Z*-isomer]	***E→Z*** (irr 365 nm)	99	99	99	95
	Z→E (irr 514 nm)	97	98	96	99

General Procedure for the Determination of the Half-Life of *Z*-isomers

To determine the half-life of the *Z* isomers of the Azo-IAJD derivatives in the dark, the thermal $Z \rightarrow E$ isomerization was monitored by recording UV–Vis absorption spectra at 37 ± 1 °C. The resulting time-dependent absorption profiles were then fitted using the first-order rate Equation S3:

$$Abs\ (t) = A_o - (A_o - A_i)e^{-kt} \quad \textbf{\textit{(Equation S3)}}$$

where

- A_o, is the absorption of original *E*-isomer (fixed or free parameter)
- A_i, is the absorption at the PSS_{365}
- k, is the rate constant of thermal isomerization
- t, is the time in minutes

After fitting, the rate constant k_{310} (37 ºC, 310.15 Kelvin) was obtained from the fitting parameters, and the half-life, $t_{1/2}$ (min), was then calculated according to Equation S4:

$$t_{1/2} = \frac{ln\ (2)}{k} \qquad \textbf{\textit{(Equation S4)}}$$

The data are presented in Figure S18-S21 and Table S2.

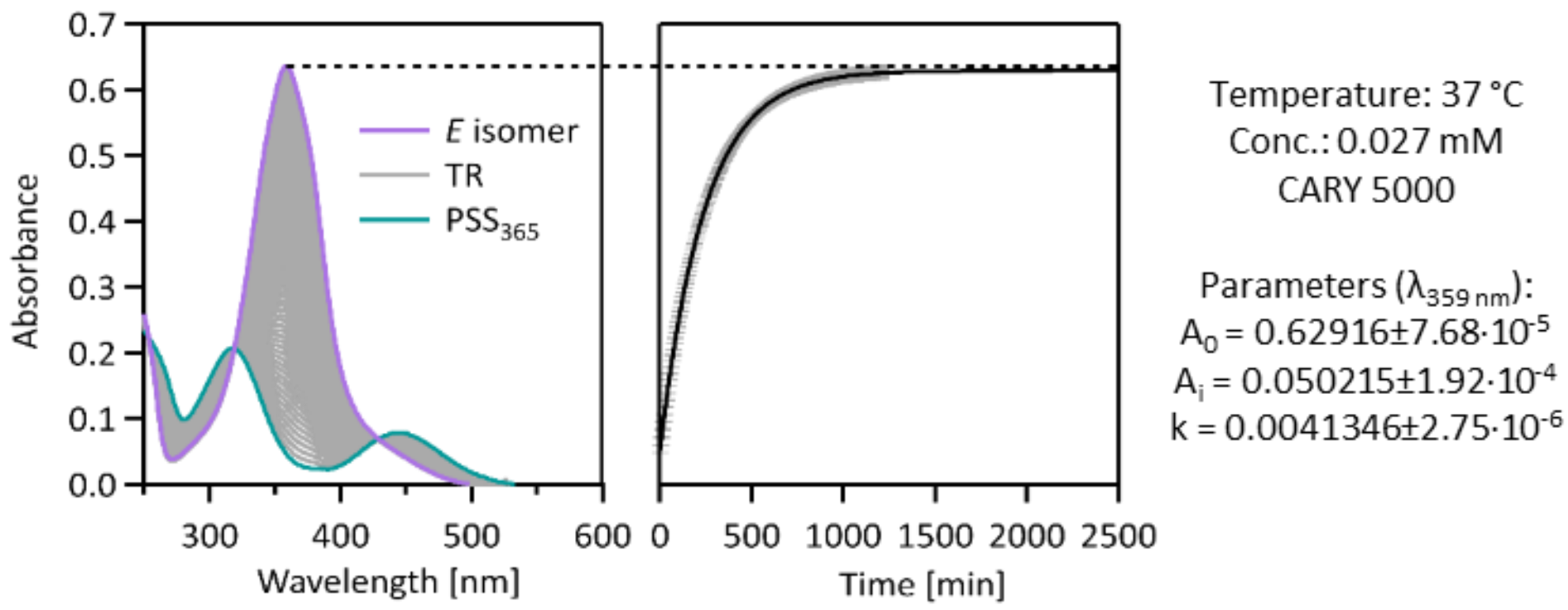

Figure S18. UV-Vis absorption spectra of the thermal return (TR) of a solution 0.027 mM of **1** ($Z \rightarrow E$) in H_2O-DMSO (9:1) at 37 ºC (CARY 5000) (left), and curve fitting following the absorbance at 359 nm (right).

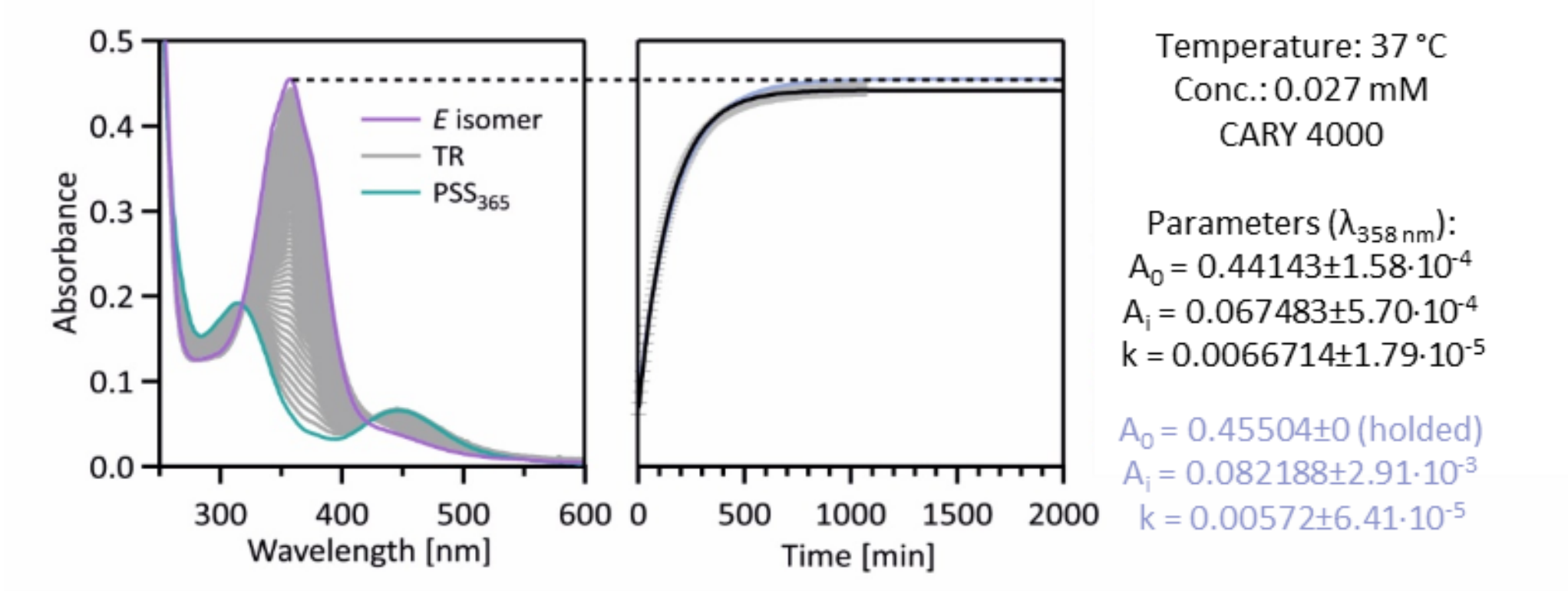

Figure S19. UV-Vis absorption spectra of the thermal return (TR) of a solution 0.027 mM of **2** (*Z* → *E*) in H_2O-DMSO (9:1) at 37 ºC (CARY 4000) (left), and curve fitting following the absorbance at 358 nm. A_0 was holded for a better adjustment (right).

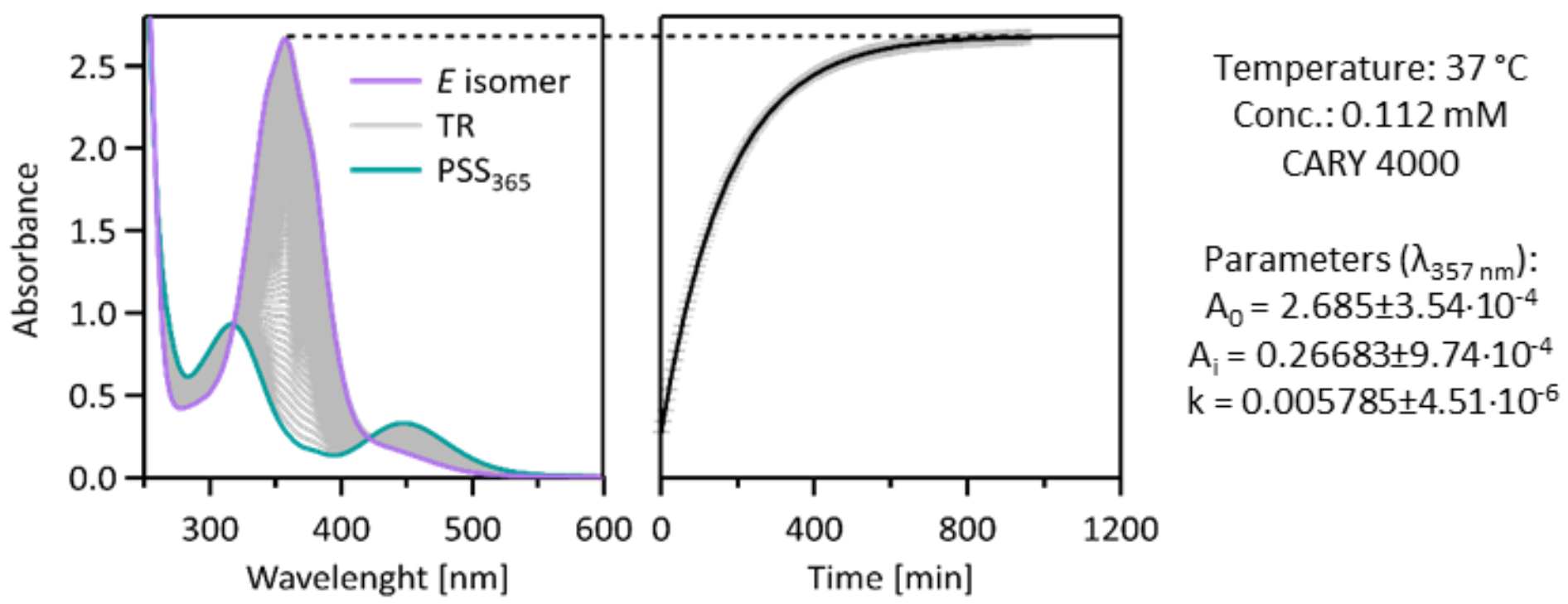

Figure S20. UV-Vis absorption spectra of the thermal return (TR) of a solution 0.112 mM of **3** (*Z* → *E*) in H_2O-DMSO (9:1) at 37 ºC (CARY 4000) (left), and curve fitting following the absorbance at 357 nm (right).

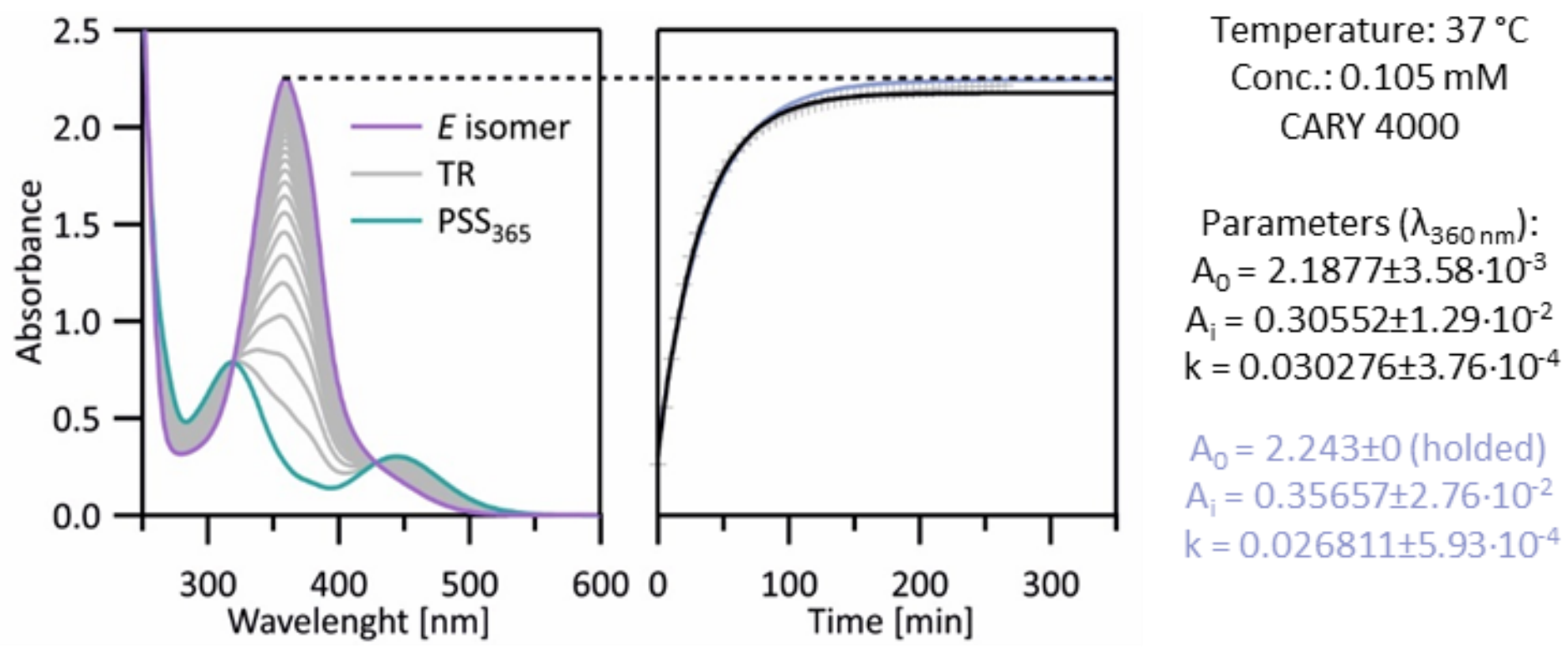

Figure S21. UV-Vis absorption spectra of the thermal return (TR) of a solution 0.105 mM of **4** (*Z* → *E*) in H_2O-DMSO (9:1) at 37 ºC (CARY 4000) (left), and curve fitting following the absorbance at 360 nm. A_0 was holded for a better adjustment (right).

General Procedure for the Determination of Photoconversion yields.

^{1}H NMR spectra were recorded on a JEOL ECZL-400 spectrometer (399.78 MHz for ^{1}H) equipped with an autotunable broadband probe, and using Delta 6 software.

An extemporaneously prepared solution of the *E*-azobenzene derivative was realized in D_2O-$(CD_3)_2SO$ (9:1) at a concentration that provide an absorbance of 1.5–4.0 at the π–π* band. This range ensured both (i) sufficient concentration for NMR measurements and (ii) compliance with the spectrophotometer's linear absorbance limit at the compound's λ_{max}. After recording the absorbance, an aliquot of the solution was taken for ^{1}H NMR analysis (relaxation delay 2 s; 512–1024 scans). Owing to aggregation, this aliquot was diluted with CD_3OD to obtain a 45:5:50 $D_2O/(CD_3)_2SO/CD_3OD$ mixture; ensuring clearer signals for NMR integrations.

The remaining solution was then irradiated at 365 nm to induce $E \rightarrow Z$ isomerization and reach the photostationary state (PSS_{365}). A second aliquot was taken, diluted with CD_3OD under the same conditions, and analyzed by ^{1}H NMR. To monitor possible thermal $Z \rightarrow E$ back-isomerization during the lengthy NMR acquisition, the initial 9:1 D_2O-$(CD_3)_2SO$ solution kept in the spectrophotometer was measured by UV–Vis spectroscopy immediately before and after each NMR measurement. The experiment was subsequently repeated at PSS_{514} ($Z \rightarrow E$ isomerization). In both cases, the PSS was confirmed by two consecutive irradiation providing UV–Vis spectra showing a complete overlap.

Photoconversion yields were determined by integrating diagnostic ^{1}H NMR signals of each isomer. For compounds **7** and **9**, faster thermal relaxation compromised accuracy; therefore, the integrated NMR values represent an average derived from UV–Vis spectra collected immediately before and after NMR acquisition. Consequently, conversion yields for these compounds were calculated using the linear correlation (Eq. S5) between absorbance at the π–π* band and the percentage of *Z* isomer determined by NMR, using the known absorbance of the PSS.

$$Abs_p = a + b \cdot CY \qquad \textbf{\textit{(Equation S5)}}$$

To select the appropriate wavelengths, various optical filters were evaluated to identify the PSS conditions that most closely approached the pure photochromic states for *E* or *Z* isomers (Figure S22). The data corresponding to the selected wavelengths are shown in Figures S23–S24 and Table S2.

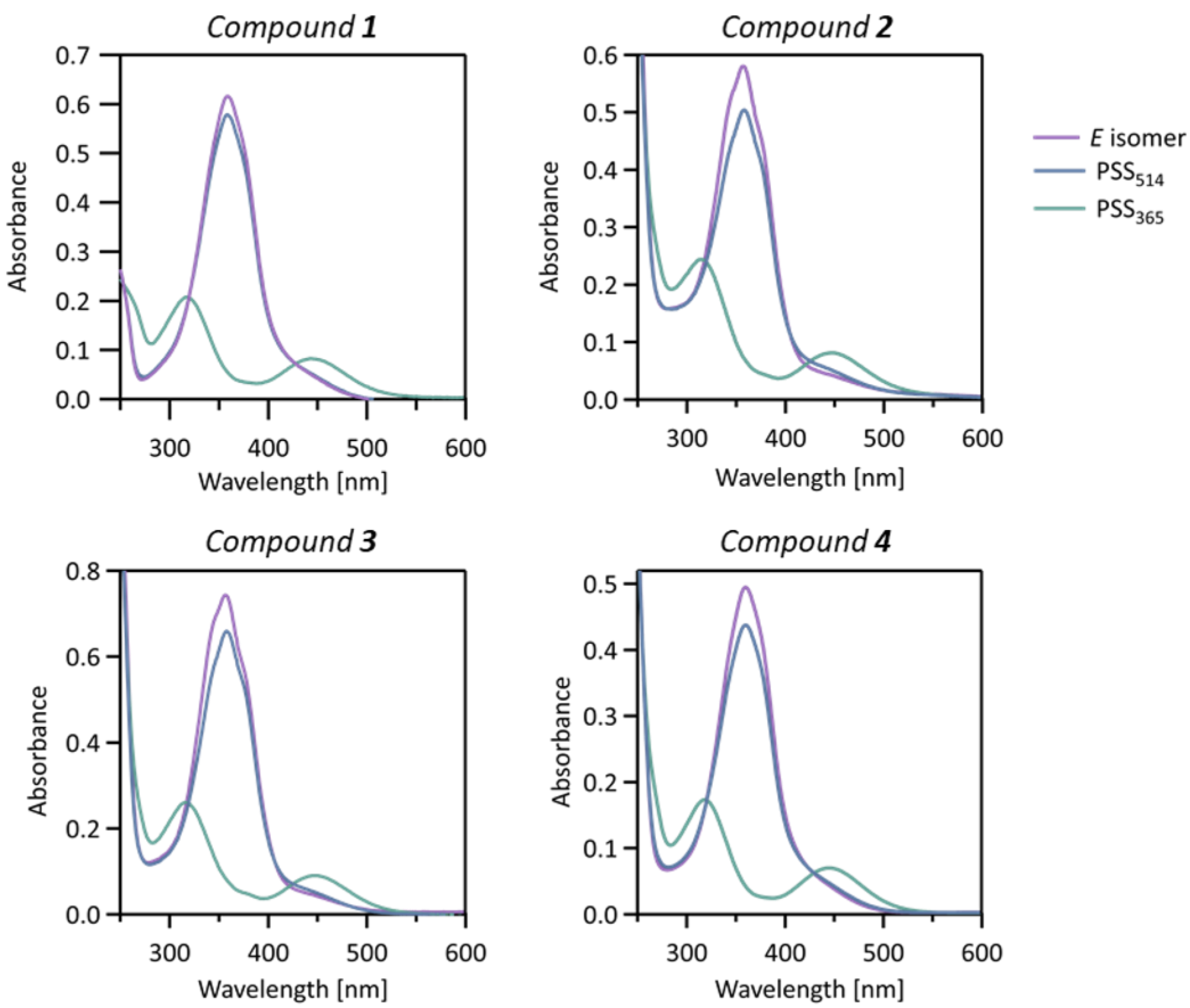

Figure S22. UV-Vis absorption spectra of **1** (0.026 mM), **2** (0.035 mM), **3** (0.031 mM) and **4** (0.024 mM), at different PSS in H_2O-DMSO (9:1).

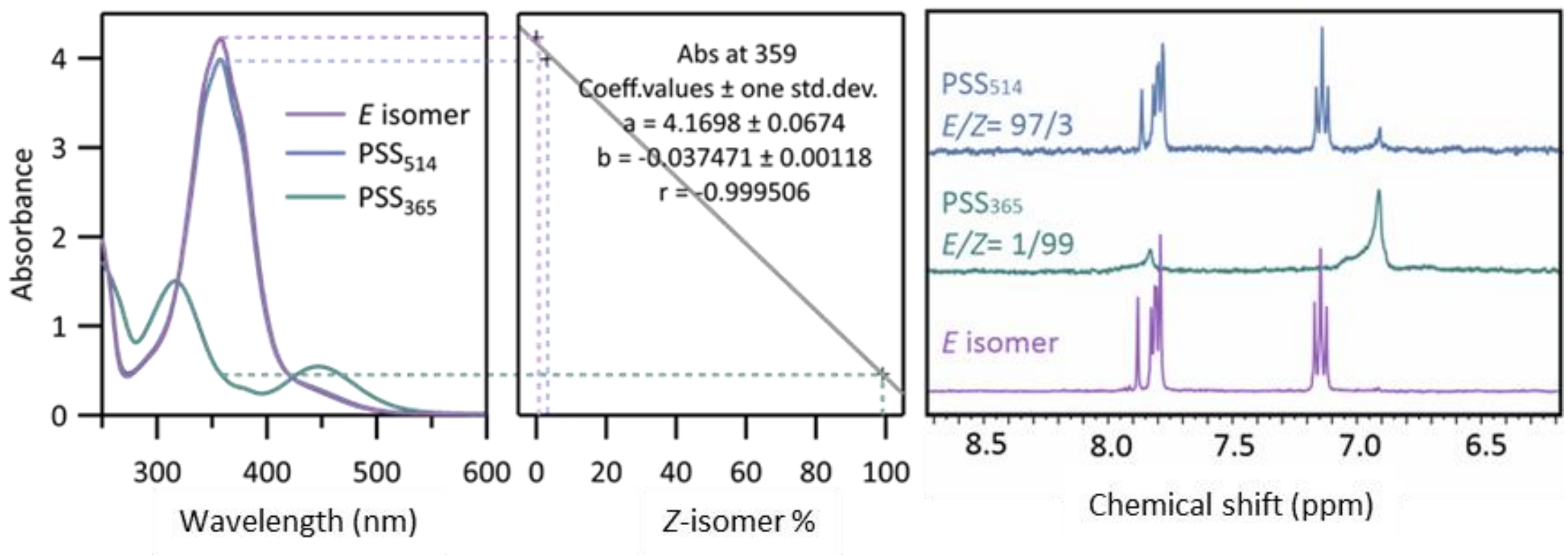

Figure S23. UV-Vis absorption (CARY 4000, 0.178 mM in D_2O-$(CD_3)_2SO$ (9:1); left) and ^1H NMR (400 MHz, 0.089 mM in D_2O-$(CD_3)_2SO$-CD_3OD (45:5:50); right) spectra of **1**. Linear regression obtained by plotting the absorbance at 359 nm as function *Z* isomer percentage obtained from NMR integration (center).

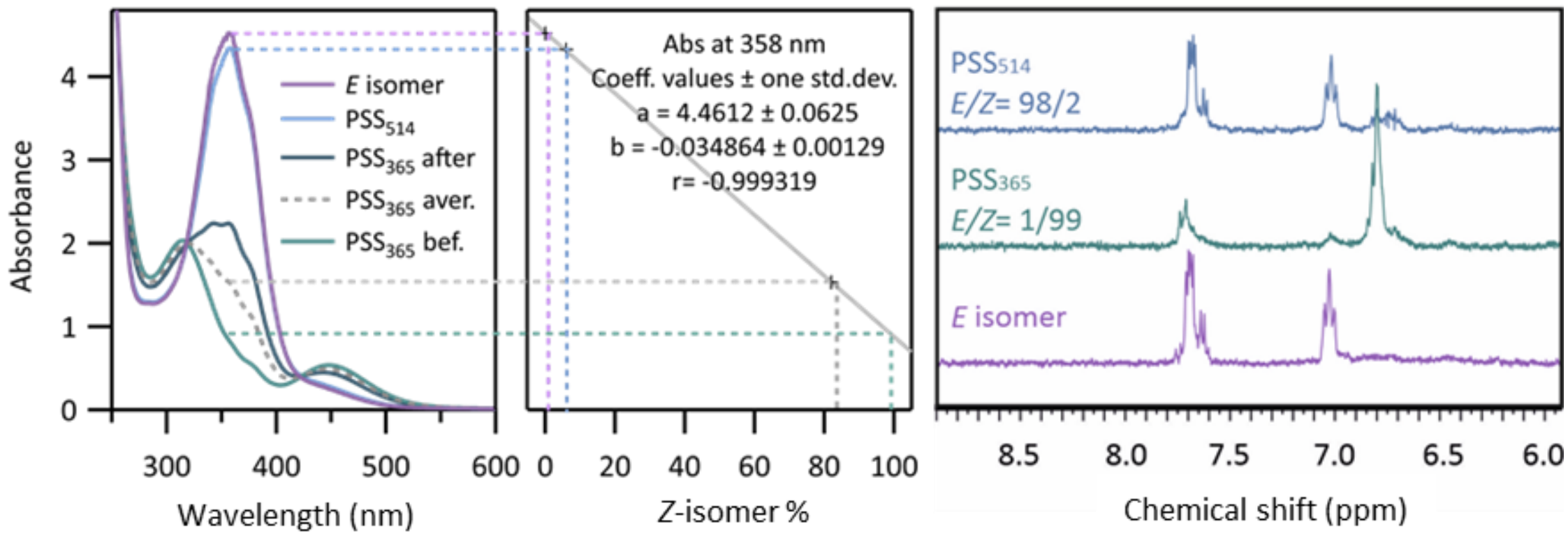

Figure S24. UV-Vis absorption (CARY 4000, 0.268 mM in D_2O-$(CD_3)_2SO$ (9:1); left) and ^1H NMR (400 MHz, 0.134 mM in D_2O-$(CD_3)_2SO$-CD_3OD (45:5:50); right) spectra of **2**. Linear regression obtained by plotting the absorbance at 358 nm as function *Z* isomer percentage obtained from NMR integration (center).

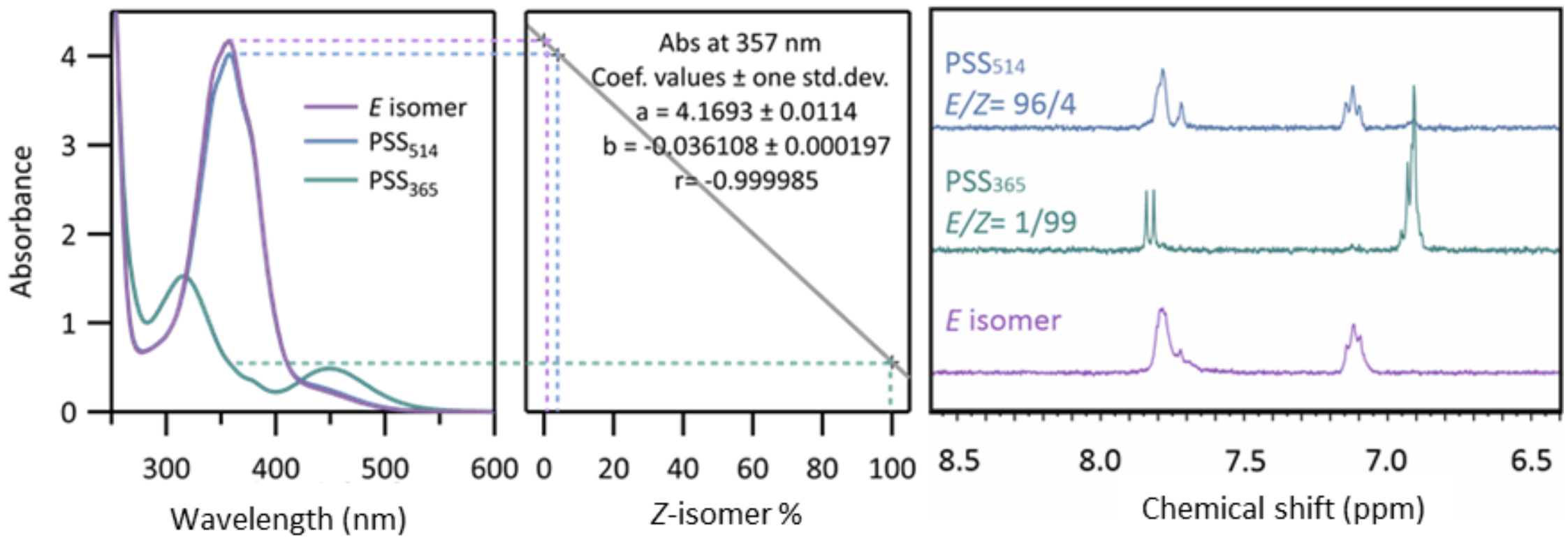

Figure S25. UV-Vis absorption (CARY 4000, 0.173 mM in D_2O-$(CD_3)_2SO$ (9:1), left) and ^1H NMR (400 MHz, 0.087 mM in D_2O-$(CD_3)_2SO$-CD_3OD (45:5:50); right) spectra of **3**. Linear regression obtained by plotting the absorbance at 357 nm as function *Z* isomer percentage obtained from NMR integration (center).

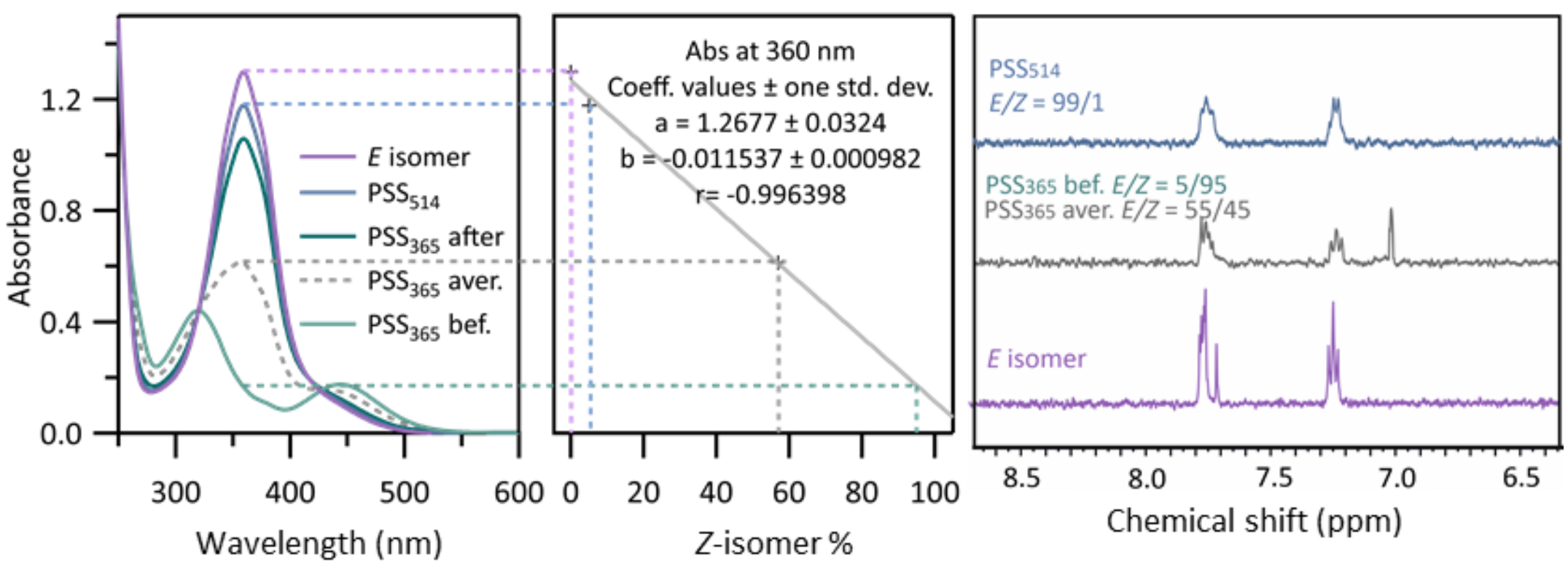

Figure S26. UV-Vis absorption (CARY 4000, 0.213 mM in D_2O-$(CD_3)_2SO$ (9:1); left) and NMR (400 MHz, 0.107 mM in D_2O-$(CD_3)_2SO$-CD_3OD (45:5:50); right) spectra of **4**. Linear regression obtained by plotting the absorbance at 360 nm as function *Z* isomer percentage obtained from NMR integration (center).

General Procedure for Fatigue Resistance Measurements

For fatigue resistance studies, a solution of the *E* isomer of each compound was freshly prepared in H_2O–DMSO (9:1). The solution was then subjected to sequential irradiation at 365 nm (green spectrum) and 514 nm (blue spectrum), defining one irradiation cycle. Absorbance was recorded before and after each illumination, and a minimum of ten consecutive cycles was performed for every compound.

The incident lamp power ($P_{\lambda irr}$) was measured and calculated according to Eq. S1 (section **General Procedure for photochromic reactions and power measurements**).

The data are presented in Figures S27-S30.

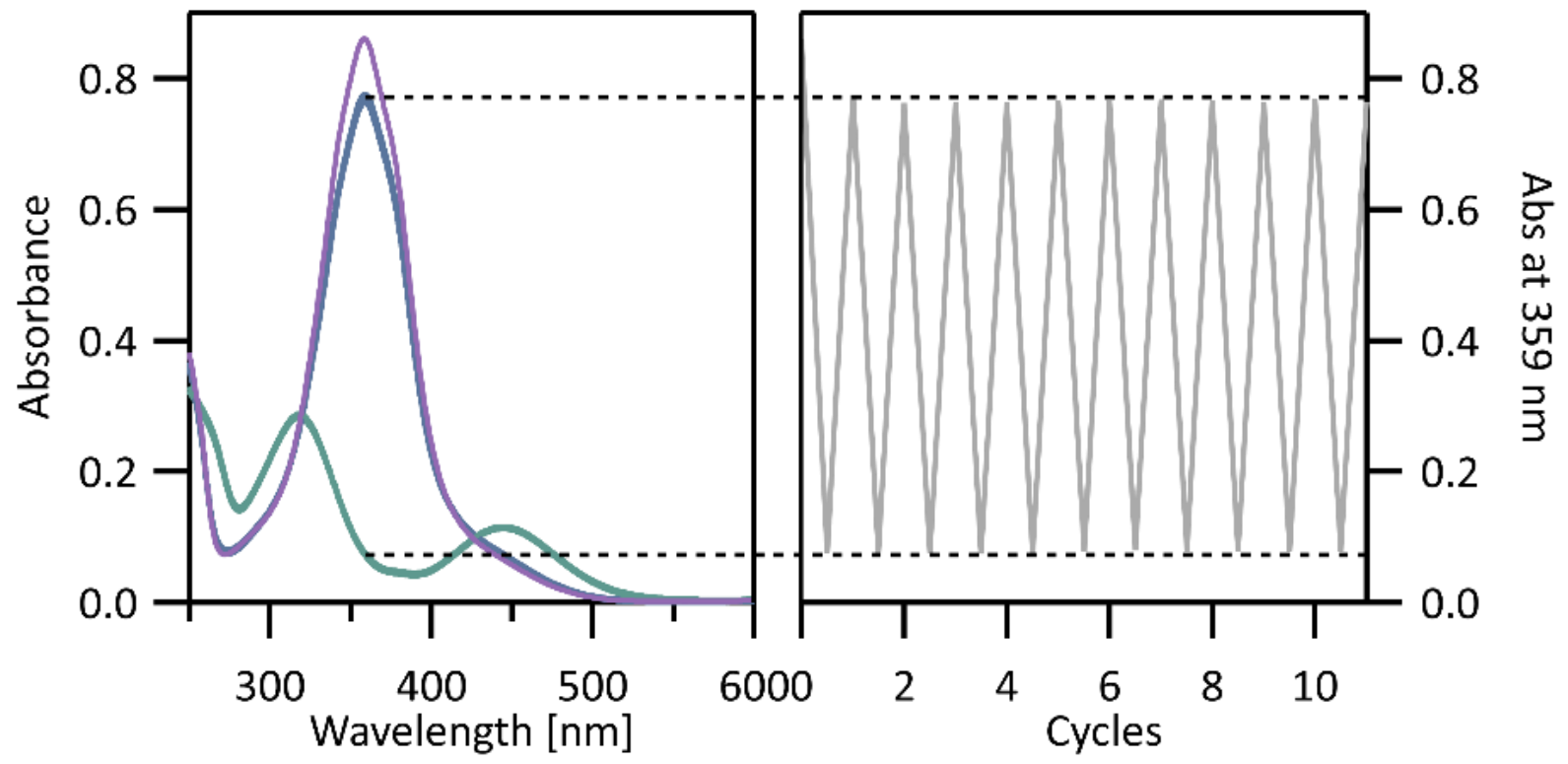

Figure S27. Fatigue resistance of a 0.036 mM solution of **1**-*E* (purple) after alternate irradiations at 365 nm (45 s; P_{365} = 19.1 mW cm^{-2}, green) and 514 nm (600 s; P_{514} = 4.2 mW cm^{-2}, blue) in H_2O-DMSO (9:1). Superimposition of all the spectra (CARY 4000, left graph) and absorbance at 359 nm as a function of the cycle number (right graph).

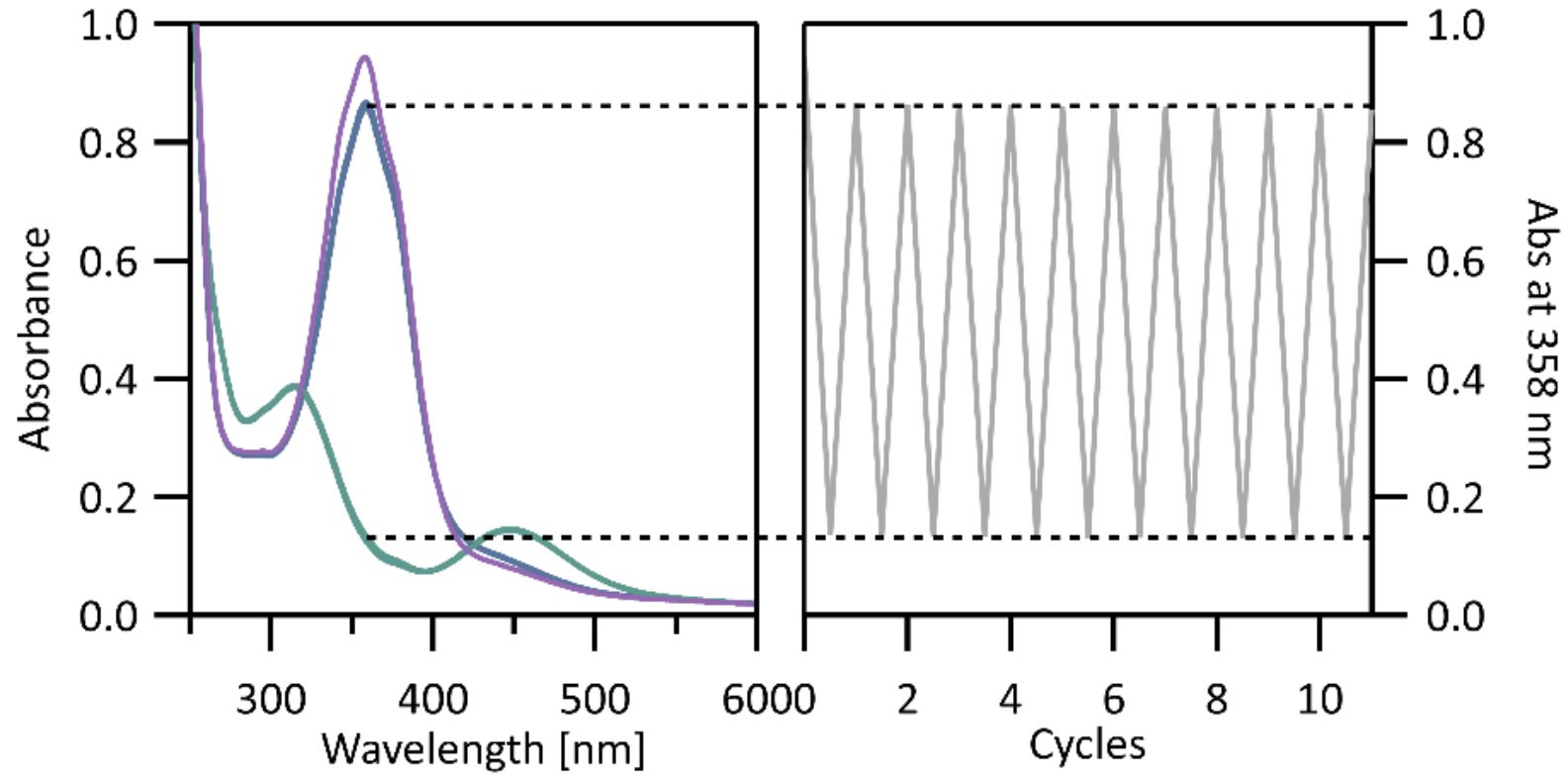

Figure S28. Fatigue resistance of a 0.058 mM solution of **2-*E*** (purple) after alternate irradiations at 365 nm (45 s; P_{365} = 82.7 mW cm^{-2}, green) and 514 nm (660 s; P_{514} = 18.4 mW cm^{-2}, blue) in H_2O-DMSO (9:1). Superimposition of all the spectra (CARY 4000, left graph) and absorbance at 358 nm as a function of the cycle number (right graph).

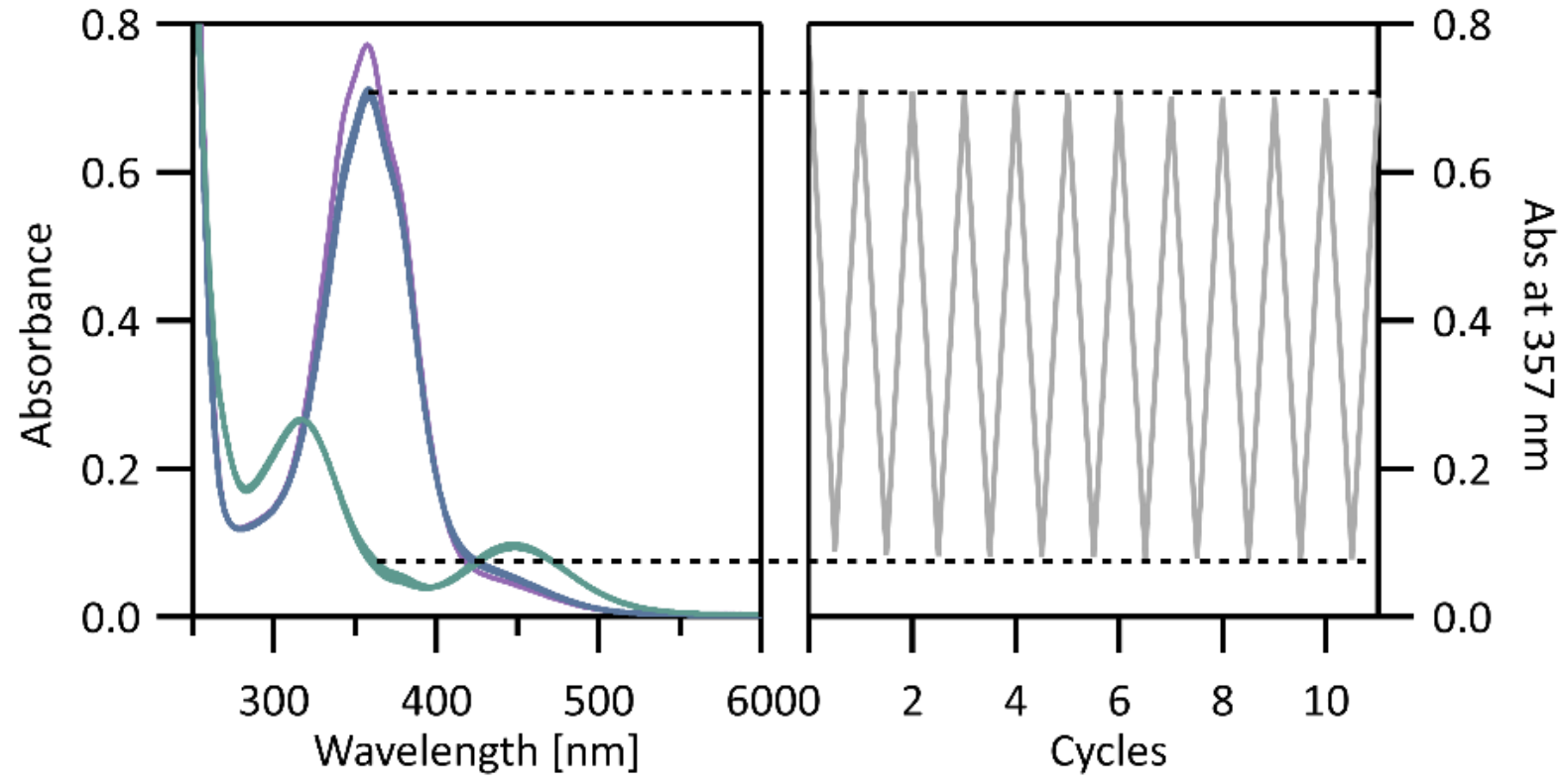

Figure S29. Fatigue resistance of a 0.032 mM solution of **3-*E*** (purple) after alternate irradiations at 365 nm (45 s; P_{365} = 92.0 mW cm^{-2}, green) and 514 nm (660 s; P_{514} = 19.2 mW cm^{-2}, blue) in H_2O-DMSO (9:1). Superimposition of all the spectra (CARY 4000, left graph) and absorbance at 357 nm as a function of the cycle number (right graph).

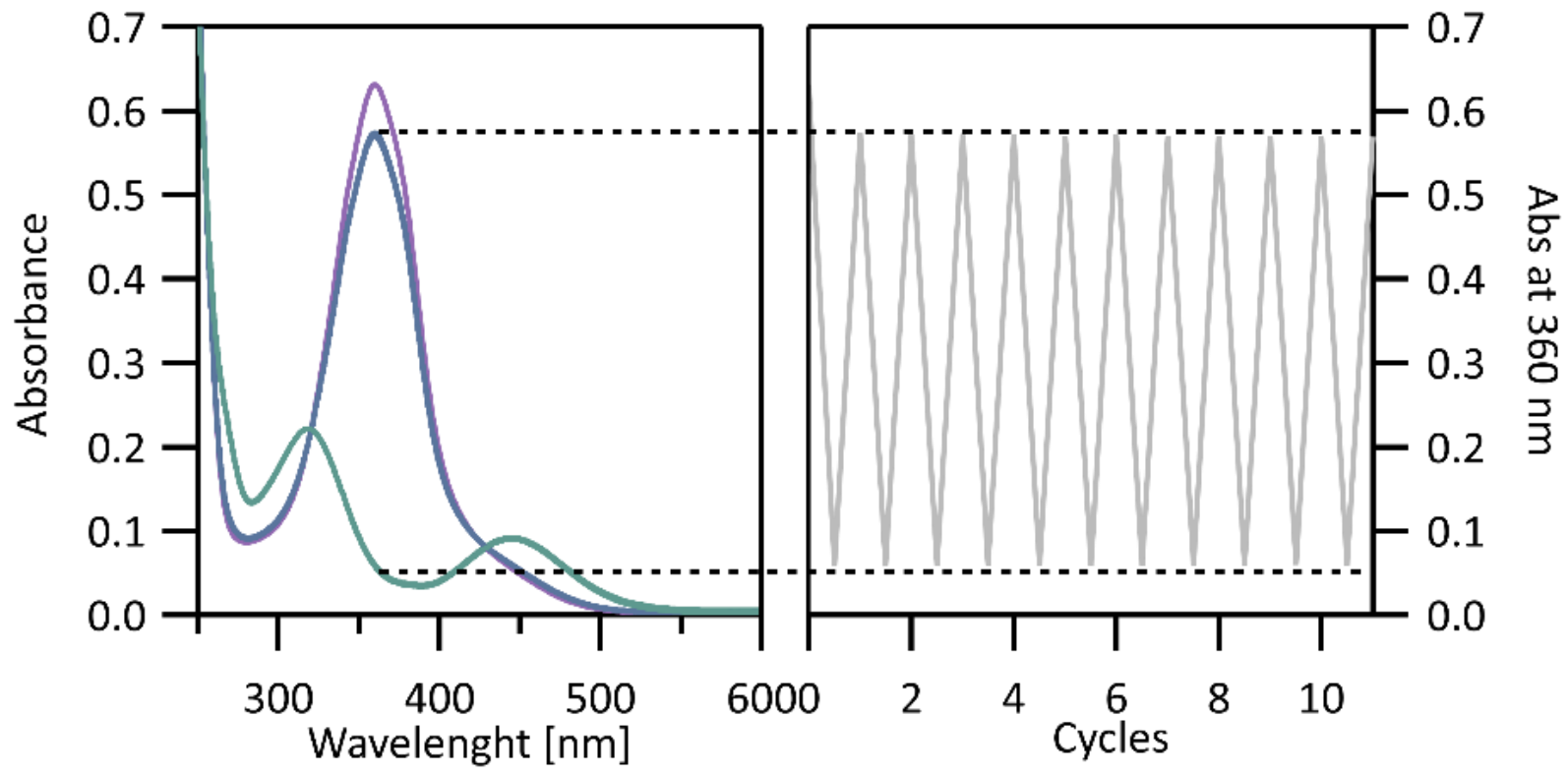

Figure S30. Fatigue resistance of a 0.031 mM solution of **4-*E*** (purple) after alternate irradiations at 365 nm (45 s; P_{365} = 73.2 mW cm^{-2}, green) and 514 nm (660 s; P_{514} = 16.2 mW cm^{-2}, blue) in H_2O-DMSO (9:1). Superimposition of all the spectra (CARY 4000, left graph) and absorbance at 360 nm as a function of the cycle number (right graph).

5. FORMULATIONS OF pDNA/AZO-IAJD NANOCOMPLEXES

Formulations were prepared using the nanocomplexation method, in which a nucleic acid (NA) phase and a vector phase are mixed at a 9:1 ratio, respectively. The amount of each vector used in the formulation was calculated based on the desired DNA concentration, the N/P ratio, the vector's molecular weight, and the number of protonatable nitrogen atoms in the corresponding cationic derivative. For *E* isomers, stock solutions were maintained at 37 °C overnight, whereas *Z*-isomer stock solutions were generated by irradiating the vector solution at 365 nm for 2 h using an LC8 Lightningcure Hg/Xe lamp (200 W) equipped with a narrow-band interference filter (Hamamatsu A9616-07).

For the NA phase, pDNA (pCMV-Luc VR1216) was used for all characterization, EMSA, cytotoxicity, and *in vitro* and *in vivo* studies. The pDNA solution was prepared by diluting a stock solution in HEPES buffer (10 mM, pH 7.4, containing 5% glucose) to a final concentration of 15.60 µM. The required amount of vector was diluted from a 2 mM stock solution in DMSO. Subsequently, 900 µL of the pDNA solution was transferred to an Eppendorf tube, and 100 µL of the vector solution was added. The resulting mixture (final DMSO content: 10%) was homogenized, and the nanocomplexes were incubated for 30 min in the dark prior to characterization for *E* isomers, or analyzed immediately in the case of *Z* isomers.

Table S3. Variable parameters for the preparation of the different pDNA–photoswitchable nanocomplex formulations used in this study. Values are calculated to obtain 1000 µL of the final solution of the nanocomplexes upon mixing 900 µL of the pDNA phase and 100 µL of the vector phase in DMSO.

**Vector**[a]	**N/P**	**No. cationizable groups**[b]	**[Cationizable groups]$_{phase}$ [mM]**[c]	**Vol$_{Vector\ stock}$ [µL]**[d]
**1-3**	5	3	6	15.6
	10			31.2
	20			62.4
**4**	5	9	18	5.2
	10			10.4
	20			20.8

[a]Values are identical for formulations using the *E* or *Z* isomer. [b]Number of cationizable amino groups per molecule of the vector. [c]Desired concentration of cationizable amino groups in the final vector phase to get the target N/P. [d]Volume of vector stock solution in DMSO, adjusted with DMSO to get 100 µL of the vector phase

6. CHARACTERIZATION OF NANOPARTICLES

DLS/SLS measurements for each formulation were performed using a Stunner nanoparticle analysis system (Unchained Labs). Samples (2 µL) were loaded into 96-well Stunner plates, and all measurements were carried out in quadruplicate. DLS analysis was conducted with a buffer viscosity of 1.002 cP at 20 °C, using four acquisitions of 5 s each. Hydrodynamic size is reported as the mean diameter (± SD) of the major population based on the volume distribution.

ζ-Potential, which reflects the electrostatic potential at the interface between the particle surface and the bulk medium, was measured using a Malvern Zetasizer Nano ZS equipped with "mixed-mode measurement" phase analysis light scattering (M3-PALS). M3 combines slow and fast field-reversal modes to enhance accuracy and resolution. Measurement conditions were as follows: automatic sampling time; 12 measurements per sample; medium viscosity 1.002 cP; dielectric constant 80; temperature 25 °C.

7. EFFECT OF ALTERNATING IRRADIATION IN NANOCOMPLEX TOPOLOGY

To address whether photoisomerization can occur once compounds **6-9** are already complexed with pDNA, and whether this affects morphology, we performed two complementary sets of experiments, where the formulations were subjected to four alternating 5-min irradiations (two complete cycles) at 365 nm/466 nm (for *E* isomers) or 466 nm/365 nm (for *Z* isomers):

(a) We simultaneously recorded DLS and UV–Vis absorption data of the nanocomplexes before and after each irradiation step using a Stunner instrument (Unchained Labs). These measurements demonstrate efficient and reversible *E*/*Z* isomerization at every irradiation cycle, with moderate changes in hydrodynamic size or polydispersity index.

(b) Moreover, in a separate experiment, we monitored the average hydrodynamic diameter and ζ-potential using a Zetasizer Nano (Malvern) before irradiation and immediately after completion of the irradiation cycles.

The corresponding data are collected in Table S4.

Table S4. Average hydrodynamic diameter (D_h), polydispersity index (PDI), and ζ-potential values for nanocomplexes formulated with pCMV-LucVR1216 pDNA and Azo-IAJDs **1-4** in 9:1 water/DMSO before and after four irradiation cycles of 5 min each using a UV-vis lamp with alternating 365/466 nm (*E*-isomers) or 466/365 nm (*Z*-isomers) irradiation.

Compound	N/P	D_h (nm)	PDI	ζ-potential (mV)
**1**-*E*	10	104±1	0.270±0.05	+25.3±1.1
**1**-*E* (irradiated)	10	127±3	0.289±0.013	+19.6±0.9
**1**-*Z*	10	77±1	0.199±0.002	+23.1±1.9
**1**-*Z* (irradiated)	10	112±2	0.238±0.078	+17.8±2.2
**1**-*E*	20	81±1	0.132±0.026	+29.6±0.2
**1**-*E* (irradiated)	20	122±2	0.258±0.034	+20.4±0.4
**1**-*Z*	20	72±2	0.254±0.001	+22.6±3.3
**1**-*Z* (irradiated)	20	116±4	0.281±0.018	+16.0±1.8
**2**-*E*	10	92±1	0.164±0.001	+26.1±0.7
**2**-*E* (irradiated)	10	109±2	0.207±0.068	+18.3±1.3
**2**-*Z*	10	79±1	0.244±0.057	+20.9±0.2
**2**-*Z* (irradiated)	10	104±1	0.285±0.024	+16.0±1.5
**2**-*E*	20	87±1	0.152±0.013	+31.5±1.6
**2**-*E* (irradiated)	20	110±3	0.256±0.042	+23.1±2.4
**2**-*Z*	20	73 ± 2	0.250 ± 0.002	+25.9 ± 0.7
**2**-*Z* (irradiated)	20	100±2	0.291±0.035	+17.2±2.0
**3**-*E*	10	142±3	0.348±0.019	+21.0±0.6
**3**-*E* (irradiated)	10	173±4	0.487±0.023	+14.9±0.6
**3**-*Z*	10	125±1.	0.148±0.091	+22.1±0.6
**3**-*Z* (irradiated)	10	145±4	0.261±0.03	+15.1±1.2
**3**-*E*	20	112±1	0.163±0.005	+37.8±1.4
**3**-*E* (irradiated)	20	139±5	0.335±0.083	+23.2±0.7
**3**-*Z*	20	110±1	0.109±0.004	+31.3±3.6
**3**-*Z* (irradiated)	20	139±3	0.248±0.023	+20.5±0.9
**4**-*E*	10	97±1	0.166±0.004	+23.0 ± 1.0
**4**-*E* (Irradiated)	10	126±3	0.313±0.038	+18.5±1.4
**4**-*Z*	10	86±1	0.187±0.049	+25.0±1.7
**4**-*Z* (Irradiated)	10	109±2	0.213±0.036	+18.9±1.1
**4**-*E*	20	85 ± 2	0.316 ± 0.002	+26.5 ± 1.3
**4**-*E* (Irradiated)	20	122±4	0.330±0.017	+19.1±1.5
**4**-*Z*	20	74 ± 1	0.197 ± 0.035	+22.5 ± 3.6
**4**-*Z* (Irradiated)	20	103±1	0.239±0.027	+16.7±2.0

8. ELECTROPHORESIS MOBILITY SHIFT ASSAY (EMSA) GELS

Each vector:pDNA nanocomplex (25 μL of the formulation with 5 μL of 6X DNA Loading Dye; ThermoFisher Scientific) was submitted to electrophoresis using Mini-Sub Cell GT Cell and PowerPac™ Basic Power Supply (Bio-Rad, Hercules, CA, USA) for 40 min under 80 V through a 1.0% agarose gel (with 10 μL of 10000X GelRed Nucleic Acid Stain, Merck) in 100 mL of TAE buffer 1X (Tris-acetate and EDTA). For protection assays, DNase I (1U/μg pDNA) was added to each sample and incubated for 30 min at 37 ºC. 1 μL of 50 mM EDTA was added to inactivate DNAase and the sample was vortexed and incubated for 10 min at rt. Then, 2.5 μL of 15% (w/v) aq sodium dodecyl sulfate (SDS) was then added and further incubated for 10 min at rt. Samples were electrophoresed as described above. Plasmid integrity was compared with free pDNA both treated and untreated. The DNA was then visualized after photographing on a Gel Doc EZ Imager with an UV Sample Tray (Bio-Rad, Hercules, CA, USA).

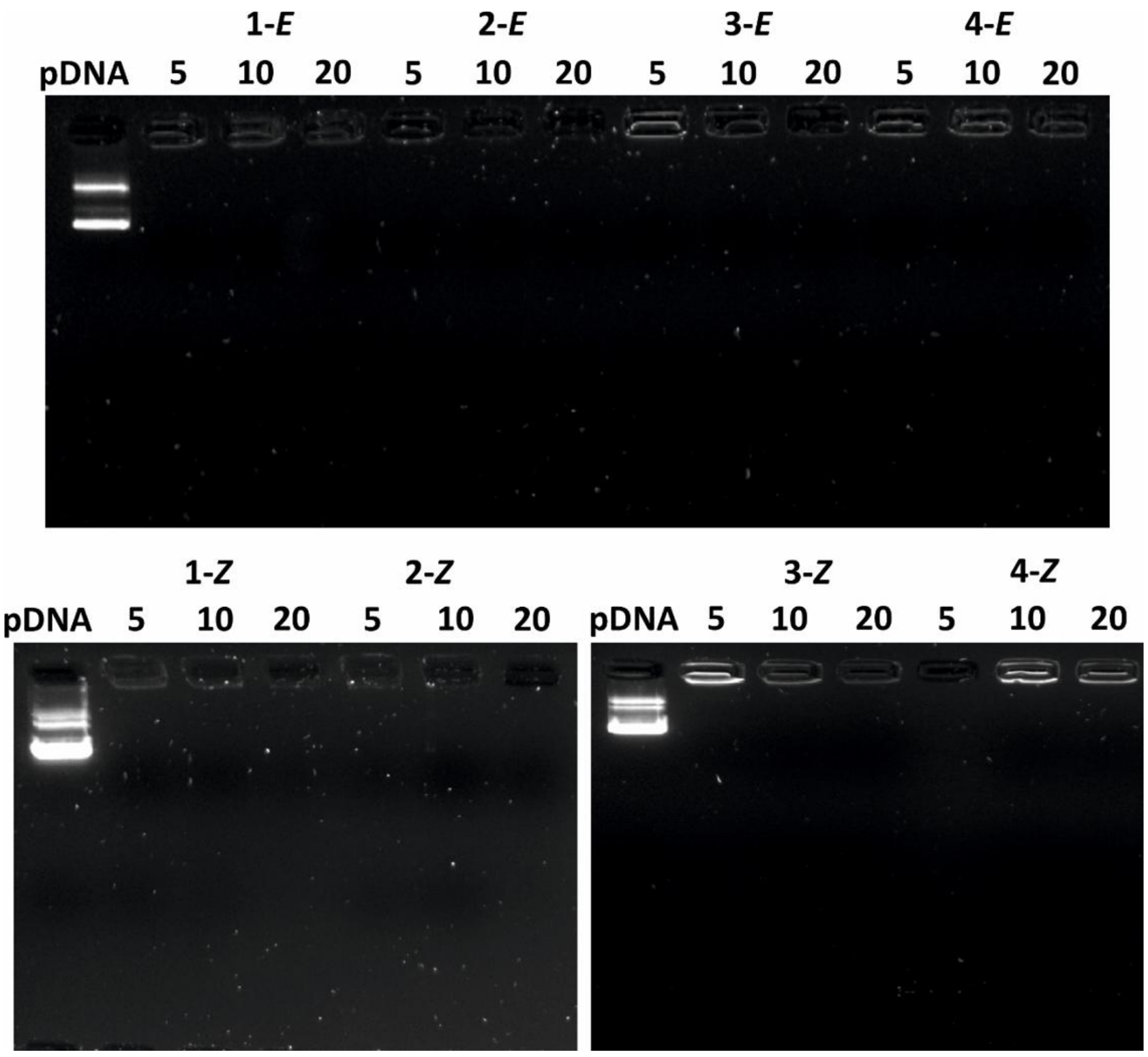

Figure S31. EMSA gels for nanocomplexes formulated with pDNA (pCMV-Luc VR1216) and both the *E*- and *Z*-isomers of **1-4** at N/P 5, 10 and 20; naked pDNA was used as a control.

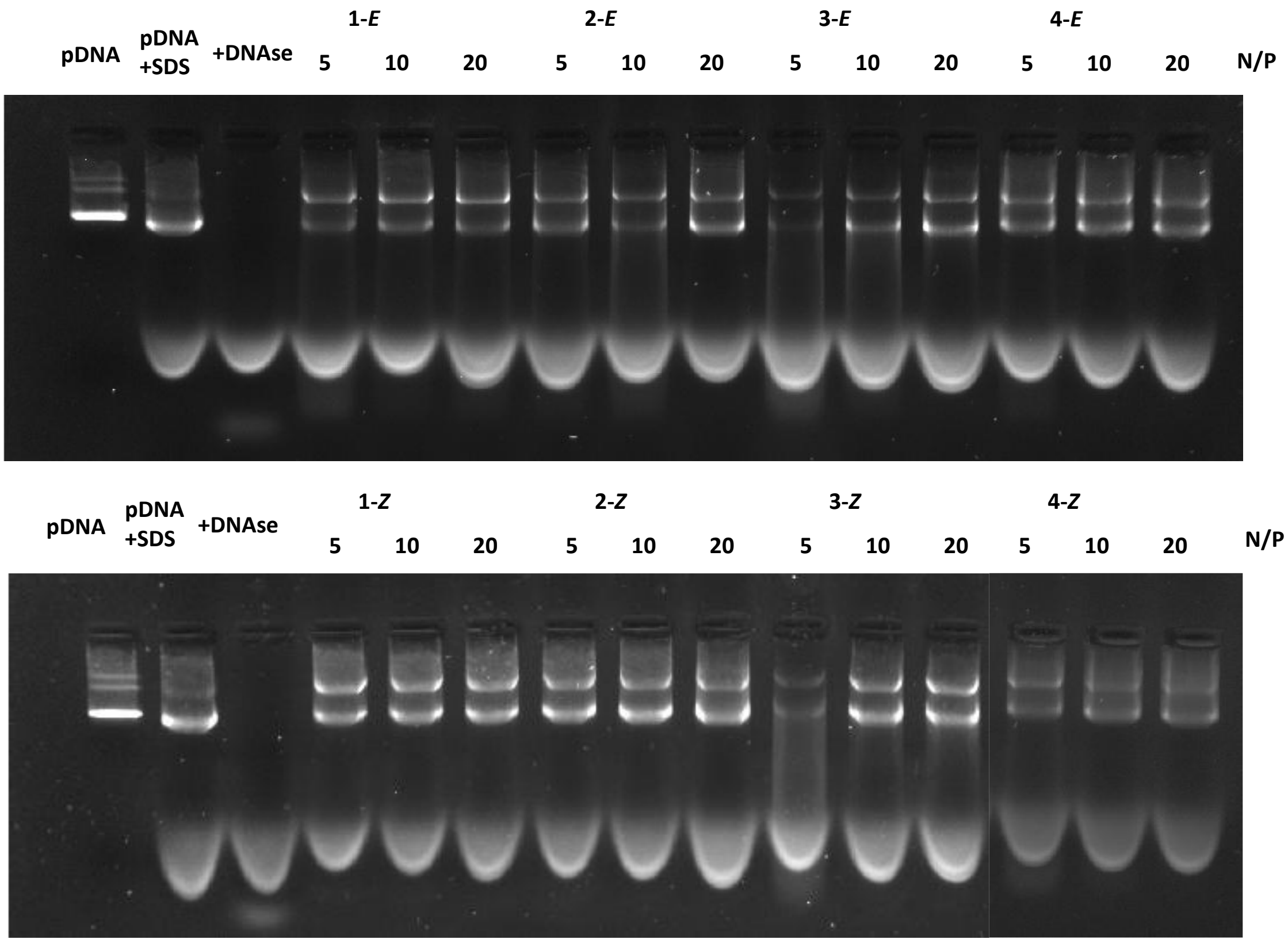

Figure S32. EMSA gels for nanocomplexes formulated with pDNA (pCMV-Luc VR1216) and both the *E*- and *Z*-isomers of **1-4** at N/P 5, 10 and 20; after treatment with DNase I and subsequent dissociation of the complexes with sodium dodecyl sulfate; naked pDNA was used as a control.

9. TRANSMISSION ELECTRON MICROSCOPY (TEM)

Formvar-carbon coated grids previously made hydrophilic by glow discharge were placed on top of small drops of the nanocomplexes prepared as describe above using N/P 10 ratios. After 1-3 min, grids were negatively stained with a few drops of 1% or 2% aqueous solution of uranyl acetate. The grids were then dried and observed with a Thermo Fisher TALOS L120C electron microscope operated at 120 kV or a Philips CM12 electron microscope working under standard conditions. Images were taken under low dose conditions with a Thermo Fisher CETA-F camera. All the experiments were reproduced twice on each formulation.

10. CELL VIABILITY ASSAY

Cell viability in COS-7, HepG2, and RAW264.7 cells (Table S5 and Figures S33 and S34) was assessed using a modified AlamarBlue® assay (Invitrogen). Briefly, 48 h after transfection, 1 mL of AlamarBlue reagent diluted to 10% (v/v) in complete medium was added to each well. Following a 2.5-h incubation at 37 °C, 200 µL of the supernatant was transferred to a 96-well plate, and absorbance was measured at 570 and 600 nm. Cell viability (expressed as a percentage of control cells) was calculated using the formula:

Viability = (A_{570}–A_{600}) of treated cells × 100/(A_{570}–A_{600}) of control cells.

Table S5. Cell viability in COS-7, HepG2 and RAW264.7 cells treated with pDNA nanocomplexes.

		N/P	**1**	**2**	**3**	**4**	**PEI**
E-isomer	COS-7	10	98.7 ± 3.2	100.3 ± 6.4	98.0 ± 5.0	101.5 ± 2.8	94.0 ± 4.8
		20	91.3 ± 4.2	94.2 ± 7.2	92.8 ± 5.8	95.7 ± 3.9	
	HepG2	10	89.7 ± 3.9	100.1 ± 6.2	92.9 ± 1.8	97.2 ± 5.6	87.8 ± 4.6
		20	85.7 ± 3.2	90.2 ±5.7	86.9 ± 3.0	88.3 ± 4.2	
	RAW264.7	10	88.6 ± 7.4	99.2 ± 5.0	90.4 ± 5.2	98.7 ± 3.8	68.8 ± 4.5
		20	81.4 ± 2.9	95.3 ± 5.4	87.9 ± 3.3	93.1 ± 4.8	
Z-isomer	COS-7	10	97.8 ± 5.1	100.0 ± 2.6	99.0 ± 4.9	101.6 ± 5.8	94.0 ± 4.7
		20	87.5 ± 2.7	96.1 ± 4.5	90.8 ± 1.4	95.7 ± 3.2	
	HepG2	10	92.3 ± 4.2	94.2 ± 1.7	93.1 ± 5.0	90.6 ± 3.5	87.8 ± 4.6
		20	90.1 ± 6.2	92.4 ± 4.0	89.5 ± 1.7	89.6 ± 5.1	
	RAW264.7	10	90.5 ± 5.4	101.9 ± 4.7	92.5 ± 6.6	101.0 ± 3.8	68.8 ± 4.5
		20	85.1 ± 3.3	97.5 ± 5.7	85.2 ± 4.1	96.7 ± 6.3	

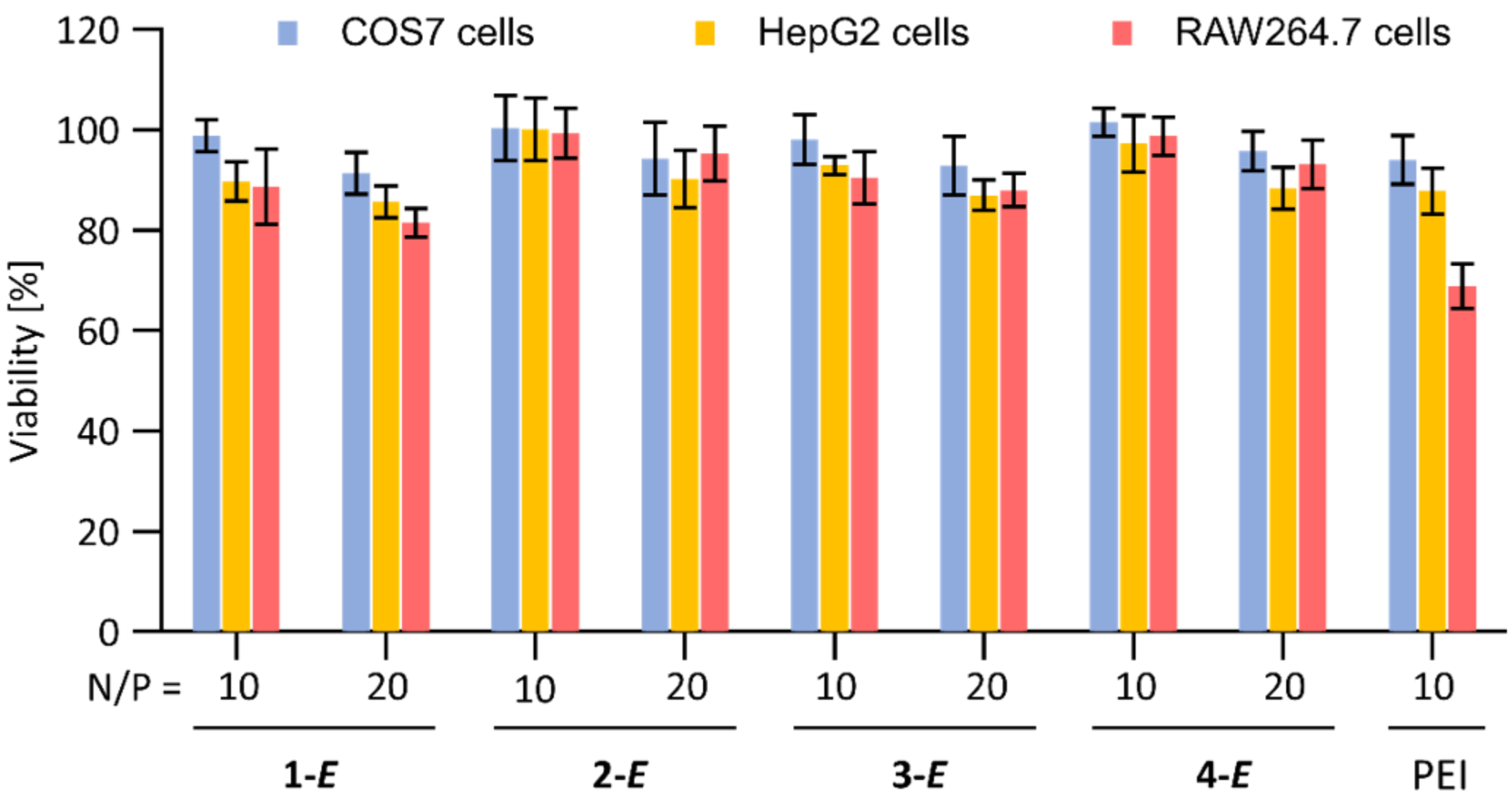

Figure S33. Cell viability in COS-7, HepG2 and RAW264.7 cells treated with *E*-isomer pDNA nanocomplexes.

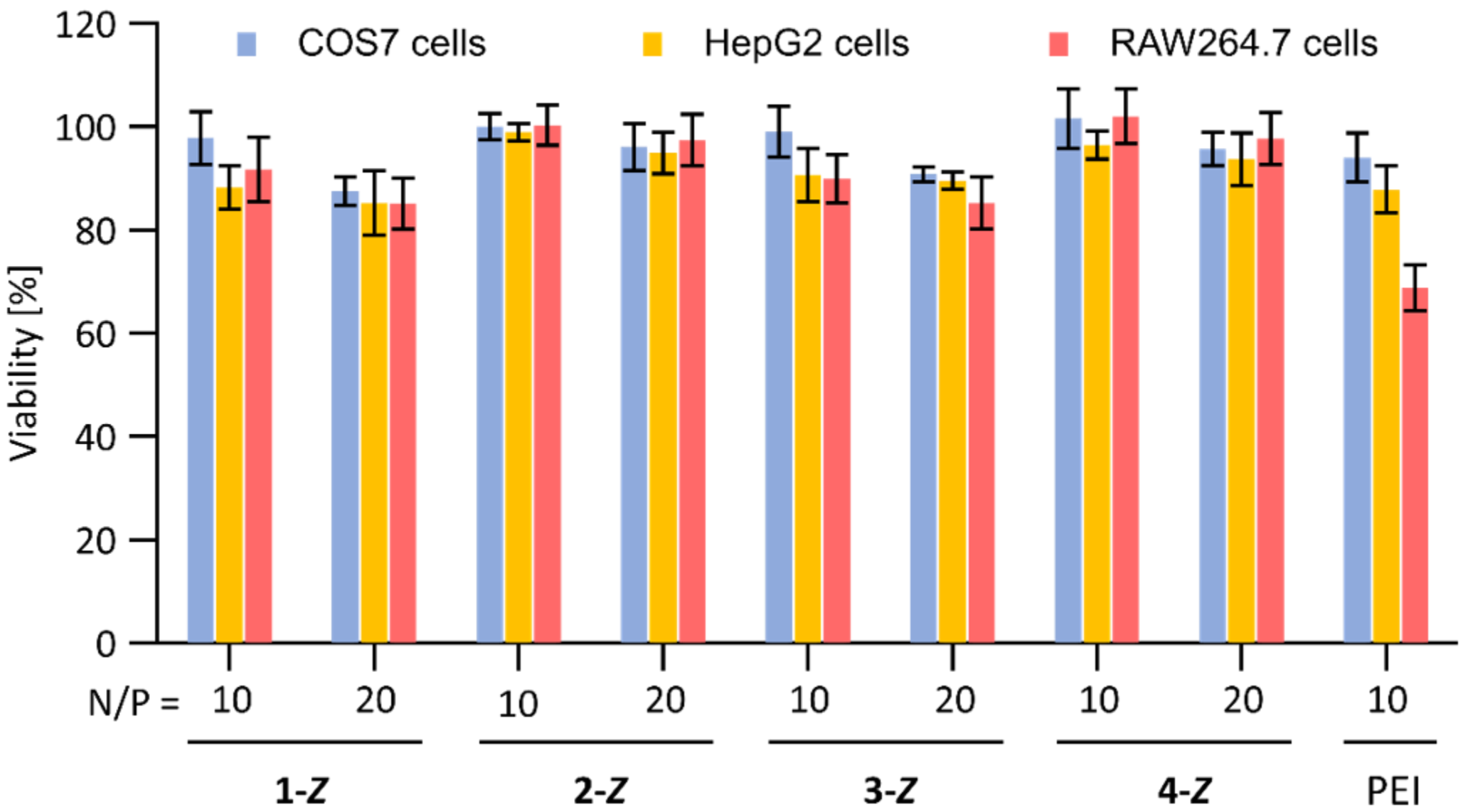

Figure S34. Cell viability in COS-7, HepG2 and RAW264.7 cells treated with *Z*-isomer pDNA nanocomplexes.

11. *IN VITRO* GENE TRANSFECTION ASSAYS

Cells (COS-7, HepG2 or RAW264.7) were seeded in medium at a density of 6000 cells per well in 48-well plates (Iwaki Microplate, Japan) and incubated for 24 h at 37 ºC in 5 % CO_2. The culture plates for each cell line were divided into three groups (I-III). The medium was removed in the three groups, and 0.3 mL of complete medium (activated 10% FBS), 0.2 mL of nanocomplexes formulated with the Azo-IAJDs **6-9** containing 1 μg of pDNA, were added to each well and the plates were incubated for 4 h at 37 ºC. 25 kDa PEI polyplexes (N/P 10), SuperFect® (SFect) dendriplexes (SFect/pDNA mass ratio 10:1) and Lipofectamine® 3000 (LP) lipoplexes (formulated prepared following the manufacturer's protocol) were sued as controls. Cells in group I were supplemented by fresh complete medium and further incubated for 48 h at 37 ºC in the dark. Cells for group II were irradiated for 15 min under an UV lamp at 365 or 466 nm (for *E*- or *Z*-isomer formulations, respectively), then supplemented by fresh complete medium and further incubated for another 47 h and 45 min in the dark. Cells in group III were subjected to four irradiation cycles of 15 min each under an UV lamp alternating 365/466 nm or 466/365 nm for *E*- and *Z*-isomer formulations, respectively), then supplemented by fresh complete medium and further incubated for another 47 h in the dark. The irradiation power at the 365 and 466 nm wavelengths are 5.4 and 20 $mW{\cdot}cm^{-2}$, respectively. Cells in all groups were washed with phosphate-buffered saline (PBS) and lysed with 100 μL of Reporter Lysis Buffer (Promega, Madison, WI) at rt. for 10 min, followed by a freeze-thaw cycle. A 20 μL aliquot of the supernatant was assayed for total luciferase activity by using the luciferase assay reagent (Promega), according to the manufacturer's protocol. A luminometer (Sirius-2, Berthold Detection Systems, Innogenetics, Diagnóstica y Terapéutica, Barcelona, Spain) was used to measure luciferase activity. The protein content of the lysates was measured by DC protein Assay Reagent (Bio-Rad, Hercules, CA) with bovine serum albumin as the standard. The data were expressed as picograms of luciferase (based on a standard curve for luciferase activity) per mg of protein. Samples were analyzed in a plate spectrophotometer Power Wave XS and a data processor KC junior, BioTek.

Table S6. *In vitro* cell transfection assay [pgLUC/mg protein] of Azo-IAJDs derivatives **6-9** (N/P 10) and pDNA (15.60 μM, pCMV-Luc VR1216), measured without irradiation, irradiated for 15 min under an UV-vis lamp at 365 or 466 nm (for *E*- or *Z*-isomer formulations, respectively) and after four irradiations of 15 min each under an UV-vis lamp alternating 365/466 nm or 466/365 nm (for *E*- and *Z*-isomer formulations, respectively).

			**1**	**2**	**3**	**4**
E isomer	COS-7	No irrad.	602 ± 20	4005 ± 68	47 ± 11	41588 ± 2296
	HepG2	No irrad.	318 ± 60	1138 ± 204	26 ± 8	2126 ± 174
	RAW264.7	No irrad.	9 ± 2	18 ± 2	3 ± 1	174 ± 11
Z isomer	COS-7	No irrad.	2831 ± 153	15057 ± 1003	71 ± 15	60720 ± 6820
	HepG2	No irrad.	1303 ± 159	4382 ± 622	60 ± 7	3550 ± 387
	RAW264.7	No irrad.	100 ± 13	74 ± 9	11 ± 4	498 ± 43

Table S7. *In vitro* cell transfection assay [pgLUC/mg protein] of Azo-IAJDs derivatives **1-4** (N/P 20) and pDNA (15.60 μM, pCMV-Luc VR1216), measured without irradiation, irradiated for 15 min under an UV-vis lamp at 365 or 466 nm (for *E*- or *Z*-isomer formulations, respectively) and after four irradiations of 15 min each under an UV-vis lamp alternating 365/466 nm or 466/365 nm (for *E*- and *Z*-isomer formulations, respectively).

			**1**	**2**	**3**	**4**
E isomer	COS-7	No irrad.	965 ± 86	8646 ± 359	374 ± 47	79860 ± 4593
		1 Irrad.	9772 ± 1061	51739 ± 3993	1163 ± 147	148459 ± 11327
		4 Irrad.	16060 ± 1477	63116 ± 6520	1570 ± 178	179684 ± 19022
	HepG2	No irrad.	731 ± 87	2279 ± 185	527 ± 64	3643 ± 274
		1 Irrad.	4455 ± 538	10061 ± 924	3208 ± 247	6913 ± 843
		4 Irrad.	5931 ± 651	13766 ± 1047	4130 ± 384	8015 ± 619
	RAW264.7	No irrad.	19 ± 3	55 ± 4	4 ± 1	428 ± 52
		1 Irrad.	796 ± 83	676 ± 42	60 ± 7	1359 ± 155
		4 Irrad.	1033 ± 147	838 ± 70	80 ± 9	1936 ± 252
Z isomer	COS-7	No irrad.	6734 ± 812	28272 ± 3412	636 ± 58	114199 ± 9465
		1 Irrad.	13975 ± 1476	84332 ± 7031	2244 ± 179	176666 ± 14377
		4 Irrad.	20075 ± 1875	92681 ± 8762	2637 ± 284	199449 ± 16931
	HepG2	No irrad.	2129 ± 166	5675 ± 658	1250 ± 80	5009 ± 614
		1 Irrad.	5620 ± 386	10612 ± 827	3799 ± 401	7112 ± 794
		4 Irrad.	7750 ± 549	17252 ± 1802	7386 ± 659	9075 ± 1077
	RAW264.7	No irrad.	222 ± 20	292 ± 32	18 ± 3	925 ± 80
		1 Irrad.	891 ± 95	837 ± 72	69 ± 8	1655 ± 137
		4 Irrad.	1092 ± 95	951 ± 101	94 ± 8	2734 ± 289

12. *IN VIVO* GENE TRANSFECTION ASSAYS

BALB/c mice (6–8 weeks old, 20–25 g) were obtained from Harlan Ibérica Laboratories. Groups of eight animals (four males and four females per group) were intravenously injected via the tail vein with 200 μL of nanocomplexes containing 50 μg of pCMV-Luc VR1216 plasmid DNA at an N/P ratio of 10. Naked DNA was administered as a control. Twenty-four hours after injection, the mice were euthanized, and the liver, heart, kidneys, lungs, and spleen were collected and rinsed with cold PBS. Each organ was homogenized in 1 mL of lysis buffer using a Mini-Beadbeater homogenizer (BioSpec Products, Bartlesville, OK) at 5000 rpm, followed by centrifugation at 10000 rpm for 3 min. A 20 μL aliquot of each supernatant was then analyzed for luciferase activity using the same procedure as in the *in vitro* assays. All animal procedures were conducted in accordance with established ethical guidelines, following European Directive 86/609/EEC and national regulations for the protection of experimental animals, and were approved by the Committee on Animal Research at the University of Navarra–CIMA (ID ES/31-2010-000132; accreditation number CEEA 026-24).

Table S8. *In vivo* cell transfection assay [pgLUC/mg protein] of Azo-IAJDs (N/P 10) and pDNA (15.60 μM, pCMV-Luc VR1216) in mice.

		**1**	**2**	**3**	**4**
E isomer	Heart	0.34 ± 0.1	11.8 ± 3.4	0.25 ± 0.10	18.6 ± 4.0
	Liver	12.4 ± 2.7	17.9 ± 5.3	6.3 ± 2.5	104.6 ± 14.0
	Lung	37.9 ± 7.1	68.3 ±14.8	21.5 ± 5.8	76.7 ± 8.5
	Spleen	1.1 ± 0.5	29.8 ± 7.6	0.96 ± 0.40	123.4 ± 16.1
	Kidney	3.6 ± 1.9	8.8 ±3.1	1.1 ± 0.4	68.8 ± 10.4
Z isomer	Heart	1.5 ± 0.5	43.7 ± 12.3	0.57 ±0.30	27.3 ± 4.2
	Liver	47.3 ± 12.0	92.6 ±18.2	25.7 ± 4.3	132.2 ± 11.0
	Lung	9.6 ± 2.8	76.0 ± 8.8	14.1 ± 3.4	68.3 ± 9.2
	Spleen	6.4 ± 2.5	54.6 ± 12.0	9.9 ± 3.1	113.0 ± 12.8
	Kidney	3.0 ± 1.0	37.1 ± 9.3	1.8 ± 0.9	82.8 ± 15.2

13. STATISTICAL ANALYSIS

Statistical analyses were performed using SPSS software from SPSS Inc. (Chicago, IL). The analysis of the transfection efficiency of the nanocomplexes was performed with a two-tailed unpaired Student's t-test. $P < 0.05$ was considered statistically significant.

14. MOLECULAR MECHANICS (MM) AND MOLECULAR DYNAMICS (MD)

Computational Methods.

Molecular Mechanics (MM) and Molecular Dynamics (MD) simulations were performed, initially, to study the stability of ordered bilayers of Azo-IAJDs and the intermolecular forces governing their formation. Compound **6** was selected for this purpose. This derivative possesses three ionizable primary amines on one side and three hydrophobic octyl tails on the opposite side, in a well-defined Janus-type arrangement. It can exist in either the *E* or *Z* configuration. The interactions between these ordered *E* or *Z* bilayer structures and two B-DNA helical fragments were further investigated within a periodic box of explicit water. Moreover, partial configurational transitions of selected azobenzene groups (*E*→*Z* or *Z*→*E*) within the bilayers were examined to emulate the effects of photoisomerization.

The Sybyl X-2.0 (SYBYL-X 2.0; Tripos Associates; St. Louis, MO, 2012) and the Tripos Force Field were used for all calculations.[6] B-DNA fragments contained twelve nucleotides with a CGCGAATTCGCG sequence each. Charges for the overall neutral hydrochloride salt of **6** were obtained by MOPAC (AM1) using the Gaussian program.[7] The Molecular Silverware algorithm (MS) and periodic boundary conditions (PBC) were used for solvation.[8] A relative permittivity $\varepsilon=1$ was employed for electrostatic contributions in the presence of explicit water. Non-bonded cut-off distances for MM, as well as for MD were set at 12 or 8 Å. Optimizations were carried out by the simplex algorithm, and the conjugate gradient was used as a termination method with gradients of 1.5 Kcal/molÅ for the MM calculations.[9,10]

MD simulations were performed following the previously described strategy.[11,12,13,14] In brief, the simulations were initiated at 1 K, and the temperature was gradually increased in 20 K increments, with the system equilibrated for 500 fs at each intermediate temperature until the target temperature of 300 K was reached. Once at 300 K, an additional equilibration period of up to 25 ps was applied. The entire heating and equilibration phase was excluded from the subsequent analysis. Following equilibration, the production simulations were performed using a 2 fs integration time step, with velocities rescaled every 10 fs. Structures were saved every 2000 fs for further analysis of the MD trajectories.

To ensure that the N···Cl lengths of virtual bonds in neutral Azo-IAJD **6** systems remained near their minimum energy length, a harmonic potential energy function was incorporated into the force field. This function is defined as $E = k(l-l_e)^2$, where k = 200 kcal/molÅ², l_e represents the equilibrium favorable N···Cl distance, and *l* denotes the distance between the N and Cl atoms. In a similar manner, in case of supramolecular DNA assemblies, in order to maintain a regular helical structure for the pair of DNA chains and to avoid the fraying of the end portions of the short DNA helix during MD, the N···HN hydrogen bond distances for each pair of DNA complementary bases were constrained to keep them constant. For this purpose, (as previously for the virtual N ···Cl virtual bonds) a harmonic penalty function was added to the force field for those atoms which were involved in the constraint. This energy function is written as $E = k\ (r-r_i)^2$, where k = 200 kcal/molÅ^2, r_i and *r* are the initial equilibrium distance and the variable distance during the MD trajectory, respectively.

Modelling bilayers of Azo-IAJD 1.

Three-dimensional bilayers of **1** with the azobenzene groups in either the *E* or *Z* configuration were built through a stepwise process consisting of:

*(a) Supramolecular homodimer formation by interaction of two chloride neutralized units of **6** through their hydrophobic tails.* Initially, two neutral, optimized, and stable structures of **1**, in either the *E* or *Z* configuration, were brought together along the *y*-coordinate. For this purpose, one unit of **1** (*E* or *Z*) was fixed at the origin (*o*) of a coordinate system, as depicted in Figure S35, while a second unit in the same isomeric state was approached in 0.5 Å increments along the y-axis from y = +5.5 Å to +2.5 nm in the presence of water. The first dimer structure, generated with the two units of **1** separated at the maximum distance (*o*–*o*') where they interacted minimally (5.5 nm), was solvated (MS and PBC) and subjected to a single optimization (gradient threshold: 1.5 kcal/mol·Å). The solvent box was then removed, the *o*–*o*' distance was reduced by 0.05 nm, and the structure was again solvated and optimized. This iterative process continued, with each optimized structure serving as the starting point for the subsequent step.

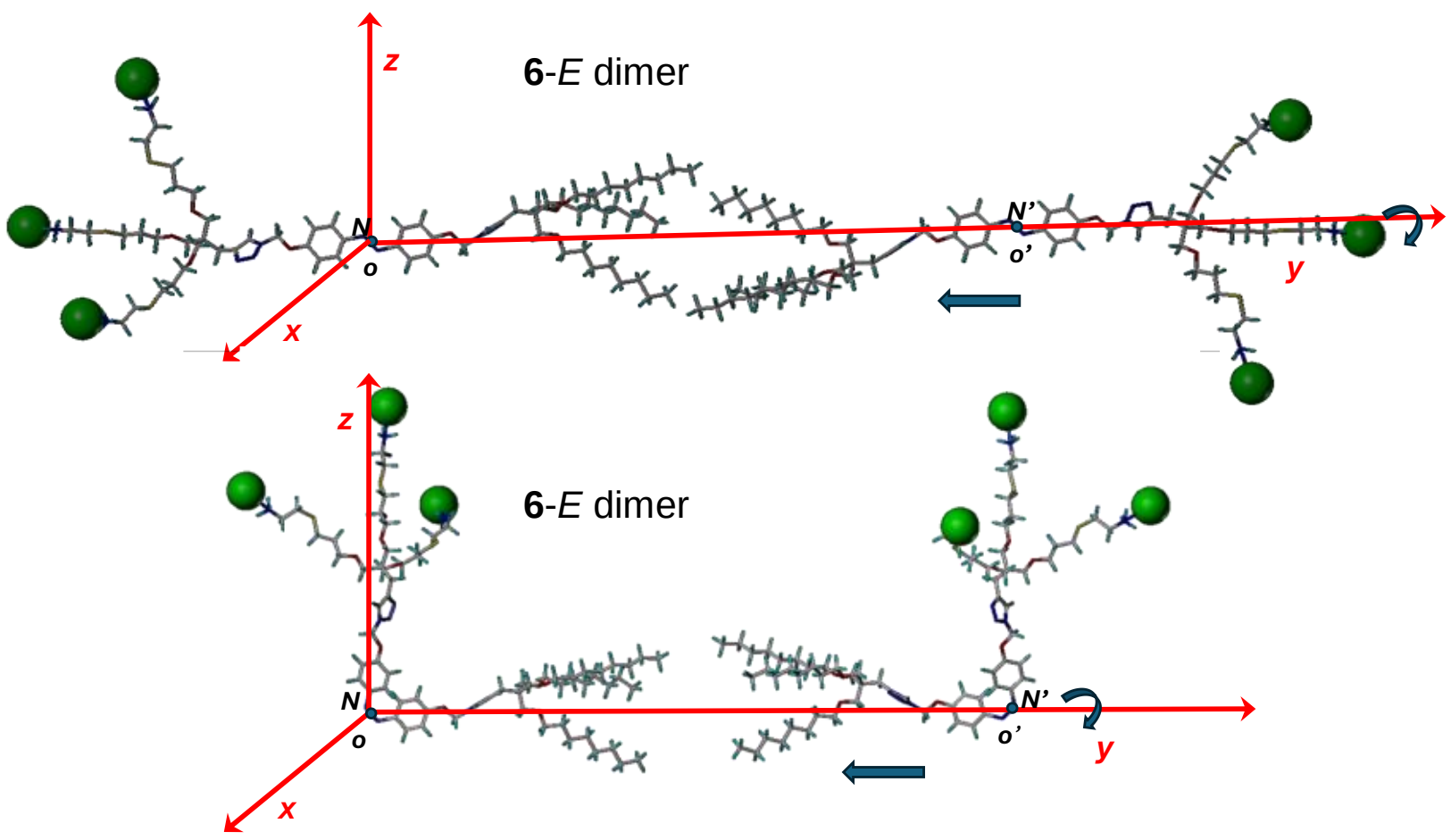

Figure S35. Coordinate system used for **1–1** approaching along the y coordinate to form a homodimer with both azobenzene derivatives in the *E* or *Z* configuration.

Prior to approaching the two units, the most favourable relative orientation of the two units of **1** (measured by the N–*o*–*o*'–N' dihedral angle, θ) was estimated. This was achieved by calculating the **1–1** interaction energy while varying the *o*–*o*' distance along the y-axis (from y = +6.0 nm to +3.0 nm in 0.1 nm increments) and the θ angle in small steps of 10 deg from –180 deg to +180 deg. Figure S37 shows the total binding energy, along with the contributions from electrostatic and van der Waals interactions, as a function of the *o*–*o*' distance obtained during the **1**-to-**1** approaching at the most favourable orientations. The total binding energies were favourable and decreased with decreasing distance, reaching a minimum binding energy (MBE) for the dimer, formally a 1 × 1 bilayer structure ($MBE_{1\times1}$). The largest contributions to the binding energy were from van der Waals interactions. Figure S36 also depicts the resulting MBE **1**-*E*- and **1** *Z* dimer structures.

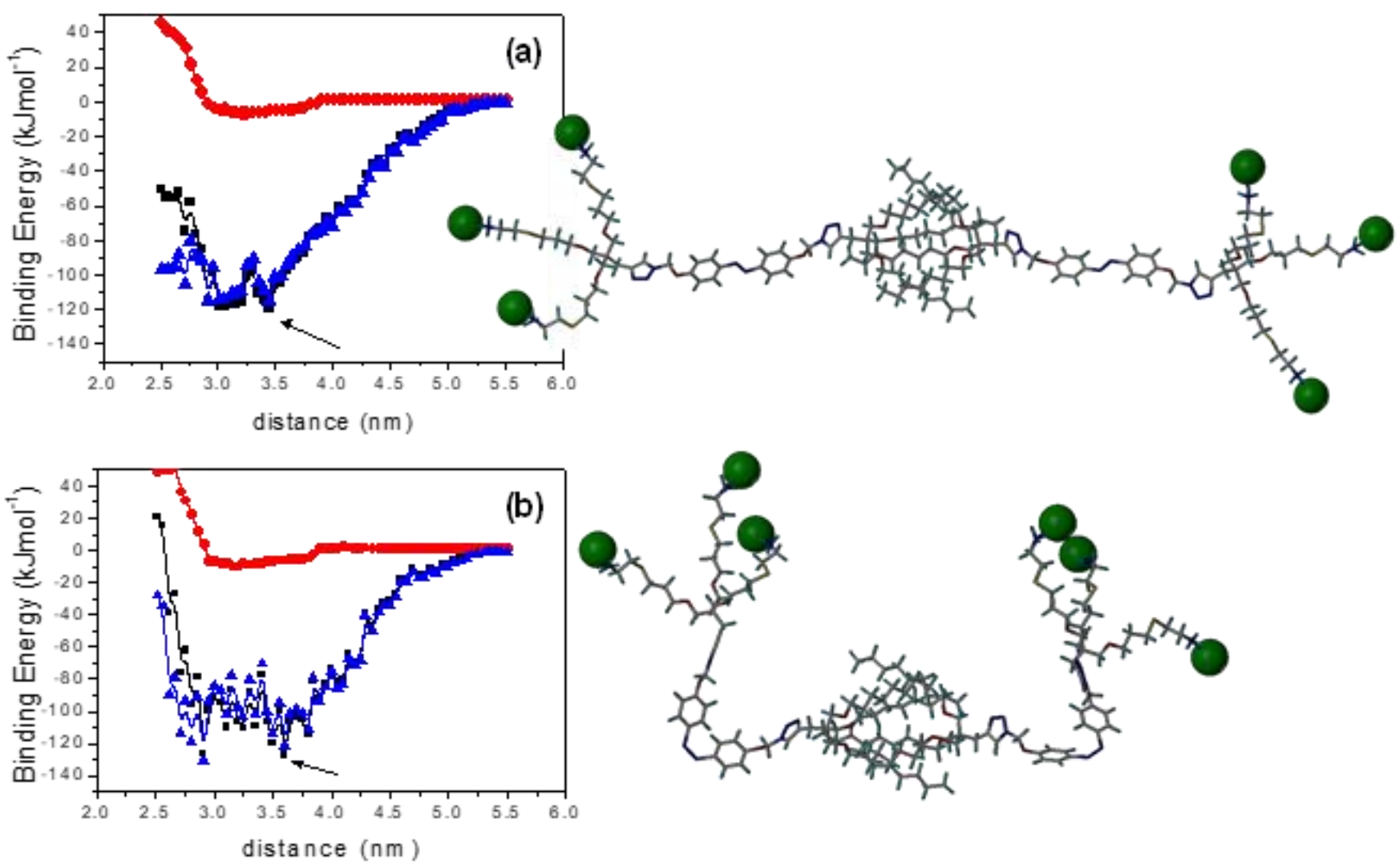

Figure S36. Total binding energy (black squares) and contributions from electrostatics (red circles) and van der Waals interactions (blue triangles) during the stepwise approach of two units of **1** in the *E* (top panel) or *Z* (bottom panel) configurations along the y-axis, with optimized relative orientations. The distance is measured between o and o'. This distance will subsequently be used to evaluate the order and stability of a three-dimensional bilayer. The minimum binding energy ($MBE_{1\times1}$) dimer structure is superimposed and indicated by an arrow.

*(b) Side by side interaction of homodimers to construct bidimensional bilayers of **6** (all-E or all-Z).* Two $MBE_{1\times1}$ homodimer structures of **1** (labelled 2 and 3 in Figure S37) were approached to a third reference dimer (labelled 1 in Figure S37) in 0.05 nm steps along the *z* coordinate from *x* = +2.5 to +0.3 (nm) (or *x* = –2.5 to –0.3 nm) in the presence of explicit water, following the same protocol as for dimer formation. Figure S38A and B shows the binding energy and electrostatics and van der Waals contributions as a function of the *o*(1)-*o*(2) and *o*(1)-*o*(3) distances obtained during the approaching process of *E* and *Z* homodimers, respectively, in the most favorable orientations, as depicted in Figure S37. The binding energies were negative and decreased with decreasing distance, reaching a minimum binding energy ($MBE_{1\times3}$) structure. This $MBE_{1\times3}$ structure corresponds to the point where both the total energy and the strain energy (sum of bond stretching, angle bending, and torsional components) begin to increase due to dimer–dimer interactions (not shown). Figure S38A and B also depicts the $MBE_{1\times3}$ structures for both the all-*E* and all-*Z* 1 × 3 bilayers, indicated by arrows in the graphs after energy minimization. The inter-dimer distances in the minimized bidimensional 1 × 3 bilayers were 0.67 and 0.51 nm for the all-*E* and 0.59 and 0.58 nm for the all-*Z* configuration. These optimized structures were subsequently used to construct the three-dimensional 3 × 3 bilayers.

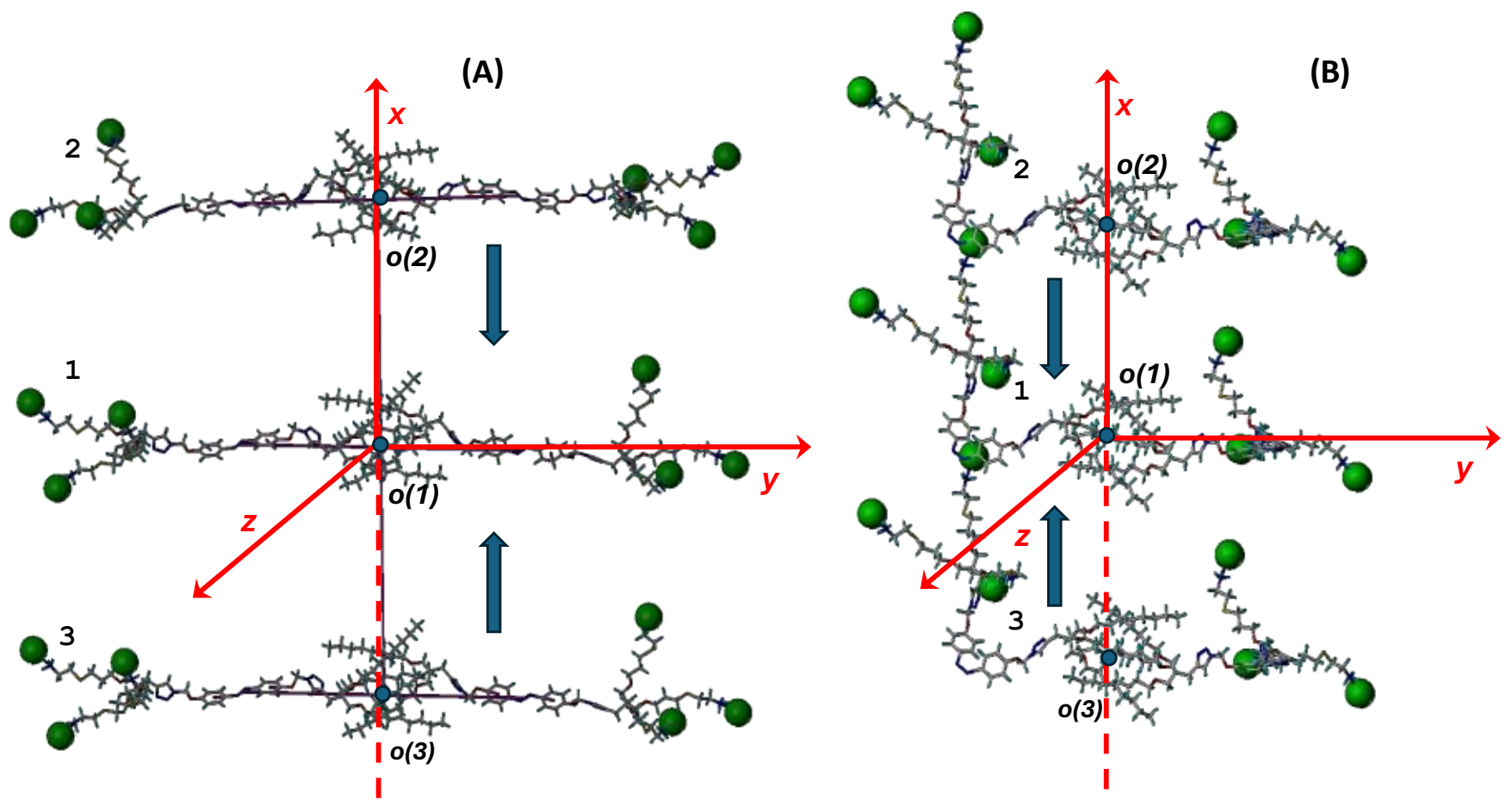

Figure S37. Coordinate system used for the approaching two homodimers of **1** (labelled 2 and 3), in the *E* (A) or *Z* (B) configuration, to a reference dimer (labelled 1) laterally along the *x* coordinate.

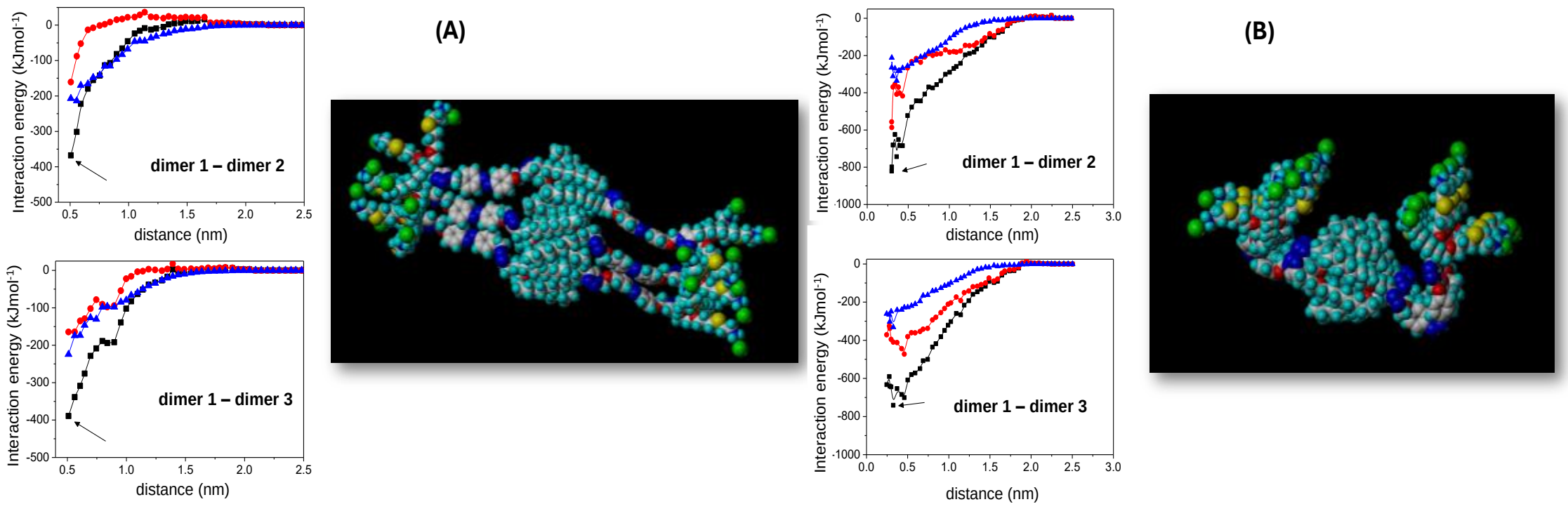

Figure S38. Total interaction energy (black squares) and electrostatics (red circle) and van der Waals (blue triangle) contributions upon two homodimers of **1** (labelled 2 and 3) approaching a reference dimer (labelled 1) along the *x*-axis in the optimized orientation. Distances are measured as *o*(2)–*o*(1) (dimer 1–dimer 2) and *o*(3)–*o*(1) (dimer 1–dimer 3). The $MBE_{1\times3}$ structures for the *E* (A) and *Z* (B) configurations are indicated by the arrows.

(c) Lateral interaction of bidimensional 1 × 3 bilayers to construct three-dimensional bilayers of ***1*** *(all-E or all-Z).* Two all-*E* or all-*Z* 1 × 3 bidimensional bilayers, corresponding to the $MBE_{1\times3}$ structures shown in Figure S37, were simultaneously approached along the *z*-axis, one (labelled as 3) from the positive direction and the other (labelled as 2) from the negative direction, toward a reference $MBE_{1\times3}$ structure (numbered as 1) centred at the origin of the coordinate system *o*(1), as illustrated in Figure S39. The approach was performed from z = +2.5 nm to +0.3 nm (and from z = –2.5 nm to –0.5 nm) in increments of 0.025 nm. At each step, various energy contributions were calculated as a function of the separation between the bilayers, measured as the *o(1)–o(2)* and *o(1)–o(3)* distances (Figure S40). The total interaction energy becomes increasingly favourable as the bilayers approach one another. The structure indicated by the arrow marks the point at which the interaction energy reaches its minimum. At distances shorter than this point, the total energy (not shown) becomes less favourable, primarily due to increased lateral repulsive interactions between the 3 × 3 bilayers and the strain energy accumulated within each bilayer. Three-dimensional 3 × 3 bilayer all-*E* or all-*Z* $MBE_{3\times3}$ assemblies, after removal of chloride atoms and further geometry optimization, were used to simulate the interactions with DNA fragments via their charged domains as described in the next section.

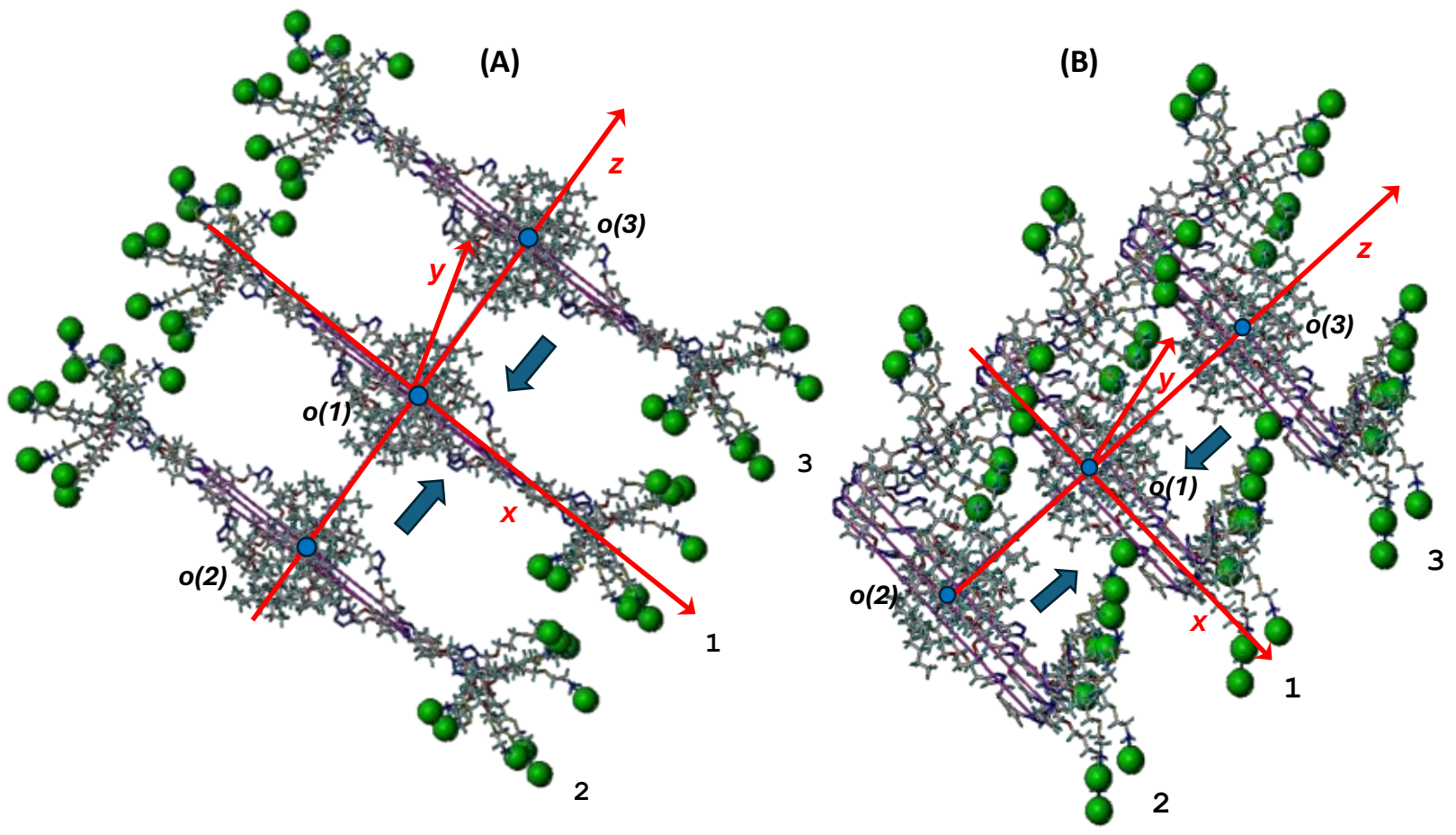

Figure S39. Coordinate system used for the simultaneous approach of two bidimensional *all-E*(A) and *all-Z* (B) 1 × 3 bilayers (labelled 2 and 3) of **1** toward a central bilayer (labelled 1) along the *z*-axis.

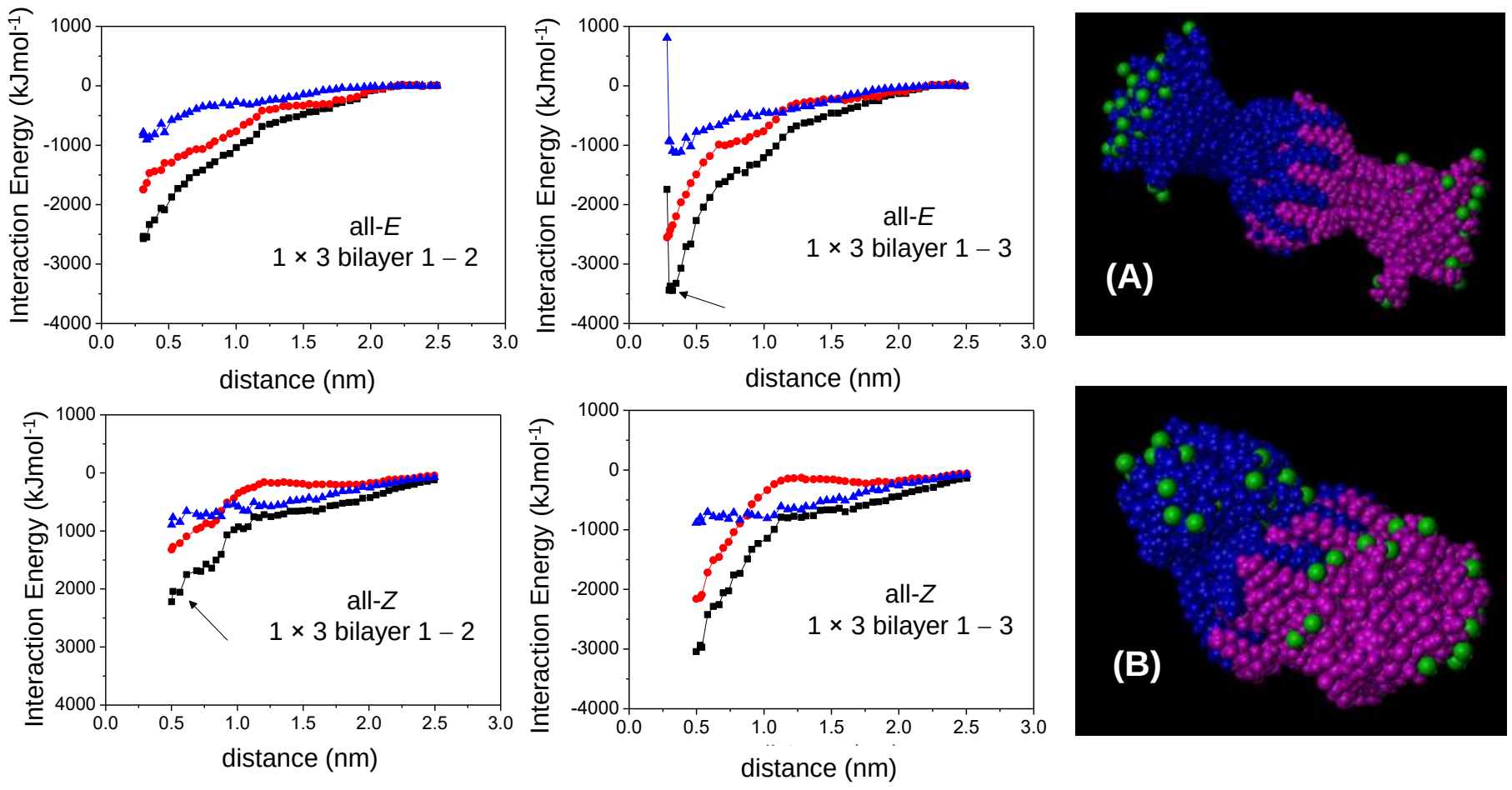

Figure S40. Total interaction energy (black squares) and its electrostatic (red circles) and van der Waals (blue triangles) contributions for bilayer pairs 1–2 and 1–3 during the approach of two bidimensional 1 × 3 bilayers (labelled 2 and 3) toward a central reference bilayer (labelled 1), as depicted in Figure S38, to form a three-dimensional 3 × 3 bilayer. The upper panel (A) correspond to the *all-E*-configuration, and the lower panel (B) the *all-Z*-configuration. The corresponding $MBE_{3\times3}$ bilayer structures, indicated by the arrows, are depicted.

Modelling the interactions of 3 × 3 bilayers of cationized Azo-IAJD 1 with DNA fragments.

One of the objectives of this work was to investigate the stability of the supramolecular assembly formed by cationized 3 × 3 bilayers of **1**, in the *all-E* and *all-*Z configuration, and two DNA fragments by MM and MD. Initially, the corresponding $MBE_{3\times3}$ bilayer was positioned with the centre of mass of its hydrophobic chains at the origin of a coordinate system, and the DNA fragments were initially placed at distances where their interaction with the bilayer structure was negligible (Figure S41). For the system in the *all-E* configuration (Figure S40A), both DNA1 and

DNA2 fragments were simultaneously moved toward the cationic side along the *x*-axis in 0.05 nm increments. Specifically, DNA1 was translated from *x* = +5.5 to +3.0 nm, and DNA2 from *x* = −5.5 to −3.0 nm, approaching the 3 × 3 bilayer via the major groove. For the *all-Z* configuration (Figure S40B), the approaching was carried out similarly, but along two virtual axes (*x′* and *x″*), as illustrated, with distances along each coordinate reduced from 5.5 to 2.5 nm in 0.05 nm steps. Each generated structure was solvated (MS and PBC), optimized (gradient 1.5 kcal/mol·Å), and analyzed. The re-optimized structures (energy gradient threshold 0.5 kcal/mol·Å) were subsequently used as initial conformations for 1 ns molecular dynamics (MD) simulations in aqueous solution

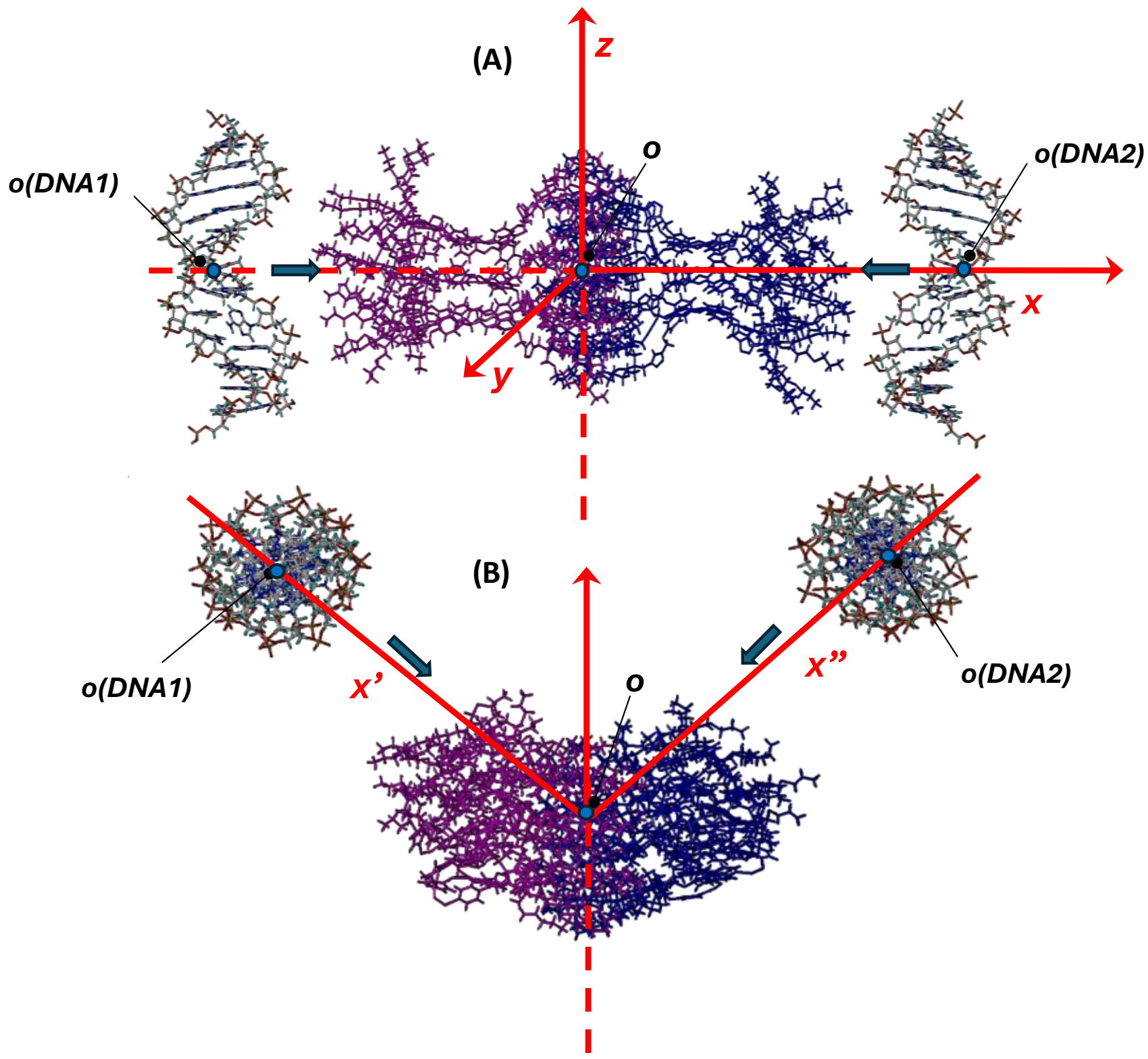

Figure S41. Schematic illustration of the coordinate system used for the simultaneous approach of two DNA fragments to the three-dimensional 3 × 3 cationic bilayer structures of **1**. The DNA fragments interact with the bilayer via their major groove. (A) all-*E* configuration: DNA fragments approach along the main *x*-axis. (B) all-*Z* configuration: DNA fragments approach along two separate virtual axes (x′ and x″).

Figure S42 shows the total 3 × 3 bilayer–DNA interaction energy, along with the van der Waals and electrostatic contributions, as well as the total energy and the non-bonded van der Waals and electrostatic components, plotted as a function of the average distances between the centre of mass of the DNA1 and DNA2 fragments and the centre of mass of the 3 × 3 bilayer's hydrophobic chains, which is located at the origin of the coordinate system in Figure S41). The bilayer–DNA interaction energies decrease as the DNA fragments approach the bilayer,

regardless of whether the azobenzene groups are in the all-*E* or all-*Z* configuration. As expected, the main contribution to this decrease arises from electrostatic interactions. The van der Waals contribution also decreases slightly, although this reduction is minimal compared to the electrostatic contribution. Notably, beyond a certain distance the strain energy of both the bilayer and the DNA fragments increases significantly—especially when the bilayer is built with azobenzenes in the *all-Z* configuration. The structures indicated by arrows in Figure S42 represent compromise situations. These are stable, low-energy structures in which the strain energy remains relatively low. Once optimized (with an energy gradient threshold of 0.5 kcal/mol·Å), the $MBE_{3\times3}/(DNA)_2$ structures were used as initial conformations for 1 ns molecular dynamics (MD) simulations in aqueous solution to assess their stability before and after photoisomerization.

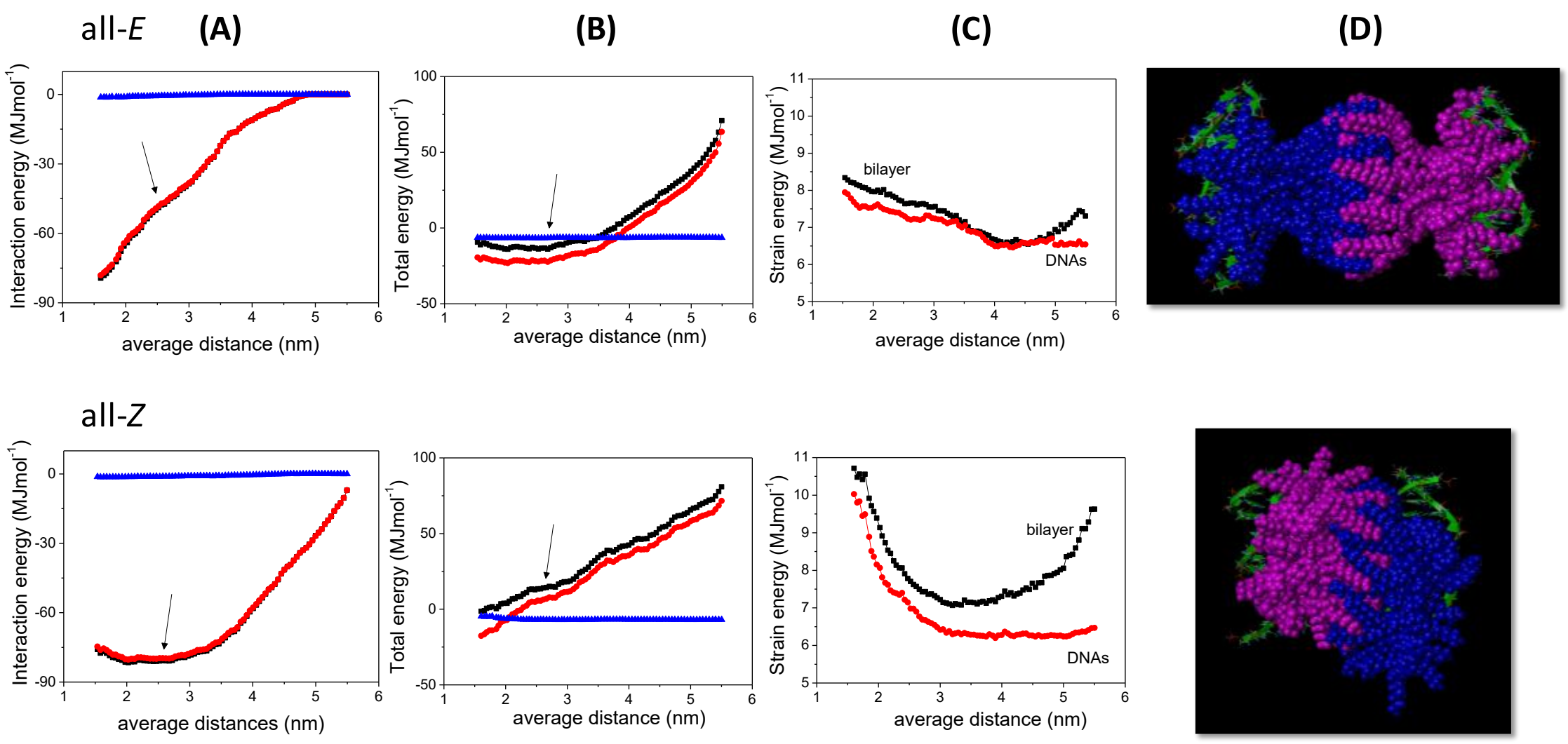

Figure S42. (A) Interaction energy (black), along with its electrostatic (red) and van der Waals (blue) components, between the DNA fragments and the all-*E* and all-*Z* 3 × 3 bilayer, plotted as a function of the average of the distance between the centre of mass of the DNA fragment, *o*(DNA1)-*o* and *o*(DNA2)-*o*, and the center of mass of the bilayer's hydrophobic terminal chains. (B) Same as in (A), showing the total energy and its electrostatic and van der Waals components, using the same colour scheme. (C) Strain energy of both the DNA fragments (DNAs; red) and the bilayer (black). (D) Selected $MBE_{3\times3}/(DNA)_2$ molecular assemblies (indicated by arrows in A and B) in both the *all-E* (upper panel) and *all-Z* (lower panel) configurations.

Effect of photoisomerization in the stability of all-*E* and all-*Z* 3 × 3 bilayers of Azo-IAJD 1 as determined by MD.

Initially, 1 ns molecular dynamics (MD) simulations were performed on the optimized $MBE_{3\times3}$ bilayer structures shown in Figure S40, each containing 18 molecules of **1** in either the all-*E* or all-*Z* configuration. Additional MD runs were carried out on 3 × 3 bilayers in which nine azobenzene units were converted to the opposite configuration and the resulting structures re-optimized, to mimic partial photoisomerization of the Azo-IAJD units.

Figures S43A and S44A show the time evolution of the total interaction energy between monolayers, together with its van der Waals and electrostatic components, for the all-*E* and all-*Z* bilayers, respectively. In both cases, the interactions are initially favourable and remain so throughout the 1 ns trajectories. The interaction energy is almost entirely dominated by van der Waals contributions, with electrostatic terms much weaker but still stabilizing. The evolution of the monolayer–monolayer distance, defined as the average distance between the centers of mass of the two nitrogen atoms in each pair of facing azobenzene units, indicates that this separation remains essentially constant over time (Figures S43C and S44C), confirming that both all-*E* and all-*Z* 3 × 3 bilayers retain their structural integrity during the simulations. Upon modelling partial photoisomerization, the total monolayer interaction energy progressively decreased (Figures 43B and 44B), in some cases approaching zero, while the monolayer–monolayer distance increased (Figures S43D and S44D). This behaviour—most pronounced for the partially switched 3 × 3 *E*-configured bilayer—signals a loss of cohesive interactions and partial disruption of bilayer integrity. The structures labelled as (2) in the right panels of Figures S43 and S44 illustrate the resulting deterioration of lamellar order, in clear contrast to the well-organized architectures of the fully ordered all-*E* and all-*Z* bilayers (structures labelled as (1)).

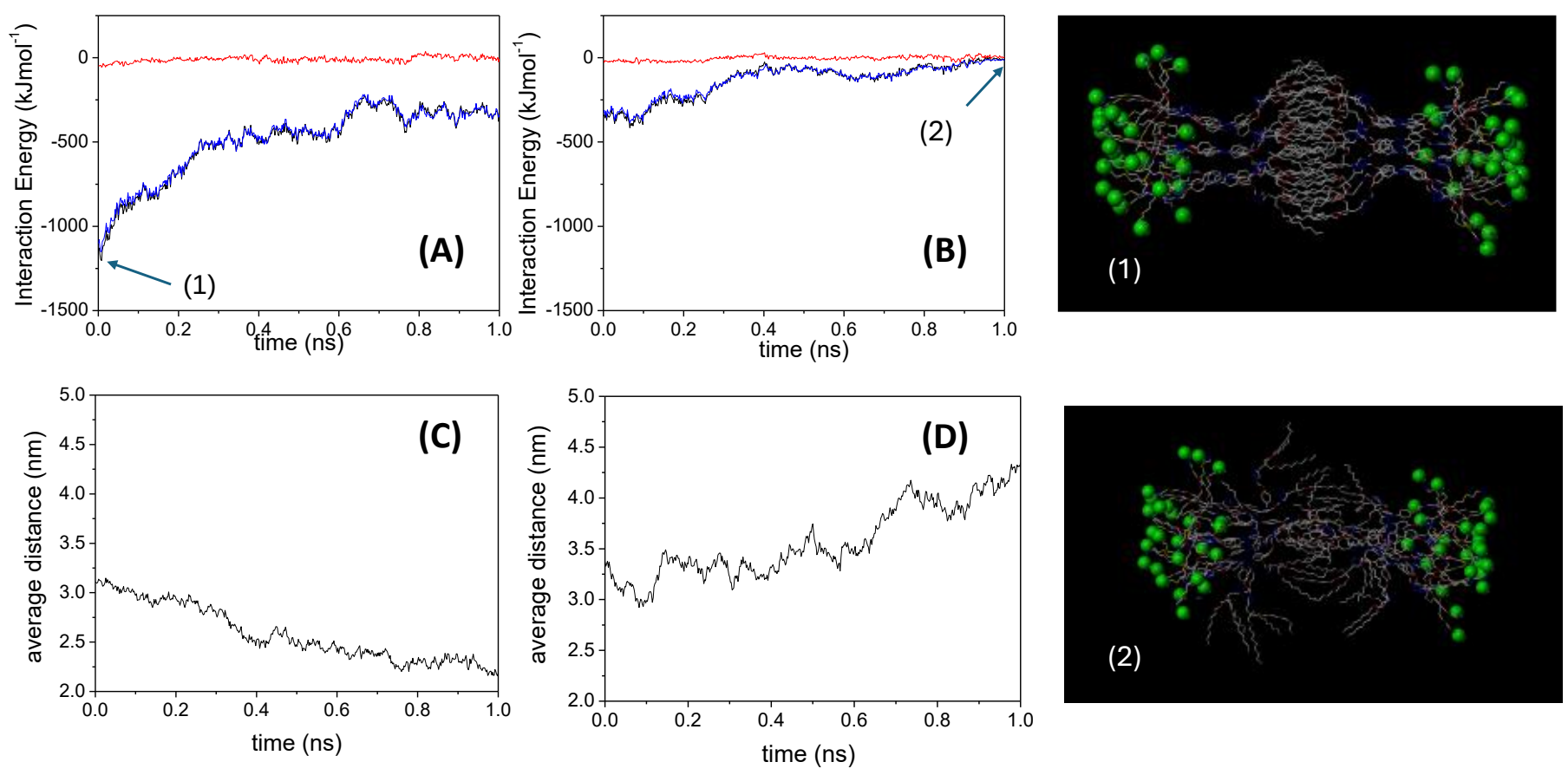

Figure S43. (A, B) Time evolution of the total interaction energy (black), together with its electrostatic (red) and van der Waals (blue) components, between the two monolayers forming the all-*E* 3 × 3 bilayer. (C, D) Time evolution of the average *o*–*o*′ distance, defined as the distance between the centers of mass of the two nitrogen atoms in each pair of facing azobenzene units within the bilayer. Data were obtained from the analysis of 1 ns MD trajectories in explicit water, using optimized $MBE_{3\times3}$ bilayer structures containing eighteen azobenzene units, either in the all-*E* configuration (A, C) or after partial photoisomerization, with nine units converted to the *Z* configuration and re-optimized (B, D). The right panel compares the initial structure (1), with all Azo-IAJD units in the *E* configuration, and the final structure after 1 ns (2), in which nine azobenzene units have undergone *E*→*Z* isomerization. Hydrogen atoms are omitted for clarity.

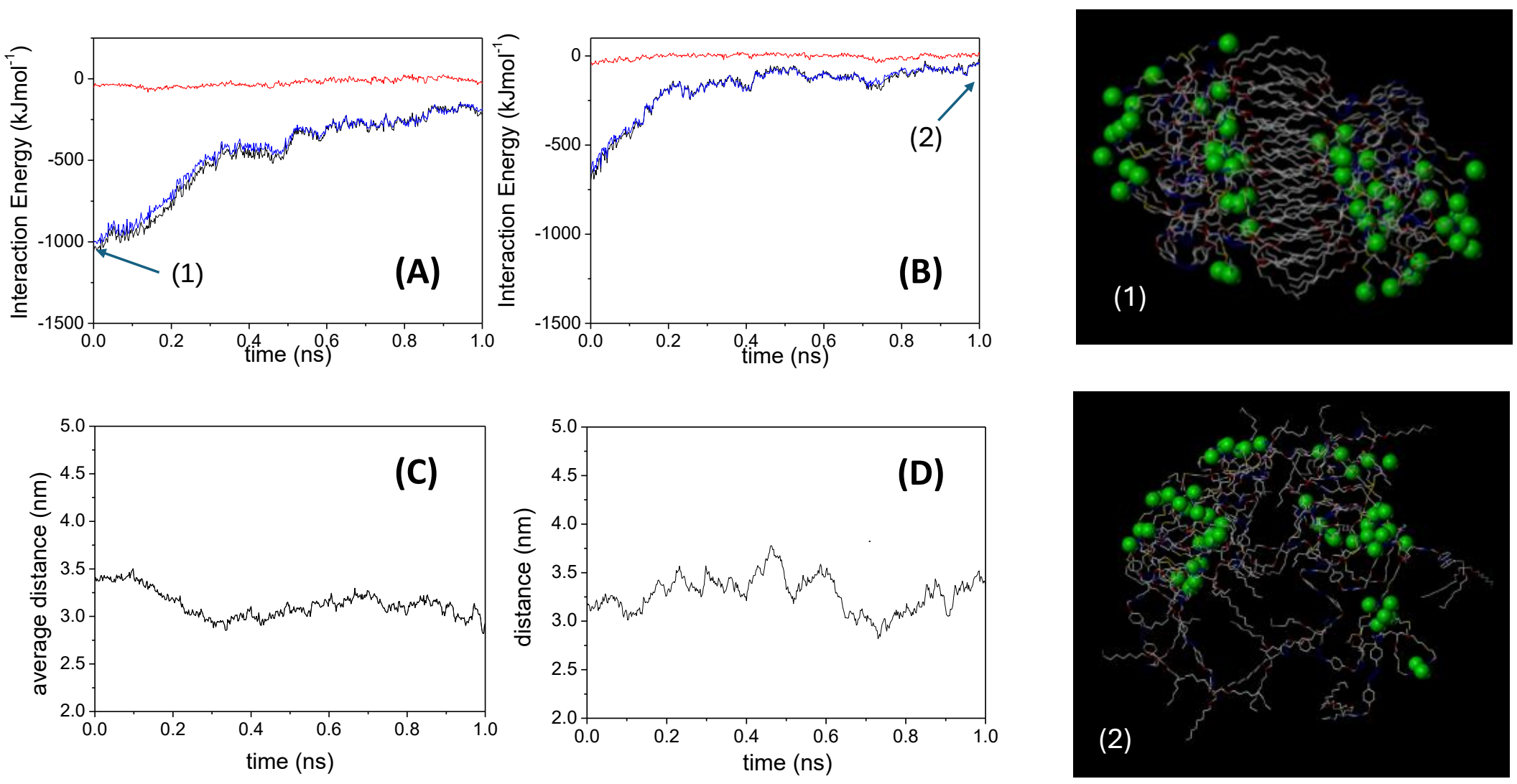

Figure S44. (A, B) Time evolution of the total interaction energy (black), together with its electrostatic (red) and van der Waals (blue) components, between the two monolayers forming the all-*Z* 3 × 3 bilayer. (C, D) Time evolution of the average *o*–*o*′ distance, defined as the distance between the centers of mass of the two nitrogen atoms in each pair of facing azobenzene units within the bilayer. Data were obtained from the analysis of 1 ns MD trajectories in explicit water, using optimized $MBE_{3\times3}$ bilayer structures containing eighteen azobenzene units, either in the all-*Z* configuration (A, C) or after partial photoisomerization, with nine units converted to the *E* configuration and re-optimized (B, D). The right panel compares the initial structure (1), with all Azo-IAJD units in the *Z* configuration, and the final structure after 1 ns (2), in which nine azobenzene units have undergone *Z*→*E* isomerization. Hydrogen atoms are omitted for clarity.

Similar MD simulations were performed on optimized 3 × 3 bilayers, in which twelve of the eighteen azobenzene units were switched to the opposite configuration, to further assess the impact of more extensive photoisomerization on bilayer stability. In this case, the destabilization is more pronounced: whatever the starting configuration, all-*E* or all-*Z*, bilayer disruption occurs early in the MD trajectory, the monolayer interaction energies rapidly approach zero, and the average of the eight *o*–*o*′ distances between monolayers increases significantly (Figure S45). This destabilization is slightly attenuated when the bilayer is initially in the all-*Z* configuration, as already observed for the less extensive photoisomerization scenario described above. Snapshots at 0 and 1 ns in Figure S46 illustrate the structural evolution of both bilayers at the beginning and end of the MD trajectories.

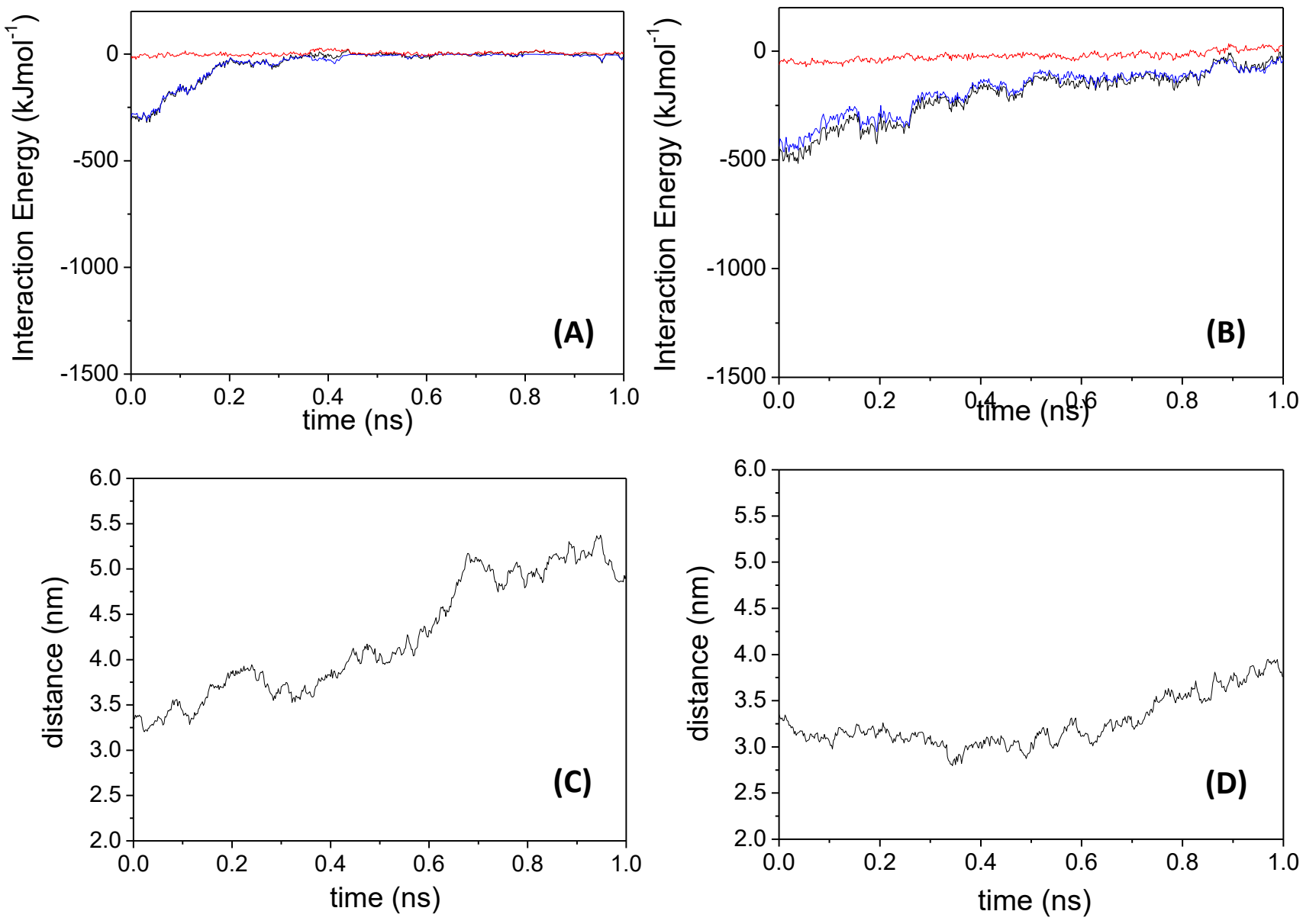

Figure S45. (A, B) Time evolution of the total interaction energy (black), together with its electrostatic (red) and van der Waals (blue) components, between the two monolayers forming the all-*E* and all-*Z* 3 × 3 bilayer, respectively, after partial photoisomerization, with twelve of the eighteen azobenzene units converted to the opposite configuration and subsequently re-optimized. (C, D) Corresponding time evolution of the average *o*–*o*′ distance, defined as the distance between the centres of mass of the two nitrogen atoms in each pair of facing azobenzene units within the bilayer. Data were obtained from the analysis of 1 ns MD trajectories in explicit water, using optimized $MBE_{3\times3}$ bilayer structures containing eighteen azobenzene units.

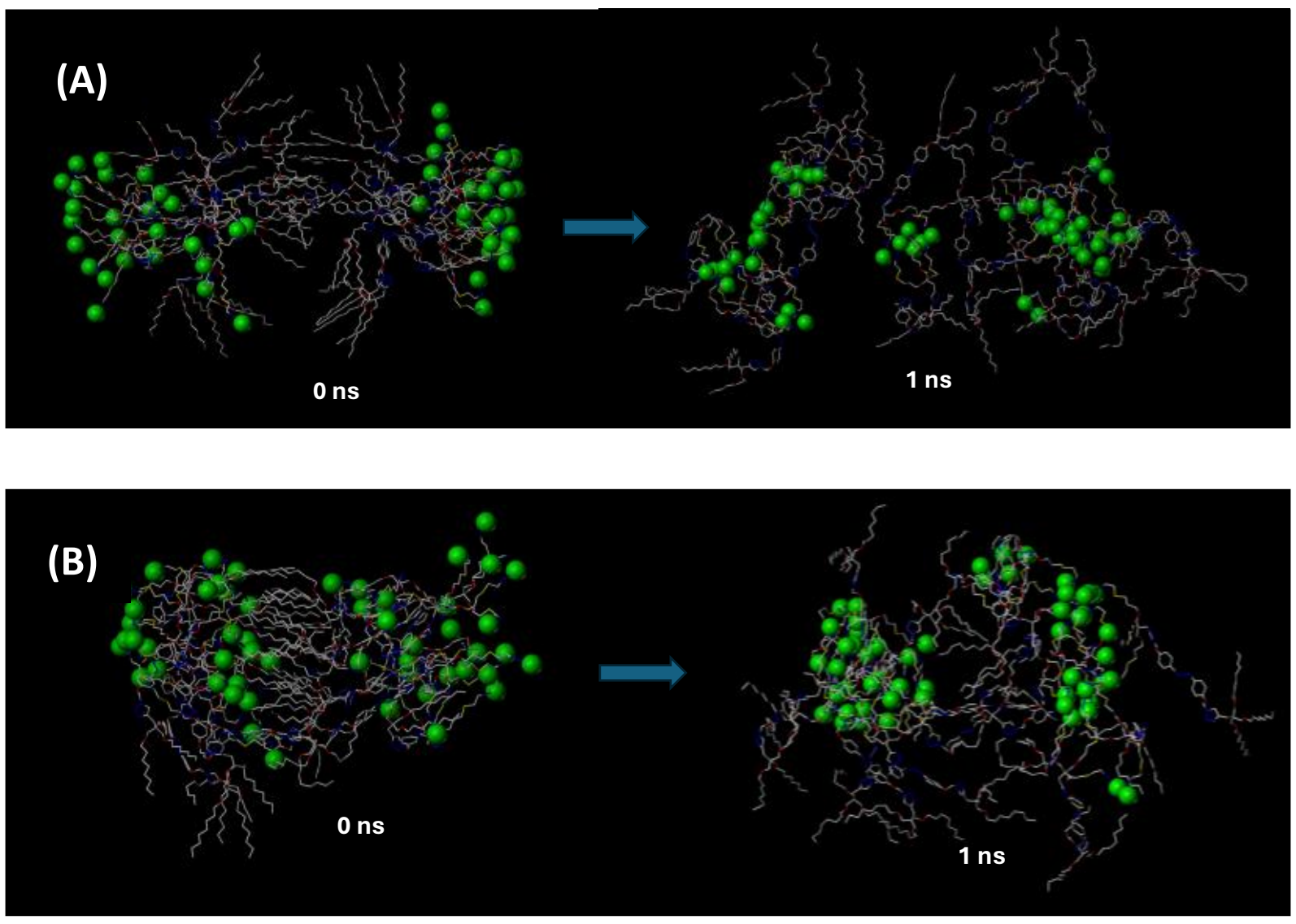

Figure S46. (A) Structure of the 3 × 3 bilayer in which twelve of the eighteen azobenzene units from the fully ordered all-*E* $MBE_{3\times3}$ bilayer were converted to the opposite configuration, shown at 0 ns and after 1 ns of the MD simulation. (B) Analogous structures obtained starting from the fully ordered all-*Z* $MBE_{3\times3}$ bilayer, shown at 0 ns and after 1 ns of the MD simulation. Hydrogen atoms have been omitted for clarity.

Assessment of the stability of 3 × 3 Azo-IAJD 1 bilayers upon complexation with two DNA fragments by MD simulations.

MD simulations of 1 ns were performed on the optimized molecular assemblies formed by the 3 × 3 bilayers of Azo-IAJD **1** and two DNA fragments shown in Figure S41, each containing 18 azobenzene moieties in either the *all-E-* or *all-Z*-configurations. Figure S47A and C shows the time evolution of the interaction energies between the DNA fragments and the charged bilayer, as well as the DNA–DNA interaction energies, for both supramolecular assemblies with the bilayer in either the all-*E* or all-*Z* configuration. In all cases, the interactions were initially favourable and remained so throughout the simulations. As expected, the interaction energies were dominated by electrostatic contributions (not shown). These results confirm that both $(DNA)_2$–bilayer assemblies remain stable irrespective of the initial azobenzene configuration. Figure S47 B and D display the time evolution of the average of the *o*–*o′* distances (black), defined between the centres of mass of the two nitrogen atoms in each pair of facing azobenzene units within the bilayer, together with the center-of-mass distance between the two DNA fragments, *o*(DNA1)–*o*(DNA2). The data indicate that the bilayer structure—and therefore the nanocomplex as a whole—remains stable throughout the trajectory. Notably, although the assemblies remain intact, the interaction energies between the positively charged monolayers are, as anticipated, strongly repulsive along the entire trajectory, reflecting dominant bilayer-DNA electrostatic forces. The two DNA fragments, in turn, reside at separations that preclude direct interaction, consistent with the energy trends shown in Figure S47A and C. The right panels of Figure S47 present representative snapshots of the 3×3 bilayer–$(DNA)_2$ nanocomplexes, with the bilayer in either the all-*E* (labelled 1 and 2) or all-*Z* configuration (labelled 3 and 4), captured at the beginning and end of the MD simulations.

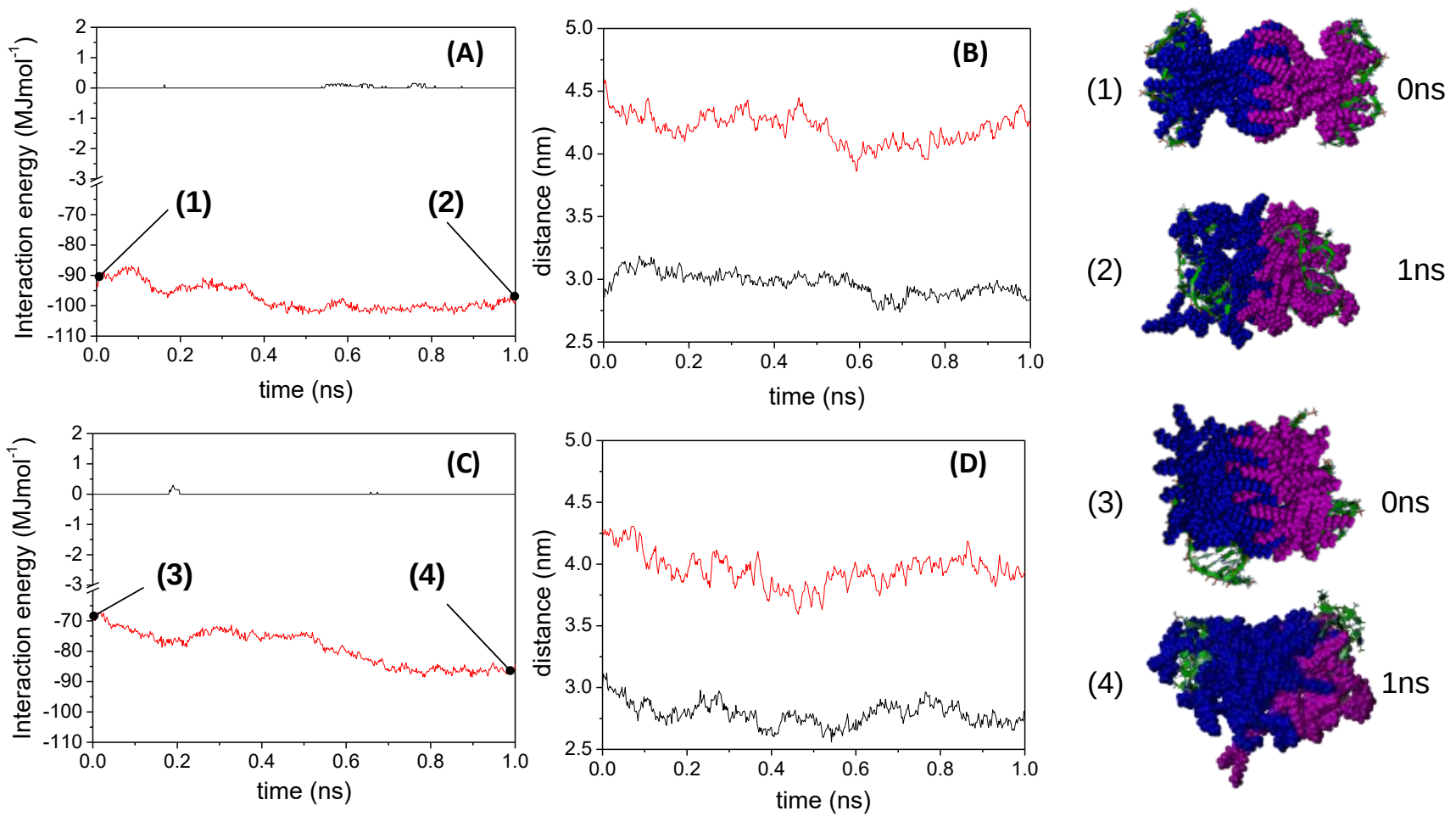

Figure S47. (A,C) Time evolution of the total interaction energy between the DNA fragments and the 3×3 bilayer of Azo-IAJD **1** (red), and between the DNA1–DNA2 fragments (black),

obtained from 1 ns MD trajectories in explicit water for the optimized 3×3 bilayer–$(DNA)_2$ nanocomplexes shown in Figure S41, with the bilayer in either the all-*E* or all-*Z* configuration, respectively. (B,D) Time evolution of the average of the *o*–*o′* distances (black), defined as the distances between the centres of mass of the two nitrogen atoms in each pair of facing azobenzene units within the bilayer, together with the centre-of-mass distance between the two DNA fragments *o*(DNA1)–*o*(DNA2), all-*E* and all-*Z* configuration, respectively. The right panels show representative snapshots of the 3×3 bilayer–$(DNA)_2$ nanocomplexes in the all-*E* (labelled 1 and 2) and all-*Z* configuration (labelled 3 and 4) at the beginning and at the end of the MD trajectories. Hydrogen atoms have been omitted for clarity.

[1] A. Lubineau, A. Malleron and C. Le Narvor, *Tetrahedron Lett.*, **2000**, *41*, 8887-8891.

[2] A. Srivastava, S. Grewal, N. Bari, M. Saraswat, S. Sinha, S. Venkataramani, *Org. Biomol. Chem*., **2022**, *20*, 5284-5292.

[3] J. M. Benito, M. Gómez-García, C. Ortiz Mellet, I. Baussanne, J. Defaye and J. M. García Fernández, *J. Am. Chem. Soc.*, **2004**, *126*, 10355-10363.

[4] J. P. Henichart, J. L. Bernier, R. Houssin. *Synthesis*, **1980**, *4*, 311-312.

[5] A. Díaz-Moscoso, L. Le Gourriérec, M. Gómez-García, J. M. Benito, P. Balbuena, F. Ortega-Caballero, N. Guilloteau, C. Di Giorgio, P. Vierling, J. Defaye, C. Ortiz Mellet, J. M. García Fernández, *Chem. Eur. J.*, **2009**, *15*, 12871-12888.

[6] M. Clark, R. D. Cramer III and N. Van Opdenbosch, *J. Comput. Chem.*, 1989, **10**, 982-1012.

[7] M. J. Frisch, G. W. Trucks, H. B. Schlegel, G. E. Scuseria, M. A. Robb, J. R. Cheeseman, G. Scalmani, V. Barone, G. A. Petersson, H. Nakatsuji, X. Li, M. Caricato, A. V. Marenich, J. Bloino, B. G. Janesko, R. Gomperts, B. Mennucci, H. P. Hratchian, J. V. Ortiz, A. F. Izmaylov, J. L. Sonnenberg, D. Williams-Young, F. Ding, F. Lipparini, F. Egidi, J. Goings, B. Peng, A. Petrone, T. Henderson, D. Ranasinghe, V. G. Zakrzewski, J. Gao, N. Rega, G. Zheng, W. Liang, M. Hada, M. Ehara, K. Toyota, R. Fukuda, J. Hasegawa, M. Ishida, T. Nakajima, Y. Honda, O. Kitao, H. Nakai, T. Vreven, K. Throssell, J. A. Montgomery Jr., J. E. Peralta, F. Ogliaro, M. J. Bearpark, J. J. Heyd, E. N. Brothers, K. N. Kudin, V. N. Staroverov, T. A. Keith, R. Kobayashi, J. Normand, K. Raghavachari, A. P. Rendell, J. C. Burant, S. S. Iyengar, J. Tomasi, M. Cossi, J. M. Millam, M. Klene, C. Adamo, R. Cammi, J. W. Ochterski, R. L. Martin, K. Morokuma, O. Farkas, J. B. Foresman and D. J. Fox, *Gaussian 09*, Revision A, Gaussian, Inc., Wallingford CT, 2009.

[8] M. Blanco, *J. Comput. Chem.*, **1991**, *12*, 237-247.

[9] Y. Brunel, H. Faucher, D. Gagnaire and A. Rassat, *Tetrahedron*, **1975**, *31*, 1075-1091.

[10] W. H. Press, S. A. Teukolski, W. T. Vetterling and B. P. Flannery, *Numerical Recipes: The Art of Scientific Computing*, 3rd edn, Cambridge University Press, Cambridge, 2007.

[11] M. Martínez-Negro, N. Sánchez-Arribas, A. Guerrero-Martínez, M. L. Moyá, C. Tros de Ilarduya, F. Mendicuti, E. Aicart and E. Junquera, *Pharmaceutics*, **2019**, *11*, 632.

[12] L. Gallego Yerga, L. Blanco Fernández, K. Urbiola, T. Carmona, G. Marcelo, J. M. Benito, F. Mendicuti, C. Tros de Ilarduya, C. Ortiz Mellet, J. M. García Fernández, *Chem. Eur. J.*, **2015**, *21*, 12093-12104.

[13] M. J. González-Álvarez, P. Balbuena, C. Ortiz Mellet, J. M. García Fernández, F. Mendicuti, *J. Phys. Chem. B*, **2008**, *112*, 13717-13729.

[14] M. J. González-Álvarez, J. Vicente, C. Ortiz Mellet, J. M. García Fernández, F. Mendicuti, *J. Fluoresc.*, **2009**, *19*, 975-988.
